# Supplementary material for: Microcavity-like exciton-polaritons can be the primary photoexcitation in bare organic semiconductors
Source: Nat Commun. 2021 Nov 11;12:6519. doi: 10.1038/s41467-021-26617-w (PMC8585971; doi:10.1038/s41467-021-26617-w)
Supplement: Supplementary file 1 — Supplementary Information [file 41467_2021_26617_MOESM1_ESM.pdf]

Microcavity-Like Exciton-Polaritons can be the Primary Photoexcitation in Bare Organic Semiconductors

[1]Raj Pandya, [1]Richard Y. S. Chen, [1]Qifei Gu, [1]Jooyoung Sung, [1]Christoph Schnedermann, [1]Oluwafemi S. Ojambati, [1]Rohit Chikkaraddy, [1]Jeffrey Gorman, [2]Gianni Jacucci, [2]Olimpia D. Onelli, [3]Tom Willhammar, [4]Duncan N. Johnstone, [4]Sean M. Collins, [4]Paul A. Midgley, [1]Florian Auras, [1]Tomi Baikie, [5]Rahul Jayaprakash, [6]Fabrice Mathevet, [7]Richard Soucek, [8]Matthew Du, [1]Antonios M. Alvertis, [1]Arjun Ashoka, [2]Silvia Vignolini, [5]David G Lidzey, [1]Jeremy J. Baumberg, [1]Richard H. Friend, [7]Thierry Barisien, [7]Laurent Legrand, [7]Alex W. Chin, [8]Joel Yuen-Zhou, [9,10]Semion K. Saikin, [11]Philipp Kukura, [12]Andrew J. Musser, [1]Akshay Rao*

[1]Cavendish Laboratory, University of Cambridge, J.J. Thomson Avenue, CB3 0HE, Cambridge, United Kingdom

[2]Department of Chemistry, University of Cambridge, Lensfield Road, Cambridge CB2 1EW, United Kingdom

[3]Department of Materials and Environmental Chemistry, Stockholm University, Stockholm, Sweden

[4]Department of Materials Science and Metallurgy, University of Cambridge, 27 Charles Babbage Road, CB3 0FS, Cambridge, United Kingdom

[5]Department of Physics & Astronomy, University of Sheffield, S3 7RH, Sheffield, United Kingdom

[6]Sorbonne Université, CNRS, Institut Parisien de Chimie Moléculaire (IPCM) UMR 8232, Chimie des Polymères, 4 Place Jussieu, 75005 Paris, France

[7]Institut des NanoSciences de Paris, UMR 7588/CNRS, Université Pierre et Marie Curie (Paris 6), Campus Boucicaut, 140 rue de Lourmel, 75015 Paris, France

[8]Department of Chemistry and Biochemistry, University of California San Diego, La Jolla, California 92093, United States

[9]Department of Chemistry and Chemical Biology, Harvard University, 12 Oxford Street, Cambridge, Massachusetts 02138, United States

[10]Kebotix Inc., 501 Massachusetts Avenue, Cambridge, Massachusetts 02139, United States

[11]Physical and Theoretical Chemistry Laboratory, Department of Chemistry, University of Oxford, South Parks Road, Oxford OX1 3QZ, UK.

[12]Department of Chemistry and Chemical Biology, Cornell University, Baker Laboratory, Ithaca, NY, 14853, U.S.A

*correspondence: ar525@cam.ac.uk

Supplementary Material

Table of Contents

Supplementary Methods 4
Supplementary Note 1: Sample characterization 19
Cyclohexyl PDI crystallographic mapping by scanning electron diffraction 20
Scanning electron microscopy of PDA and PIC thin films 22
Atomic force microscopy of PDI nanobelts 23
Transmission electron microscopy of PIC 24
Kelvin probe force microscopy 25
Confocal Microscopy 25
Photoluminescence microscopy of PDI nanobelts 26
Photoluminescence microscopy of PIC thin films 26
Supplementary Note 2: Specular reflection 30
Specular reflection measurements on PIC 30
Specular reflection measurements on PDA 31
Absorption and Reflection spectra of PDI 33
Supplementary Note 3: Transfer Matrix Simulations 35
Supplementary Note 4: MEEP Calculations 40
Supplementary Note 5: Rabi Flopping measurements 48
Supplementary Note 6: Data analysis 51
Data acquisition 51
Self-referencing method 51
Data smoothing - moving average 53
Accurate determination of time zero, signal centre and sigma propagation 57
General method 57
Determination the signal centre 60
Determine sigma at different time points and angles 62
Extraction of time zero (t_0) 63
Spot size at time zero 65
Radial analysis of TAM kinetics 67
Fitting with two-dimensional Gaussian function 70
Supplementary Note 7: Probe wavelength dependence 80
Femtosecond pump-probe spectra of PDA 80

Femtosecond pump-probe spectra of PIC 81
Femtosecond pump-probe spectra of PDI nanobelts 83
Kinetics of fs-TAM signal 85
Supplementary Note 8: Modelling coherent transport in fs-TAM data 92
Supplementary Note 9: Modelling annihilation in fs-TAM data 96
Pump fluence dependence 99
Supplementary Note 10: Correlation between diffusion length and decay time and error bars on MSD 102
Supplementary Note 11: Theoretical modelling of exciton diffusion in PDI, PIC and PDA 108
Ab initio calculations of lowest electronic transitions and transition densities of PDI molecules 108
Intermolecular couplings and exciton transport in PDI nanobelts 109
Theoretical calculation of exciton velocities in PDA 113
Supplementary Note 12: Waveguiding 115
Supplementary Note 13: Control measurements 118
Supplementary Note 14: Refractive index dependent fs-TAM measurements 123
Supplementary References 124

Supplementary Methods

Femtosecond Transient Absorption Microscopy (fs-TAM) Our fs-TAM setup is schematically depicted in Supplementary Figure 1. Pulses were delivered by a Yb:KGW amplifier (Pharos, LightConversion, 1030 nm, 5 W, 200 kHz) that seeded two broadband white light (WL) stages . The probe WL was generated in a 3 mm YAG crystal and adjusted to cover the wavelength range from 650-950 nm by a fused-silica prism-based spectral filter. In contrast, the pump WL was generated in a 3 mm sapphire crystal to extend the WL in the high frequency to 500 nm, and a short-pass filtered at 650 nm (Thorlabs, FESH650).

The pump pulses were focussed onto the sample using a single-lens oil immersion objective (100×, numerical aperture 1.1 NA) to a diffraction-limited spot of ~270 nm (FWHM, full bandwidth). In contrast, the counter-propagating probe pulses were loosely focused onto the sample by a concave mirror (FWHM ~15 μm).

A set of third-order corrected chirped mirrors (pump WL – Layertec, probe WL – Venteon) in combination with a pair of fused silica wedge prisms (Layertec) compressed the pulses to sub-10 fs at the sample, as verified by second-harmonic generation frequency-resolved optical gating (Figure **SI2**).

The transmitted probe light was collected by the same objective used to focus the pump pulses and imaged by an EMCCD camera (Qimaging Rolera Thunder, Photonmetrics). Pump light was suppressed by inserting a 650 nm long-pass filter in the detection path (Thorlabs, FELH650) in front of the camera. Additionally, a bandpass filter (Semrock/Thorlabs) was additionally placed in front of the camera to image at the desired probe wavelength in order to avoid chromatic aberration induced image artefacts. Differential imaging was achieved by modulating the pump beam at 45 Hz by a mechanical chopper. The axial focus position was maintained by an additional auto-focus line based on total internal reflection of a 405 nm continuous wave laser beam.

The TAM method builds on techniques developed by the super-resolution microscopy community where monitoring a combination of 'on' and 'off' states allows for sub-diffraction imaging When there is no time delay between pump and probe, a diffraction limited transient absorption (TA) signal is imaged onto an EMCCD camera arising only from the area of excitation. At subsequent time delays, any transport of the initial excitation energy will be observed as a spreading of the spatial pump-probe signal, outside of the initial excitation spot. Subtracting the FWHM of our spatial signals at different time delays allows read-out of the extent of transport as a function of time. Because we are subtracting spatial signals at different time delays, the only limit to our resolution is how well *different* spatial profiles can be resolved. Based on the signal to noise ratio of the measurement this is typically ~10 nm, and hence allows us to break through the diffraction limit.

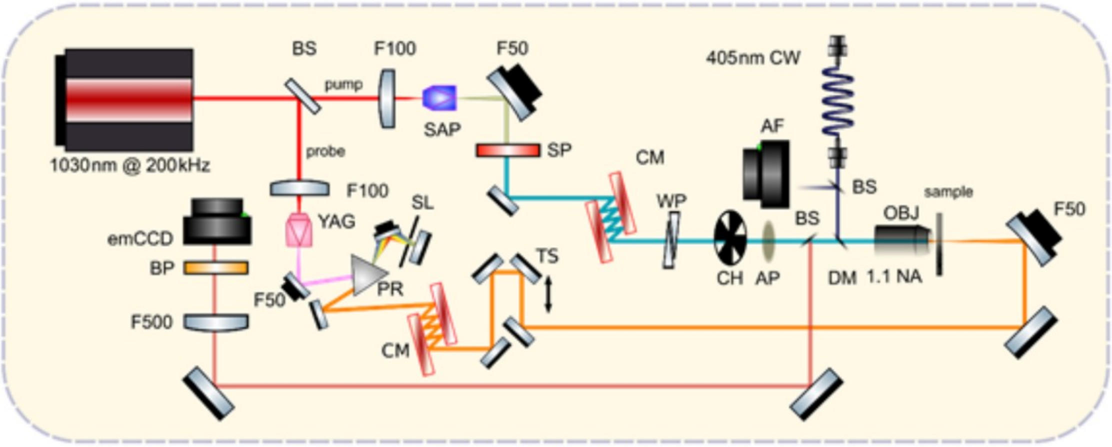

Supplementary Figure 1: Schematic of fs-TAM setup. Pump and probe pulses are generated by focussing the 1030 nm fundamental of the laser into YAG and sapphire crystals to generate a white light continuum. After second and third order dispersion compensation with chirp mirrors and fused silica wedge pairs, the pump is focussed onto the sample by a high NA objective whilst the probe is loosely focussed onto the sample to achieve wide field illumination. A chopper generates a series of pump on and off shots, which are subsequently imaged by the EMCCD camera. BS – beam splitter, FX – focussing or collimating lens/mirror of X focal length in cm, CM – chirp mirrors, WP – silica wedge pair, AP – pinhole aperture, OBJ – objective, BP X band pass filter of X nm width, SP – short pass filter at X nm, CH – chopper wheel, YAG – 3 mm yttrium aluminium garnet, SAP – 3 mm sapphire, DM – dichroic mirror, AF – auto focus camera, CW – continuous wave autofocus laser, PR – prism, SL – slit, TS – translation stage, EMCCD - electron multiplying charge coupled device.

Pump and probe pulse compression

The pump and probe pulse compression was confirmed using second harmonic generation frequency resolved optical gating (SHG-FROG) [1]. The dispersion of each pulse up to the sample was compensated for with a combination of chirp mirrors and silica wedge pairs. For the pump, the SHG-FROG traces were measured following passing through the objective. Supplementary Figure 2 shows the respective SHG-FROG traces confirming a sub-10 fs pulse compression.

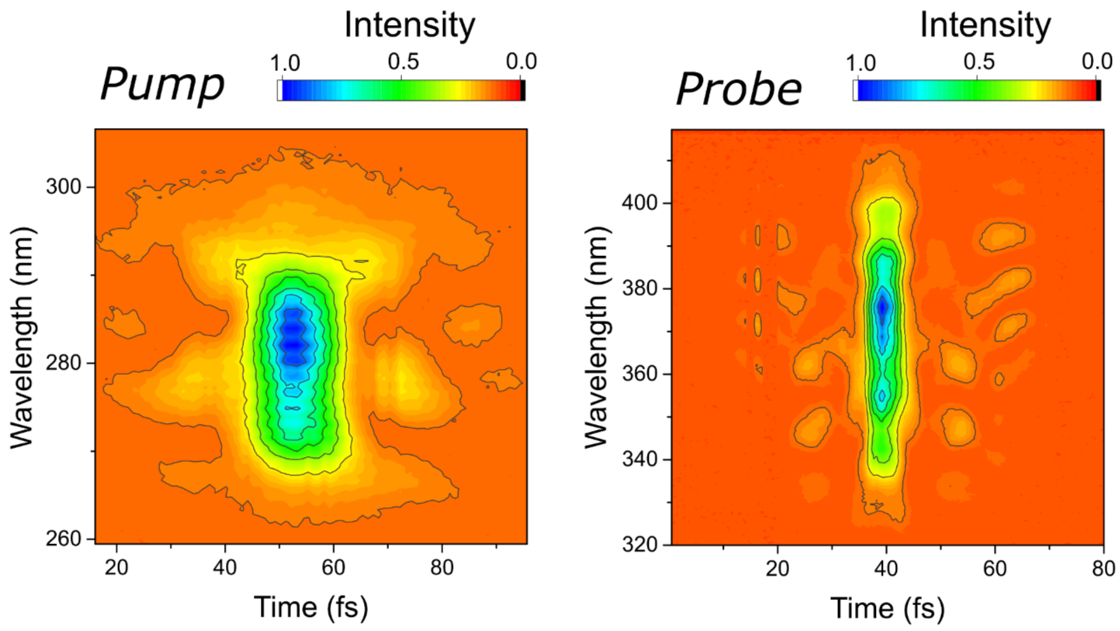

Supplementary Figure 2: a-b. Second harmonic generation frequency resolved optical gating (SHG-FROG) traces for pump and probe. The step size used is 1 fs and the pump FROG spectrum is taken after the beam has passed through all dispersive optics including the objective. In this case the pump has time duration of ~8 fs and probe ~6 fs.

The time resolution of our setup can also be confirmed from measurement of the samples themselves. PDA for example, is known to exhibit strong Raman bands at ~1400 cm^{-1} and ~2100 cm^{-1} arising from double and triple bond stretches, respectively. A sufficiently short laser pulse (<15 fs) should be able to impulsively excite these modes, resulting in an oscillatory modulation superimposed on the pump probe kinetics. As can be seen in Supplementary Figure 3a this is the case for the 670 nm kinetic. Subtracting the electronic background and Fourier transforming to the frequency domain shows the kinetic contains two modes: one at 1420 cm^{-1} and another at 2080 cm^{-1}, verifying the high temporal resolution of our setup.

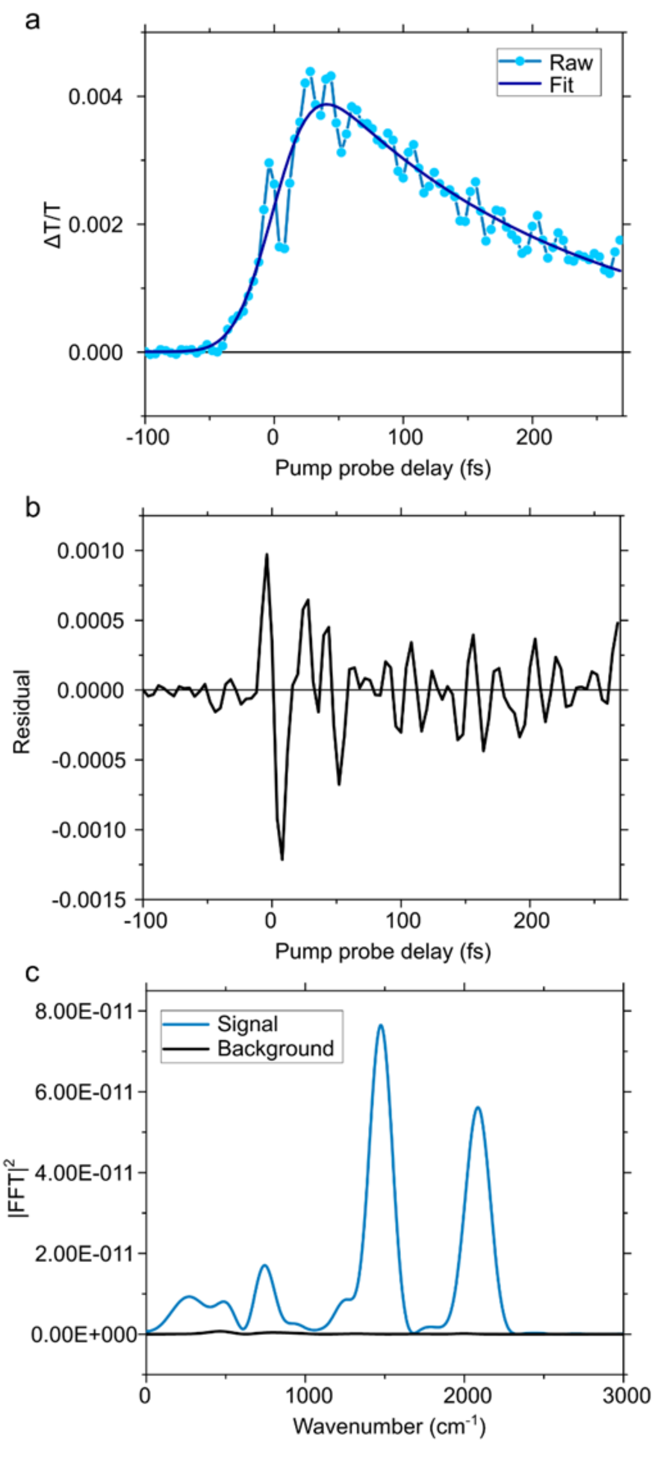

Supplementary Figure 3: a. PDA kinetic at 670 nm, solid dark blue line shows fit to the raw data (light blue line). **b.** Residual following subtraction of electronic decay. **c.** Fourier transform of residual (blue line) showing clear Raman modes at 1480 cm^{-1} and 2080 cm^{-1}. Dark black line shows Fourier transform of the background.

Femtosecond Pump Probe Spectroscopy (fs-TA) The fs-TA experiments were performed using an Yb-based amplified system (Pharos, Light Conversion) providing 14.5 W at 1030 nm and a 38 kHz repetition rate. The probe beam was generated by focusing a portion of the fundamental in a 4 mm YAG substrate and spanned from 520 to 1400 nm. The pump beam was generated in home-built non-collinear optical parametric amplifiers (NOPAs; 37° cut BBO, type I, 5° external angle) pumped with either the second or third harmonic of the source. The NOPAs output (~4–5 mW power) was centered between 520 nm - 860 nm depending on the experiment, and pulses were compressed using a chirped mirror wedge prism (Layterc) combination to a temporal duration of 9 fs - 25 fs for the particular experiment (upper limits determined by SHG-FROG). The white light probe was delayed using a computer-controlled piezoelectric translation stage (Physik Instrumente), and a sequence of probe pulses with and without pump was generated using a chopper wheel on the pump beam. The energy density per pulse maintained at 19 μJ/cm^2. After the sample, the probe pulse was split with a 950 nm dichroic mirror (ThorLabs). The visible light (520–950 nm) was then imaged with a silicon photodiode array camera (Stresing Entwicklunsbüro; visible monochromator 550 nm blazed grating) with the near-infrared proportion of the probe seeded to an IR monochromator (1200 nm blazed grating) and imaged using an InGaAs photodiode array camera (Sensors Unlimited). This technique allows simultaneous collection of the entire probe spectrum in a single shot. Offsets for the differing spectral response of the detectors was accounted for in the data post-processing.

Picosecond Pump Probe Spectroscopy (ps-TA) The picosecond transient absorption (ps-TA) experiments were performed using an Yb-based amplified system (PHAROS, Light Conversion) providing 14.5 W at 1030 nm and 38 kHz repetition rate. The probe beam was generated by focusing a portion of the fundamental in a 4 mm YAG substrate and spanned from 520 to 900 nm. The pump beam was generated by seeding a portion of the fundamental to a narrow band optical parametric oscillator (ORPHEUS-LYRA, Light Conversion). The pump and probe beams were focused to an area of 300 μm × 330 μm and 60 μm × 70 μm, respectively on the sample using off-axis parabolic mirrors. The energy density per pump pulse was 5 - 45 μJ/cm^2. The white light probe was delayed using a computer-controlled piezoelectric translation stage (Newport), and a sequence of probe pulses with and without the pump was generated using a chopper wheel (ThorLabs) on the pump beam. The probe pulse transmitted through the sample was detected by a silicon photodiode array camera (Stresing Entwicklungsbüro; visible monochromator 550 nm blazed grating).

Transmission and Reflection Spectroscopy Optical microscopy was performed using a Zeiss Axio.Scope optical microscope in Köhler illumination equipped with a 100× objective (Zeiss EC Epiplan-APOCHROMAT 0.95 HD DIC) coupled to a spectrometer (Avantes HS2048) via an optical fibre (Thorlabs, FC-UV50-2-SR). Five spectra were collected for each sample using an integration

time of 10 ms and 20 ms for reflection and transmission measurements, respectively. The reflectance and transmittance were calculated using a silver mirror (ThorLabs, PF10-03-P01) and the glass substrate as references respectively.

Angle resolved reflectivity Angle dependent reflectivity measurements were performed using an Andor Shamrock SR-303i-A triple-grating imaging spectrograph, with a focal length of 0.303m. The spectra were recorded using a 300 grooves / mm grating blazed at 500 nm. The reflectivity light source was a fibre-coupled 20W tungsten halogen lamp (Ocean Optics DH-2000-BAL). The angle dependence was measured using a k-space imaging setup. The white light was focussed on to the sample at normal incidence using an Edmund Optics 20X objective with a numerical aperture (NA) = 0.63, with the reflected signal collected through the same optical path using a beam splitter. This light was then focussed into a spectrometer using a final collection lens. An additional Fourier-plane imaging lens positioned before the final lens facilitated the Fourier plane to be imaged into the spectrometer. Here, a dual axis slit was positioned at the focus of the imaging lens (before the final collection lens) which allowed the reflectivity to be spatially filtered, permitting the unwanted real space signal to be rejected.

Power dependent photoluminescence spectroscopy Samples were excited with ~120 fs pulses, ~10 nm full width at half maximum (FWHM), generated from a tunable optical parametric oscillator (OPO) (Spectra Physics Inspire) pumped at 820 nm with a repetition rate of 80 MHz. The power of the pulses was controlled using a variable neutral density filter (ThorLabs) mounted on a rotating stage. The attenuated pulses pass were focused by a microscope objective (Nikon 100x, numerical aperture 0.9) to excite the PDI nanobelts at 590 nm. Emission light was collected by the focusing objective and passes through tunable long pass filters (Fianum). An achromatic doublet lens (focal length f = 60 mm) focuses the filtered light onto a single photon avalanche diode (SPAD) (MPD, jitter time <50 ps). The output of the SPAD was read out and averaged over 5 seconds for each power step. In the plot shown in Figure 2 of the main manuscript, we have accounted for the transmission through the optics and the data is a representative measurement from different single nanobelts.

Sample Preparation

Unless otherwise stated all samples were deposited onto pre-cleaned (Acetone/Water, Isopropanol, 10 mins O_2 Plasma etch) 22 mm × 22 mm No. 1.5 borosilicate glass coverslips (ThorLabs). Samples were encapsulated in a N_2 glovebox prior to measurement by placing a second smaller coverslip atop of the sample substrate with a ~200 µm thick carbon tape spacer and sealing with epoxy resin.

Polydiacetylene (PDA): The 3BCMU (3-methyl-n-butoxy-carbonylmethyl-urethane) diacetylene molecules were synthetized in-house using the method previously outlined by Se *et al.* and references

therein [2]. The synthesis classically consisted of two steps: (i) oxidative coupling of 4-pentyn-1-ol (Hay's method) to produce the 4,6-decadiyn-1,10-diol and then, (ii) reaction of the diol with *n*-butylisocyanate acetate. Note that the source 4-pentyn-1-ol was not synthetized at the laboratory but purchased from Sigma-Aldrich (Merck).

Ultrathin single crystals were grown between two coverslips using a melt-processing method. The whole process was systematically carried out under a polarized optical microscope so as to be able to follow and control the sample elaboration: a very small amount of diacetylene powder is placed at one edge of the double-slides assembly; when heating above the melting temperature (~65°C) the liquid diacetylene fills the empty space by capillary action to form a thin liquid film between the two substrates. Rapid cooling leads to the formation of a highly polycrystalline film. The sample is then heated again to around the melting temperature until the melting of all the crystallites took place. When only a few crystal germs remain the sample is cooled again at a very slow cooling rate (typically < 0.1°C/mn) to induce the growth of large single monocrystalline domains from the germs.

The sample prepared in this manner is then studied as it is (without separation of the slides) after the desired amount of polymer is generated inside the film. It is important noting that submicronic thickness is obtained [3] corresponding to a reduction of 1 to 2 orders of magnitude in thickness as compared with diacetylene monocrystals usually considered in advanced transmission spectroscopy experiments [4–6]. The optical density (OD) of the studied samples was kept below 10^{-2} (that is close to the detection limit of our CARY 5000 spectrophotometer). Taking an upper bound of ~800 nm for the film thickness leads to a chain content by weight, x_p, that keeps below ~ $2.3\ 10^{-4}$ [7]. It is then estimated that such a polymer content corresponds to an interchain distance, d_{inter}, of several tens of nm; for $x_p = x_{p,\,max} \sim 2.3\ 10^{-4}$ we find here $d_{inter} = d_{inter,\,min} \sim 30$ nm (considering the shorter interchain distance along the crystal **c** axis, $d_{inter} \sim 0.45$ nm, for $x_p = 1$ as well as homogeneous topochemical polymerization [7,8]). In these conditions interchain interactions can reasonably be discarded in the analysis.

Highly aligned regions of the crystal were selected using a transmission microscope prior to fs-TAM measurements. A 3BCMU crystal (monoclinic space group) is a birefringent medium with its optic axis lying in the surface plane and corresponding to the long axis of the aligned polymer chains. Another principal axis also lies in the surface plane of the crystal). To identify high quality crystalline domains (constant thickness, absence of strain), samples are placed between crossed polarisers and illuminated. The rotation of the sample allows to identify domains that present the most homogeneous optical extinction (Supplementary Figure 4). Regions exhibiting such behaviour were predefined by application of masks to the sample.

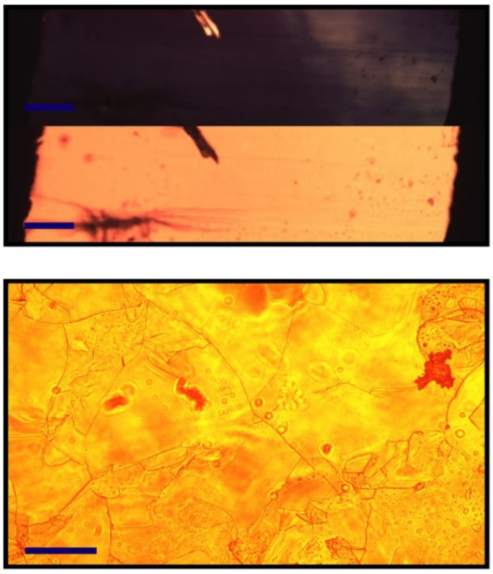

Supplementary Figure 4: Top panel: Optical microscope image of PDA thin film in transmission and between crossed polarizers. When the linearly polarized incident light has its polarization along one of the optic axes, complete extinction is obtained. In "dark" domains the chains present in the diacetylene matrix have their conjugated backbone either in the direction of incident polarization or perpendicular to it. In general, the chains are parallel to the longest dimension of the monocrystalline domain. Adjacent domains do not have the same orientation as observed in the figure and transmitted light acquires elliptical polarization as it propagates through the film. Scale bar in the top image is 2 µm. Bottom image shows transmission microscope image through PDA crystal. Clear crystal domains can be observed. Scale bar for bottom image is 150 µm.

PIC: To prepare the aggregates, we modified existing procedures [9,10] to create a high-concentration sugar glass similar to that initially detailed for double-walled cyanine nanotubes by Caram *et al.* [11]. PIC monomer (Sigma Aldrich) was dissolved in methanol by shaking overnight to produce a 10 mM solution. We then mixed 250 µL of monomer solution with 250 µL of a saturated solution of sucrose. We deposited 200 µl of the sugar-monomer mixture onto pre-cleaned glass substrates (acetone, isopropanol, O_2 plasma etc; each 10 mins) and spin coated at 2500 rpm for 2 mins. All spin coating was performed in an inert N_2 environment, to prevent oxidative degradation. All solvents used were purged of O_2 via a freeze-pump-thaw cycle and bubbling of Argon through the solvent. The samples were then dried under vacuum (0.5 atm) for 24 hrs, to form a uniform amorphous glass. Samples for electron microscopy were prepared by dropping ~3 µL of monomer-sugar solution matrix on to a TEM copper grid with a holey carbon support film in an inert atmosphere, followed by spin coating under the same conditions as above. Once dried in vacuum, bright-field TEM was carried out in an FEI Tecnai Osiris TEM operated at 200 kV. In all cases, samples were measured within 48 hrs of preparation to avoid morphological changes.

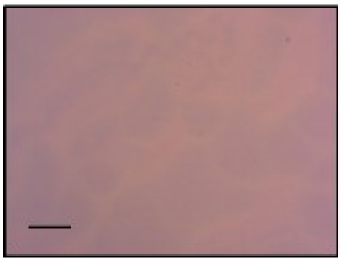

Supplementary Figure 5: Optical microscope image of PIC thin film. The fibrous pale yellow streaks on the pink background are suggested to be bundles of nanotubes. Scale bar is 5 µm.

PDI: In order to prepare PDI nanobelts, the PDI monomer was synthesised as described below.

Abbreviations

CF Chloroform

EtOAc Ethyl Acetate

EtOH Ethanol

eq. Equivalents

HCl Hydrochloric Acid

NMR Nuclear Magnetic Resonance

PTCDA Perylene - 3, 4, 9, 10 - Tetracarboxylic Dianhydride

R_f Retardation Factor

Materials

Solvents and reagents were purchased from commercial suppliers and used as received. Flash column chromatography was performed using silica gel (60 Å, 40-60 µm, Acros Organics). Analytical thin layer chromatography was carried out with silica gel 60 F254 plates (Merck).

Methods

Reactions were carried out with oven-dried glassware. ^1H and ^{13}C NMR spectra were recorded on a Bruker 500 MHz DCH Cryoprobe Spectrometer. Proton chemical shifts (δ) were measured in parts per million (ppm) relative to tetramethylsilane (0 ppm) and referenced to the residual undeuterated solvent peaks. Shifts are reported to the nearest 0.01 ppm, coupling constants (J) reported to the nearest 0.1 Hz. Multiplicity is reported using: singlet (s), doublet (d), triplet (t), quartet (q), and multiplet (m), and combinations thereof. Carbon chemical shifts are also measured in ppm relative to tetramethylsilane, referenced to the solvent peaks, and are quoted to the nearest 0.1 ppm.

Synthesis

***N*,*N′*-dicyclohexyl -3,4,9,10-perylenedicarboximide**

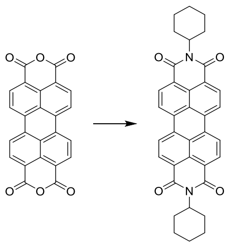

PTCDA (390 mg, 1 mmol, 1 eq.), $Zn(OAc)_2 \cdot 2H_2O$ (196 mg, 4 mmol, 4 eq.), cyclohexylamine (343 μL, 3 mmol, 3 eq.), and imidazole (4 g) were stirred under argon at 160 °C for 2 hrs. The reaction was cooled and the crude dispersed in EtOH (150 mL), 2M HCl (100 mL), and water (100 mL) and left stirred overnight in air at room temperature. The precipitate was collected by filtration, washed with water, and dried. The solid was purified by column chromatography (silica gel, CF/EtOAc, 9:1) to yield the title compound as a dark red solid (283 mg, 0.51 mmol, 51 %).

^{1}H NMR (500 MHz, $CDCl_3$, <0.5 mg/mL) δ 8.67 (d, *J* = 8.0 Hz, 4H), 8.61 (d, *J* = 8.0 Hz, 4H), 5.05 (tt, *J* = 12.2, 3.8 Hz; 2H), 2.62-2.53 (m, 4H), 1.94-1.91 (m, 4H), 1.80-1.73 (m, 6H), 1.50-1.33 (m, 6H).

^{13}C NMR (126 MHz, $CDCl_3$) δ 163.9, 134.5, 131.1, 129.4, 126.4, 123.9, 123.0, 54.0, 31.87, 23.5, 22.7.

R_f 0.54 (CF:EtOAc, 9:1)

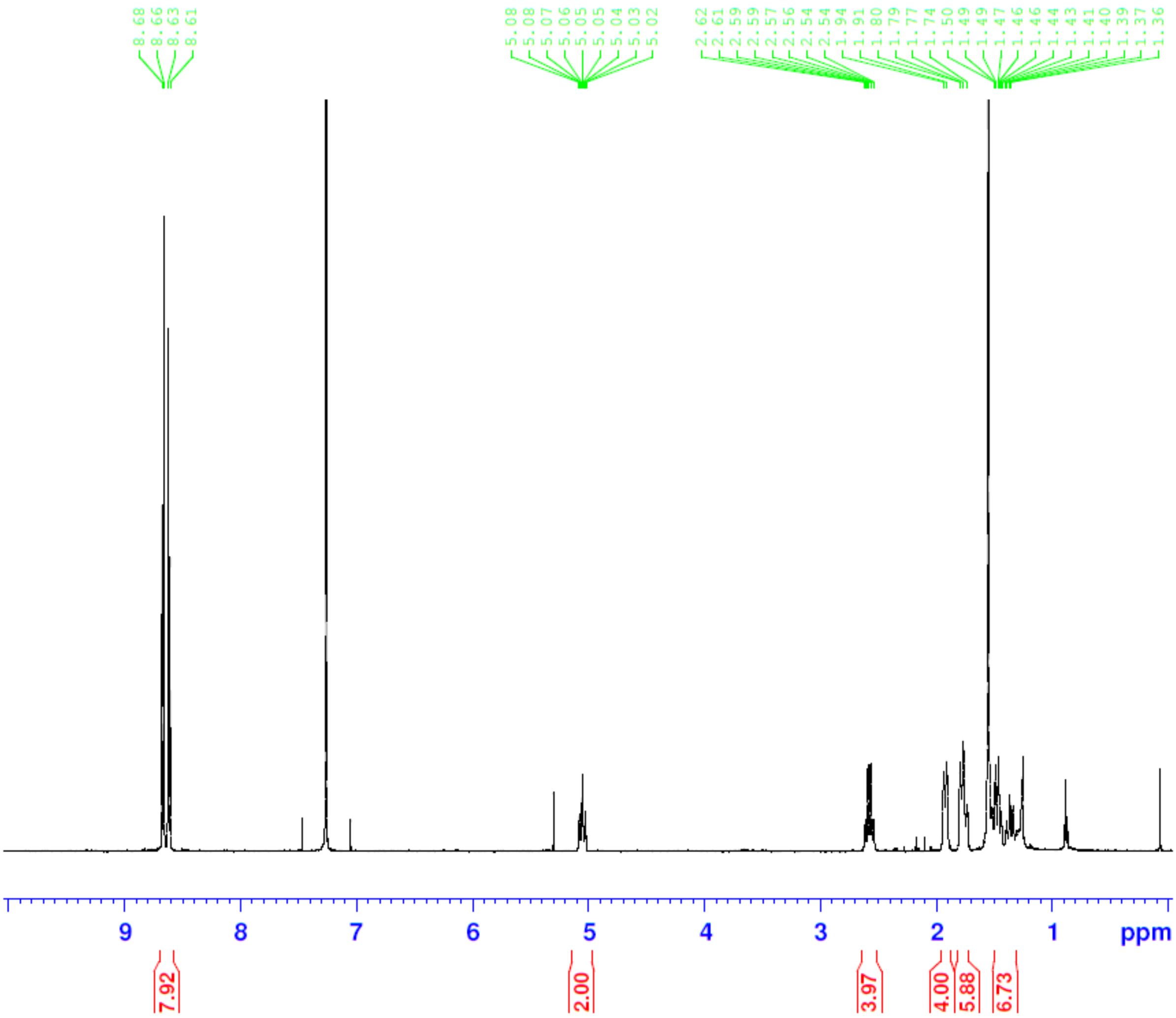

Supplementary Figure 6: ^{1}H-NMR of *N*,*N′*-dicyclohexyl -3,4,9,10-perylenedicarboximide

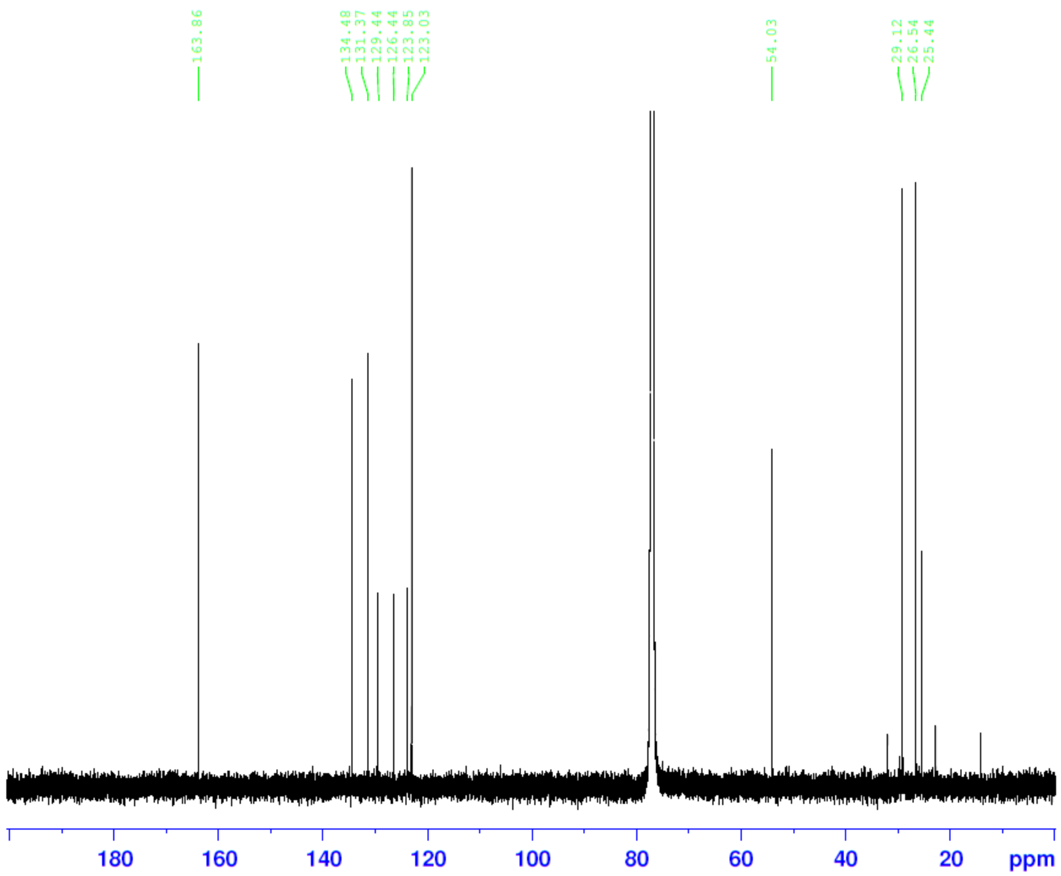

Supplementary Figure 7: ^{13}C-NMR of *N*,*N'*-dicyclohexyl -3,4,9,10-perylenedicarboximide

Nanobelts were fabricated by self-assembly of CH-PTCDI molecules using a phase transfer method as outlined previously by Che *et al.* [12]. A concentrated solution of CH-PTCDI (0.5 mL, 0.3 mM) was prepared in chloroform in a glass vial. This was then injected to the bottom of a solution of ethanol (~11:3 volume ratio, EtOH : PDI). The solution was allowed to sit in the dark at room temperature for 24 hrs and nanobelts were formed at the interface of the solvents. After mixing the two solvents, the nanobelts diffused into the whole solution phase and were transferred onto glass coverslips by pipetting (Supplementary Figure 8). The samples were then allowed to dry in a N_2 glove box before encapsulation and measurement. The solution was not shaken during the self-assembly process to prevent nanobelts with sharp ends forming.

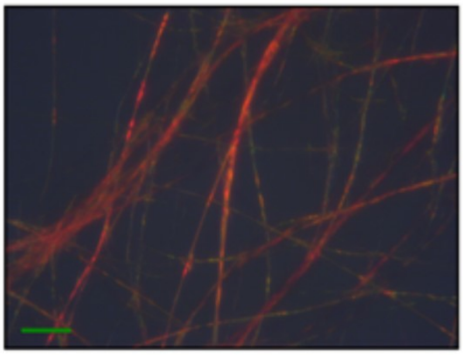

Supplementary Figure 8: Optical microscope image of PIC thin film in transmission. The wires are long and highly twisted. Scale bar is 10 µm.

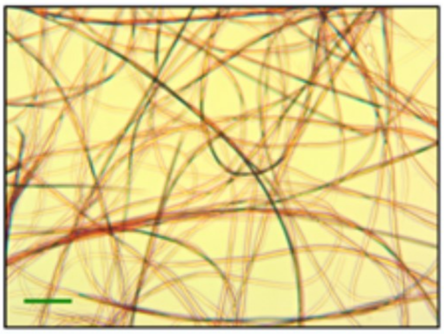

Supplementary Figure 9: Optical microscope image of PIC thin film in reflection. Scale bar is 10 µm.

Powder X-ray diffraction (PXRD) patterns (Supplementary Figure 10) were measured on a Bruker D8 Advance diffractometer equipped with a Cu K_α source (0.1 mm divergence slit, static) and a LynxEye XE detector. K_β radiation was attenuated with a 0.0125 mm Ni filter.

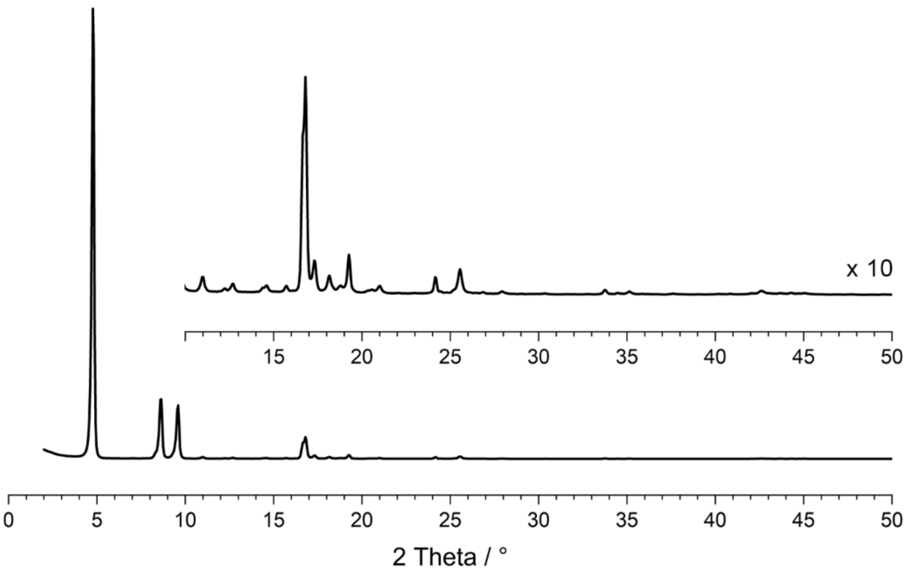

Supplementary Figure 10: Powder XRD pattern of the cyclohexyl PDI wires.

Supplementary Note 1: Sample characterization

To characterize the structure and morphology of the semiconductor thin films, a combination of techniques were employed, including; continuous rotation electron diffraction (cRED), transmission electron microscopy (TEM), scanning electron diffraction (SED), scanning electron microscopy (SEM), atomic force microscopy (AFM), and kelvin probe force microscopy (KPFM).

Cyclohexyl PDI structure determination by continuous rotation electron diffraction

cRED data were acquired from PDI nanobelts deposited on standard holey carbon coated 200 mesh Cu TEM grids (EM Resolutions Ltd.) using a JEOL JEM-2100 TEM operated at an accelerating voltage of 200 kV. The specimen was cooled to a temperature of ~100 K during data collection and during acquisition the goniometer was rotated continuously with a speed of 0.45 °/s. The PDI nanobelts were typically single crystals and selected electron diffraction patterns, acquired near to crystallographic zone axes, during this tilt series, are shown in Supplementary Figure 11.

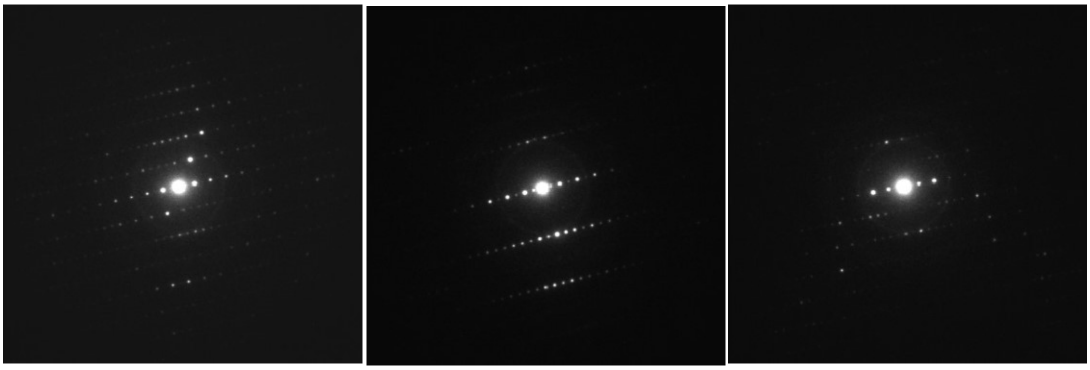

Supplementary Figure 11: Selected electron diffraction patterns from a continuous Rotation Electron Diffraction data set. The selected patterns are from orientations close to crystallographic zone axes.

The cRED datasets were processed using X-ray Detector Software (XDS) [13] where the observed reflections were indexed using a single unit cell and the corresponding intensities were extracted for structure solution and refinement. The structure was solved using the software SHELXT [14] and a least-squares refinement was performed in SHELXL-97 [15], converging with an R1 of 2879%, which is a typical value for structures solved using electron diffraction data and refined in a kinematical framework [16], details of the data and least-square refinement can be found in Supplementary Table 1. The determined structure was orthorhombic ($Pn2_1a$) with unit cell parameters (a = 22.9 Å, b = 37.6 Å, c =

7.2 Å, 90°, 90°, 90°). The cyclohexyl PDI molecules exhibited a herringbone packing with π-stacking near to the crystallographic c-axis as shown in Supplementary Figure 12.

**Crystal system**	Orthorhombic
**Space group**	$Pn2_1a$ (No. 33)
**a, Å**	22.9
**b, Å**	37.6
**c, Å**	7.2
**α, º**	90
**β, º**	90
**γ, º**	90
**Volume, Å^3**	6199.5
**λ, Å**	0.0251
**Exposure time per frame (s)**	0.6
**Tilt range, º**	-59.71 – +77.85
**Resolution, Å**	0.97
**Completeness, %**	79.9
**R_{int}**	0.159
**R1**	0.288
**No. of symmetry independent reflections**	6043
**Number of parameters**	256
**Number of restraints**	7

Supplementary Table T1: Details of the electron diffraction data and refinement

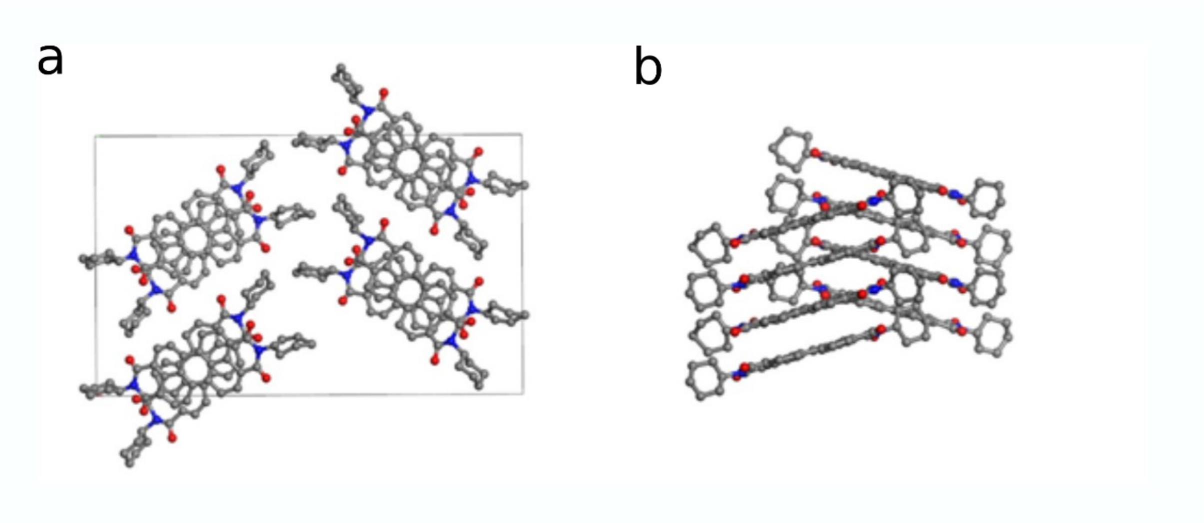

Supplementary Figure 12: The crystal structure of as determined from continuous Rotation Electron Diffraction data. **a.** View along the crystallographic *c*-axis **b.** shows the π-stacking of the molecules along an axis near to the (vertical) crystallographic *c*-axis..

Cyclohexyl PDI crystallographic mapping by scanning electron diffraction

Scanning electron diffraction (SED) data was acquired from the PDI nanobelts using a JEOL GrandARM 300F (S)TEM operated at an accelerating voltage of 300 kV with a probe current of 1.8 pA and a convergence semi-angle of ~0.6 mrad leading to a diffraction limited probe diameter of ~5 nm. Electron diffraction patterns were recorded using a Merlin/Medipix detector with 256×256 pixels at a camera length of 20 cm giving a calibrated pixel size of 0.010 Å^{-1}/px. Scans were performed with a calibrated step size of 7.73 nm/px over 256×256 probe positions. Virtual dark-field (VDF) images were formed by plotting the summed intensity in the diffraction plane, in the scattering vector magnitude range 0.75 to 1 Å^{-1}, as a function of probe position using the pyXem python library, as shown in Supplementary Figure 12. These images reveal the nanobelts to have a width of ~100 nm. Diffraction patterns from selected regions along the nanobelts illustrate that the nanobelts are approximately single crystals with a common orientation axis aligned along the long axis of the nanobelts, as shown in Supplementary Figure 13.

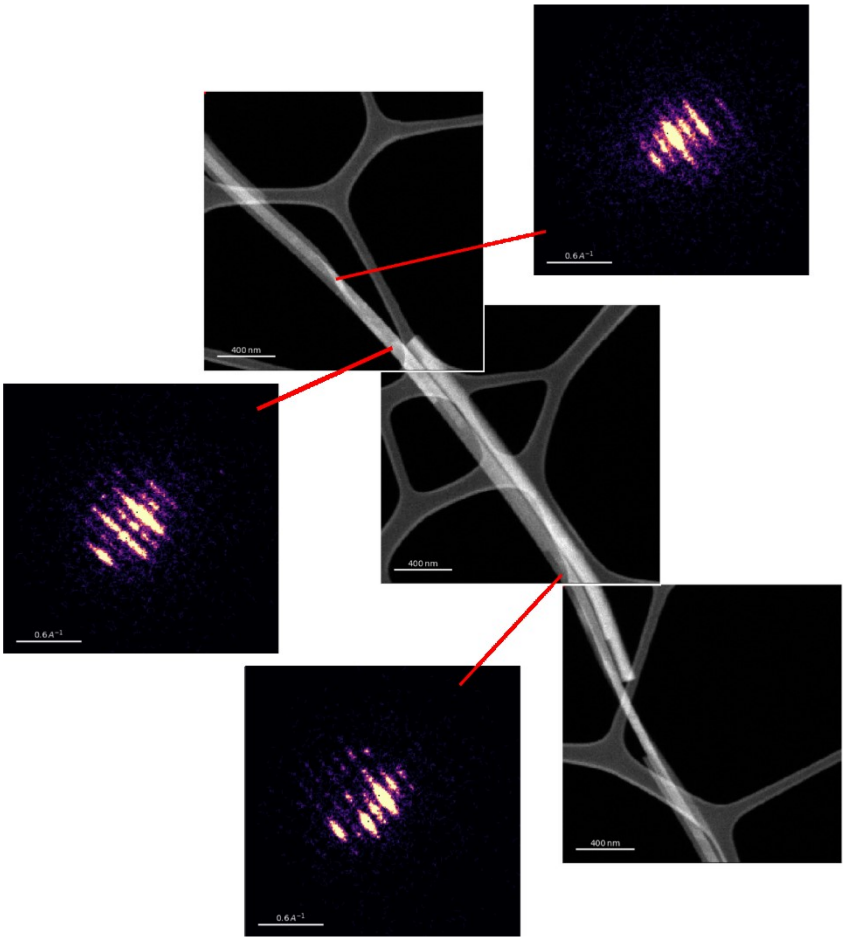

Supplementary Figure 13: Virtual diffraction images reconstructed from the SED microscopy data, integrating the scattering range 0.75 to 1 Å^{-1}, and representative electron diffraction patterns from selected regions along the nanobelts illustrating an approximately common crystal orientation.

Scanning electron microscopy of PDA and PIC thin films

To better characterise sample morphology, scanning electron microscopy (SEM) was performed on PDA and PIC thin films. In PDA (Supplementary Figure 14) it can be clearly seen that the crystal grows into 100 μm^2 domains with well-defined grain boundaries between the domains. Optical microscopy reveals the polymer to be highly aligned within a domain. Unpolymerized/unaligned regions of the sample (Supplementary Figure 14c) appear as small <1 μm^2 sized islands on the glass substrate.

SEM of PIC thin films (Supplementary Figure 15) shows the sample to be highly textured. In particular, there are large (>5 μm) grains in which it is presumed the nanotubes are bound. The remainder of the film appears to consist of a smooth background potentially arising from isolated nanotubes embedded within the sugar matix or isolated dye molecules.

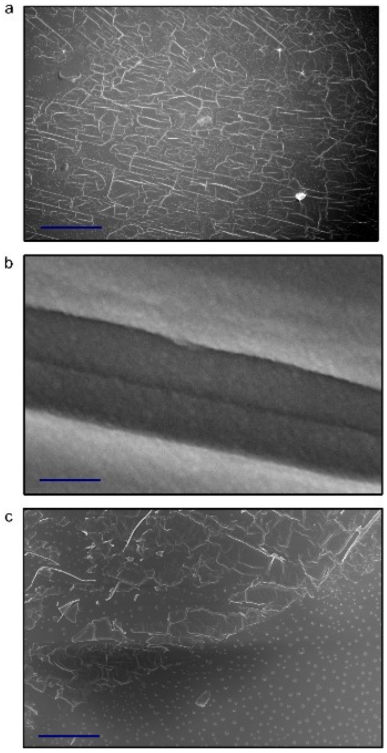

Supplementary Figure 14: a. Scanning electron microscopy (SEM) image of thin PDA crystal. The polymer chains are arranged into 100 μm^2 domains which themselves are highly aligned. The scale bar is 20 μm. **b.** Grain boundary between two PDA crystal domains. Scale bar is 500 nm. The domain boundary is relatively smooth. **c.** SEM image showing unaligned regions of PDA polymer. The domains are broken into small clusters and also show regions of incomplete spherulitic crystal growth. Scale bar is 20 μm.

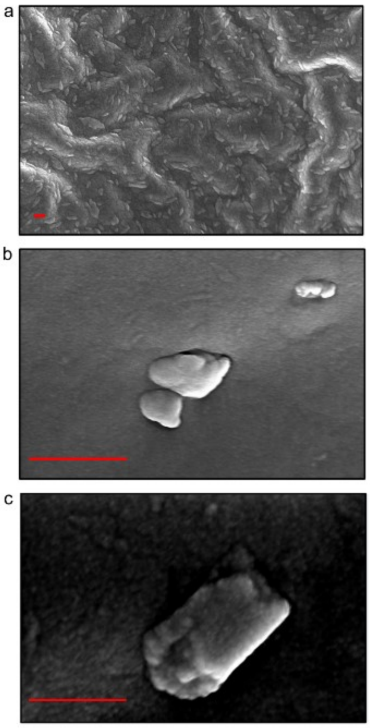

Supplementary Figure 15: a. Scanning electron microscopy (SEM) image of a PIC thin film. The clusters correspond to large bundles of nanotubes, with the background composed of the sugar matrix. The films charges easily even when sputtered in gold (50 nm) leading to some bright spots on the image. Scale bar is 5 µm in all the images. **b-c.** Zoom into SEM of cluster of nanotubes, which show a very rough surface structure. The background shows less aggregated nanotubes embedded into the sugar matrix.

Atomic force microscopy of PDI nanobelts

Atomic force microscopy was performed with a Veeco Dimension™ 3100 on individual PDI wires at room temperature (Supplementary Figure 16). The width of a wire was found to be ~130 nm and is uniform across the length of the wire shown in the image (>5 um). The surface of the wires is smooth with a root mean square (RMS) roughness of less than 1 nm, as illustrated in the height profile. This suggests highly-ordered packing of individual monomers. Consequently, the aggregates we suggest

aggregate to be low in structural disorder.

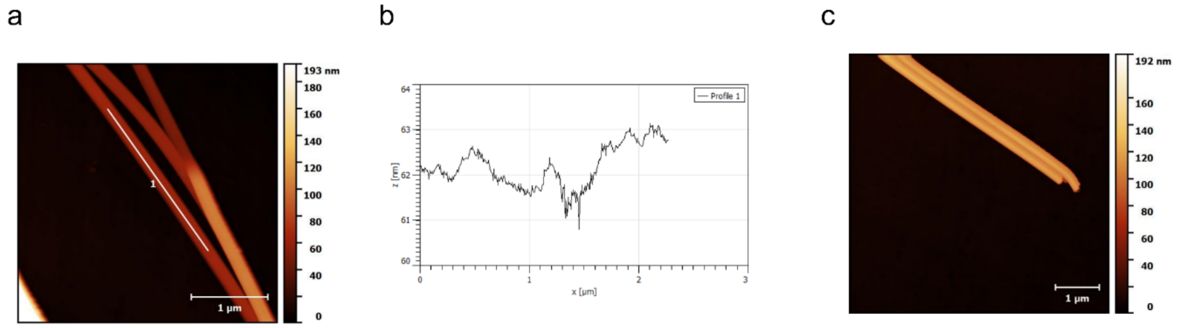

Supplementary Figure 16: a. AFM of PDI nanobelts showing the uniform topology of the surface. **b.** Topology profile taken along white line in a, the variation is height is less than 1 nm. **c.** The nanobelts show blunt ends. In cases where the ratio of ethanol to PDI monomer is incorrect, the wire ends can be sharp. Such wires were not considered in this work.

Transmission electron microscopy of PIC

To further characterize the PIC films, transmission electron microscopy was performed. Supplementary Figure 17 shows the J-aggregates consist of highly twisted and knotted tubes tens of nanometres thick and hundreds of nanometres long; bundles of such tubes are presumed to make up the clusters found in SEM. The nanowires are highly unstable even to low dose electron beams, hence high resolution images could not be collected.

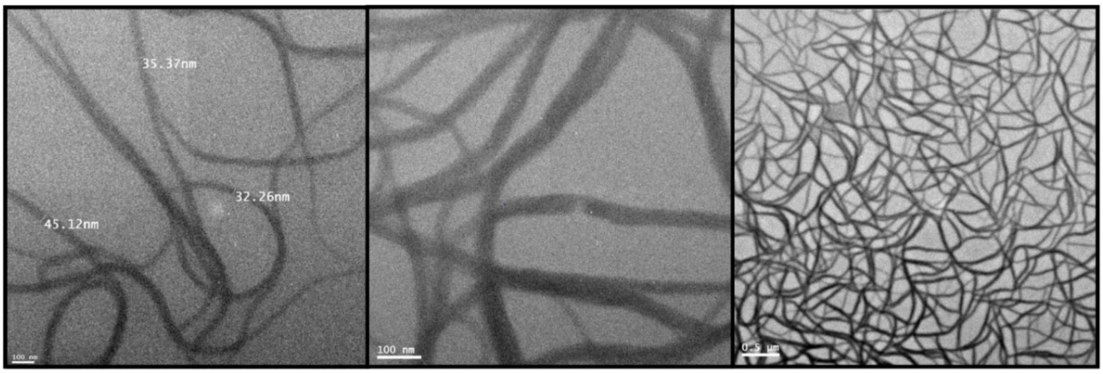

Supplementary Figure 17: PIC nanotubes embedded in a sugar matrix twist into threads tens of nanometres thick (left). Zooming into the left image (centre) shows each set of threads appear to consist of several nanotubes. Each of the threads is bundled into large agglomerations which are presumed to make up the clusters observed in SEM.

Kelvin probe force microscopy

Kelvin probe force microscopy was performed on eight different wires deposited on ~100 nm gold-coated glass slides. Separate locations were selected where wires were sufficiently separate from any aggregation of wire. The variation of work function along any isolated wires was less than 50 meV, suggesting low disorder.

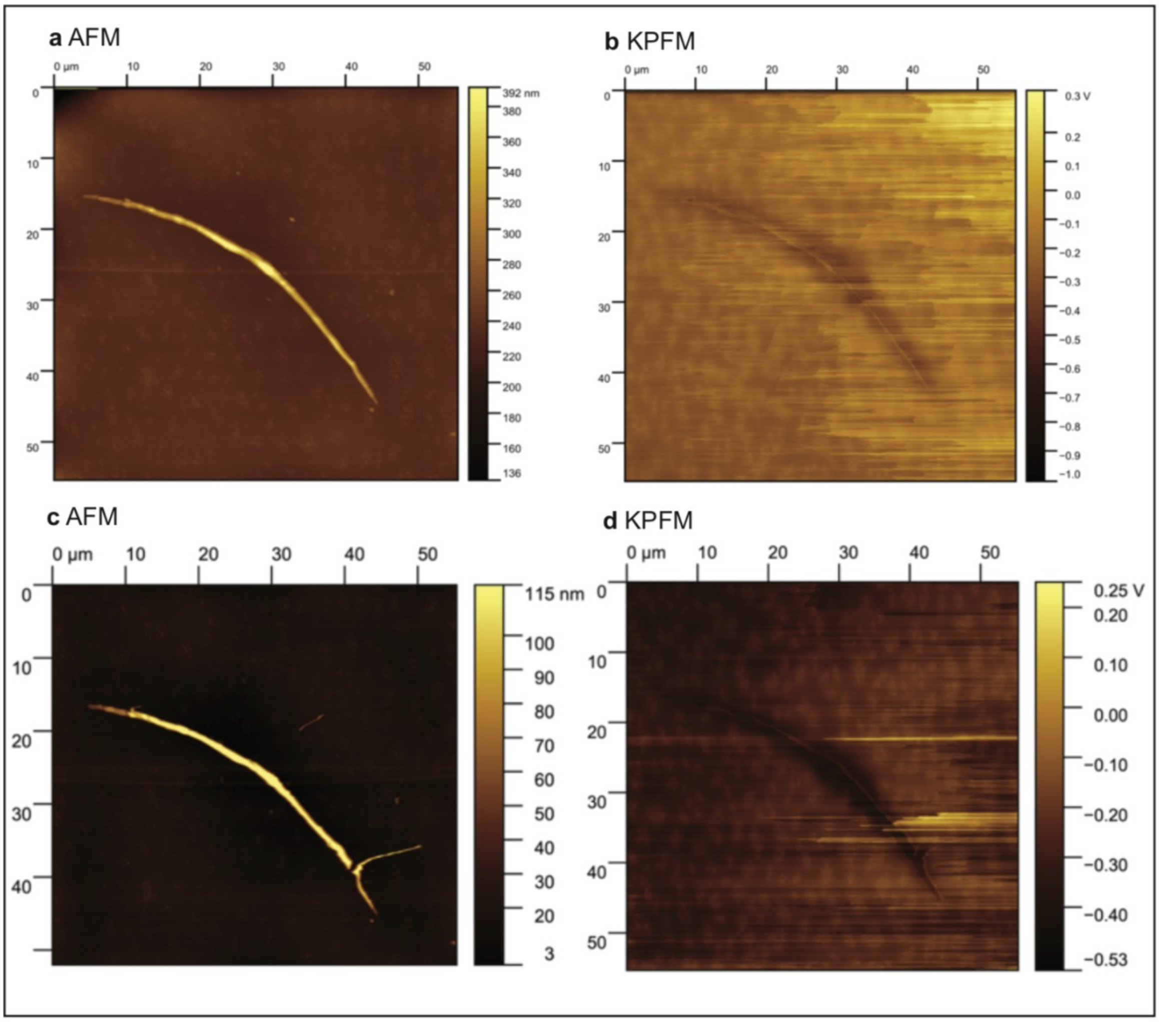

Supplementary Figure 18: Selected Amplitude Modulation mode Kelvin probe force microscopy (KPFM) and Atomic Force Microscopy (AFM) topology measurements taken on an Asylum Instruments KPFM-AFM using a n-type silicon tip with a platinum coating from Windsor Scientific (HQ:NSC35/PT). i) and ii) refer to the same wire, whereas iii) and iv) refer to a different wire where the end has been broken by indentation.

Confocal Microscopy

Confocal microscopy although cannot directly probe the microstructure of films is a useful tool in characterizing their microstructure. This is discussed below for PIC and PDI, ‘blue’ PDA is non-emissive and hence such measurements could not be used.

Photoluminescence microscopy of PDI nanobelts

Supplementary Figure 19a shows a confocal photoluminescence (PL) microscopy image of a PDI nanobelt. The PL intensity is relatively uniform across the nanobelt in keeping with the suggestion of the samples being low in structural defects. Measuring the spectrally resolved PL decay of nanobelts (405 nm excitation; decay captured with Andor iSTAR ICDD) shows a decay that is although asymmetric in shape, decays uniformly (23 ± 2 ns) across all wavelengths and is approximately independent of the position on the nanowire (white asterisk). This suggests there be to a single recombination pathway in nanobelts as opposed to localised defect emission.

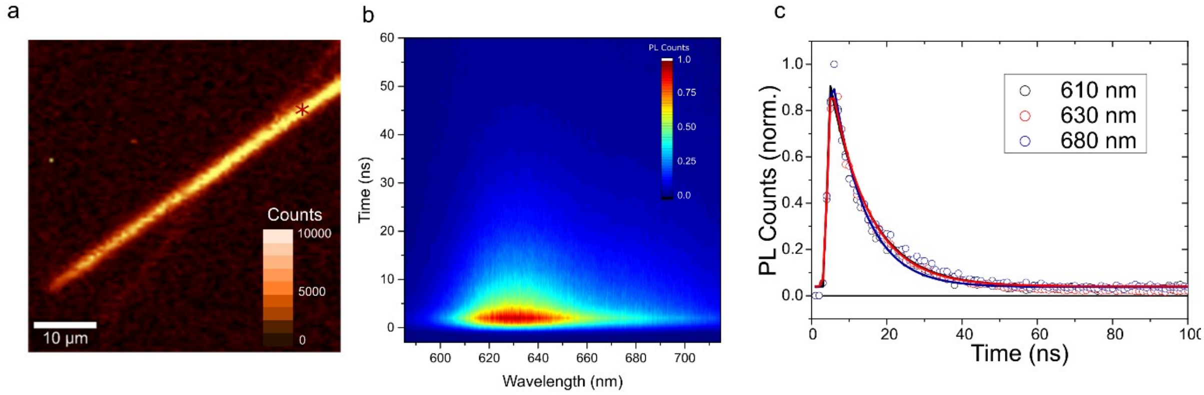

Supplementary Figure 19: a. Confocal photoluminescence (PL) image of two overlapped PDI wires, the bottom wire is in a different focal plane and hence appears dimmer. The PL is relatively uniform across the wire in line with a structure low in defect concentration. **b.** Spectrally resolved PL decay from PDI wire at location marked with white asterisk. **c.** The rate of decay is spectrally uniform with the normalised decay kinetics at 610 nm, 630 nm and 680 nm being nearly identical.

Photoluminescence microscopy of PIC thin films

Thin films of PIC are inhomogeneous. This is most readily demonstrated by confocal photoluminescence (PL) microscopy (405 nm excitation; ~500 nm FWHM spot size) as shown in Supplementary Figure 20 for a typical 10 × 10 μm region of the films. The film appears to contain regions where the nanotubes are clustered together leading to bright emission, as well as regions which appear to contain little to no nanotubes within the matrix. Unfortunately, experimental limitations

preclude correlation of the TAM images with confocal PL. However, we speculate that the highly bundled regions give rise to the largest expansion.

Cryogenic absorption spectra of PIC

Cryogenic absorption spectroscopy is a commonly used technique to estimate energetic disorder in molecular materials. We measured the absorbance of our PIC films at a range of temperature from 298 K down to 17 K. Supplementary Figure 20a shows the normalized absorbance as a function of temperature. The sharp J-band is clearly observed at 585 nm at room temperature and blue shifts to 580 nm when cooled to 17 K, accompanied by a small degree of narrowing. After a quadratic background removal, we fitted the J-band to a pseudo-Voigt profile (i.e. a linear combination of a Gaussian and a Lorentzian line shape):

$$y = A_{Gauss}\frac{e^{-(\hbar\omega-E_0)^2/2\sigma^2}}{\sigma\sqrt{2\pi}} + A_{Lorentz}\frac{\gamma}{\pi[(\hbar\omega-E_0)^2+\gamma^2]} \quad (1)$$

where σ and 2γ are the Gaussian standard deviation and Lorentzian FWHM respectively. The fitting results are shown in Supplementary Figure 20b-d We note that the absorbance spectra are asymmetrical, implying it is impossible to achieve a perfect fit to the data using a simple pseudo-Voigt line shape. However, it provides a reasonable approximation throughout the investigated temperature range, with a stronger contribution from the homogeneous Lorentzian line shape. The Lorentzian linewidth, γ, is found to decrease from 15 meV (~120 cm^{-1}) to 10 meV (~80 cm^{-1}) when cooled to 17K, which is notably smaller but on the same order of magnitude as many self-assembled J-aggregates (e.g. 50 meV in TDBC J-aggregates). This low disorder is likely to reduce exciton scattering and aid long-range polaritonic transport. Fitting γ to a power law reveals its quadratic dependence in temperature as shown in Supplementary Figure 20c, which theoretical models have shown relates to pure electronic dephasing [17] due to elastic two-phonon scattering of the excitons. The inhomogeneous broadening is likely the result of interactions with the inhomogeneous sugar matrix.

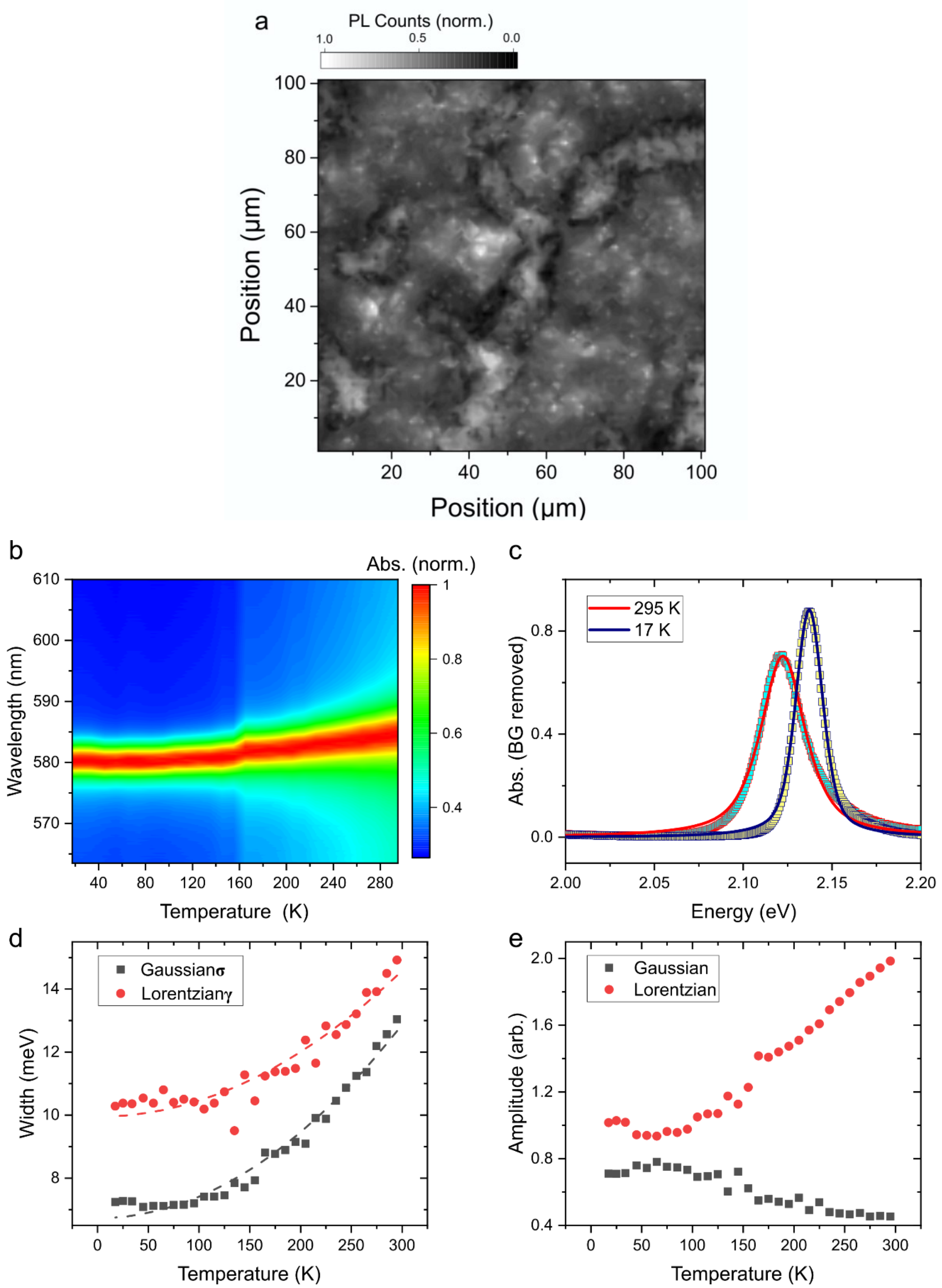

Supplementary Figure 20: a. Confocal photoluminescence (PL) map from a typical region of PIC thin film (normalized counts). The brighter regions are suggested to arise from tightly packed cluster of nanowires. They are also suggested to show the greatest level of polaritonic transport associated with Fresnel modes supported by the higher volume. **b.** Temperature dependent absorption response of PIC thin film. **c.** Background-subtracted absorbance at 17 K (blue) and 295 K (red) with corresponding fits

to a pseudo-Voigt function. **d.** Temperature dependent FWHM (extracted from Gaussian and Lorentzian peak modelling) fit to a quadratic relationship suggesting a 2-phonon pure dephasing mechanism for the observed broadening. **e.** Amplitudes (areas) of the fitted Gaussian and Lorentzian profiles. The Lorentzian line shape has a larger contribution across the whole temperature range. However, there is a considerable contribution from the Gaussian profile, suggesting inhomogeneous broadening mechanisms in our samples.

Supplementary Note 2: Specular reflection

Specular reflection measurements on PIC

Specular reflection measurements were performed on ~70 randomly selected locations of a PIC film. The reflection spectra obtained fall into two categories: those that do not show a splitting and those that do (Supplementary Figure 21). In the former case the spectra appear as approximately the inverse of the absorption with a sharp single excitonic dip at 583 nm (2.13 eV) and as well as a broad tail out to 550 nm, which we suggest to be associated with residual monomers. In the case of regions of the sample that do show a splitting, the J-band clearly shows two pronounced dips in reflectivity at ~579 nm and ~587 nm, whose relative intensities vary based on the precise sample location. In all cases, the separation between the peaks (splitting) is between 39 – 40 meV (Supplementary Figure 22).

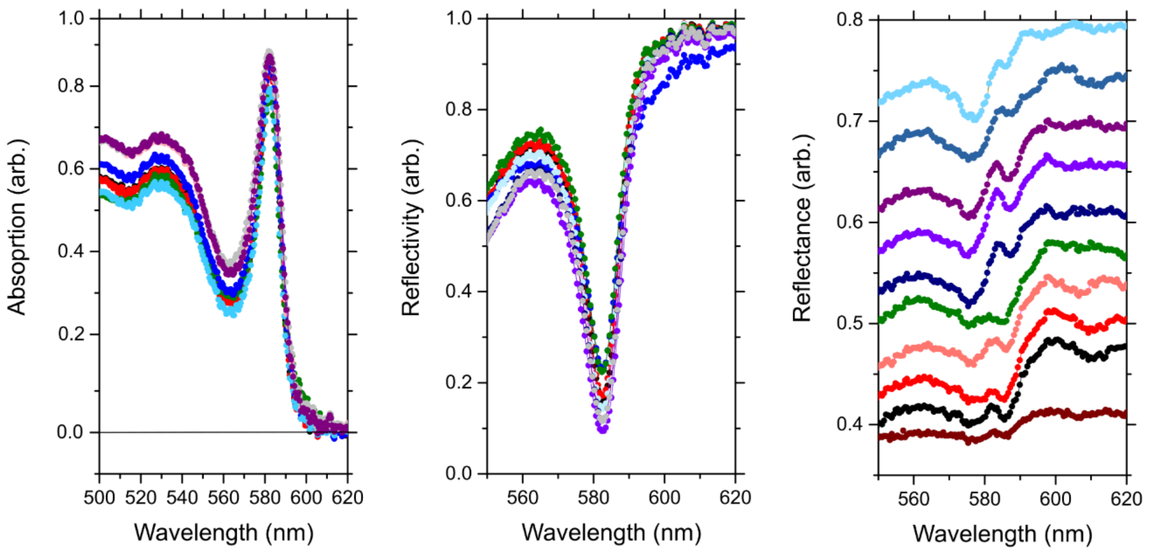

Supplementary Figure 21: a. Absorption spectra of PIC film in eight different locations. **b.** Reflection spectra in PIC film in regions that do not show a splitting in the excitonic peak. **c.** Reflectivity spectra of PIC film in regions of the sample showing a line splitting of ~ 40 meV. The off-set between spectra has been added to ease viewing.

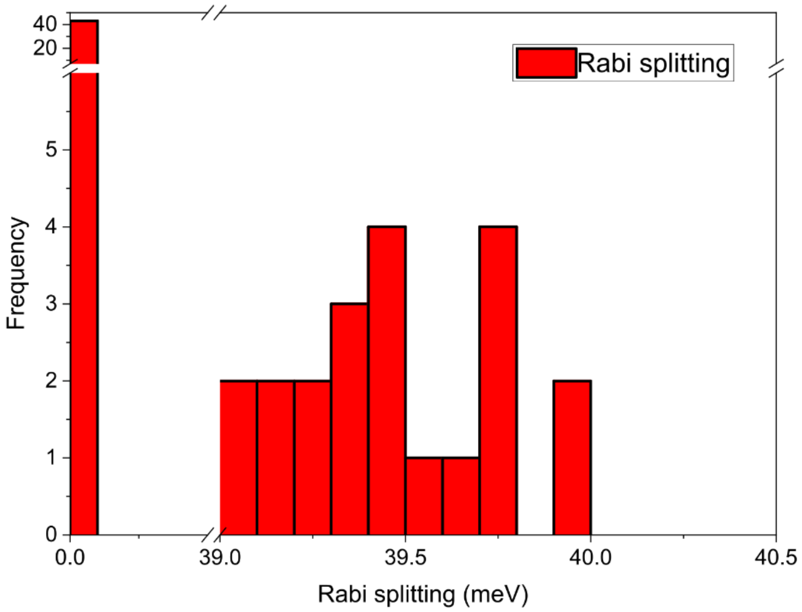

Supplementary Figure 22: Distribution of Rabi splittings measured in PIC thin films over ~70 spots. In a large number of locations (~40) no splitting is observed. In some places however, a splitting between 39 – 40 meV is observed.

Specular reflection measurements on PDA

Specular reflection measurements were performed on ~70 randomly selected locations of a PDA crystal. In all cases, the incoming light was polarized parallel to the long axis of the polymer chains. The reflection spectra obtained fall into two categories those that do not show a splitting and those that do (Supplementary Figure 23 and 24). In the former case the spectra appear as approximately the inverse of the absorption with a sharp zero-phonon and first vibronic peaks at 630 nm (1.97 eV) and 581 nm (2.13 eV) respectively. In the case of regions that do showing a splitting the spectral shape is similar, but the peaks associated with the zero-phonon line and first vibronic transition are clearly split. For the former the splitting is higher around ~18 meV, whereas in the latter case the splitting is slightly smaller at ~17 meV, in-line with the lower absorbance. The values are obtained from the peak to peak separation, but we do not stress their relative magnitude too strongly due the small difference which is also close to the noise level of our experiment.

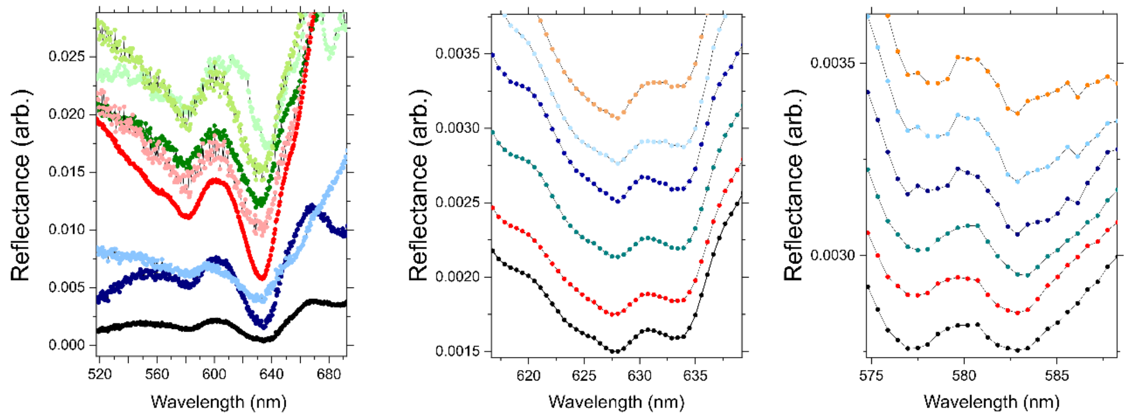

Supplementary Figure 23: a. Reflectance spectra from a region of PDA crystal that does not show a splitting. The curves are offset from one another for ease of viewing hence the absolute intensity is arbitrary. **b.** Splitting around the zero-phonon peak for several different sample locations in PDA, the lower polariton branch is typically more intense than that of the upper polariton. **c.** Splitting around first vibrational peak in PDA crystal. We note that in **b** and **c** the scale is also arbitrary with curves offset from one another.

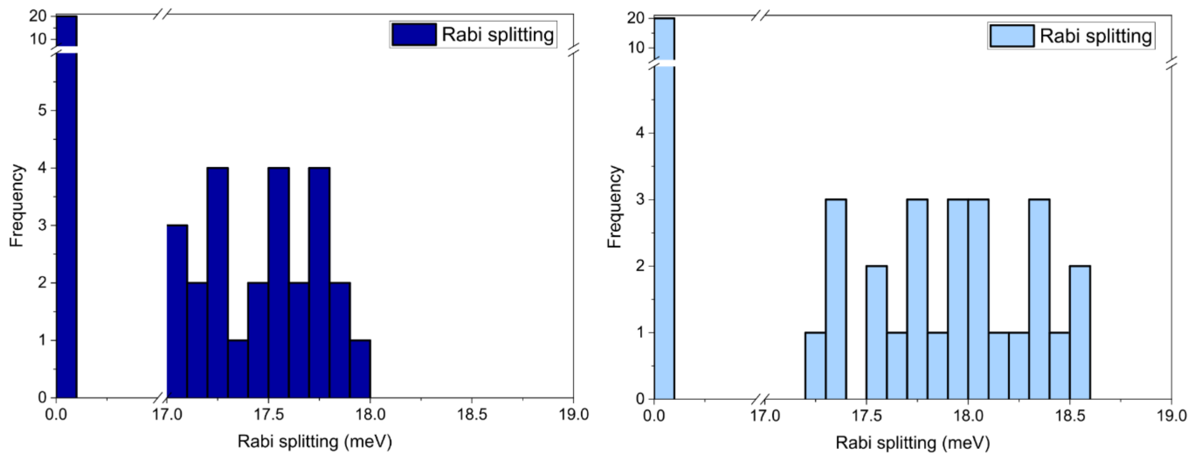

Supplementary Figure 24: Distribution of Rabi splittings measured in PDA crystalline films measured over ~70 spots. As for PIC a large number of sample locations do not show any splitting. However, in some cases the zero-phonon and first vibronic peaks at 630 nm (1.965 eV) and 581 nm (2.134 eV) are split. The splitting around the zero phonon peak (17.2 – 18.5 meV) is empirically larger than at the first vibronic transition where it is 17 -18 meV.

Absorption and Reflection spectra of PDI

Supplementary Figure 25 shows the absorption spectrum of a dilute solution of the PDI monomer, an evaporated film and the PDI nanobelts. The absorption spectrum blue shifts to higher energies between the solution and evaporated films or nanobelts, with a broadening of the vibronic peaks. Such behaviour is characteristic of the formation of an H-aggregate.

Microscopic specular reflectance measurements were additionally performed on PDI nanobelts. The reflectance spectrum is highly sensitive to polarization as can be seen from the red and black curves in Supplementary Figure 25b where the incoming light is parallel and perpendicular to the nanowire direction. The multitude of inhomogeneously broadened vibronic peaks precludes the observation of any Rabi splitting as in PDA or PIC. Under unpolarised light the reflectance spectrum (Supplementary Figure 25c) appears approximately as the inverse of the absorption.

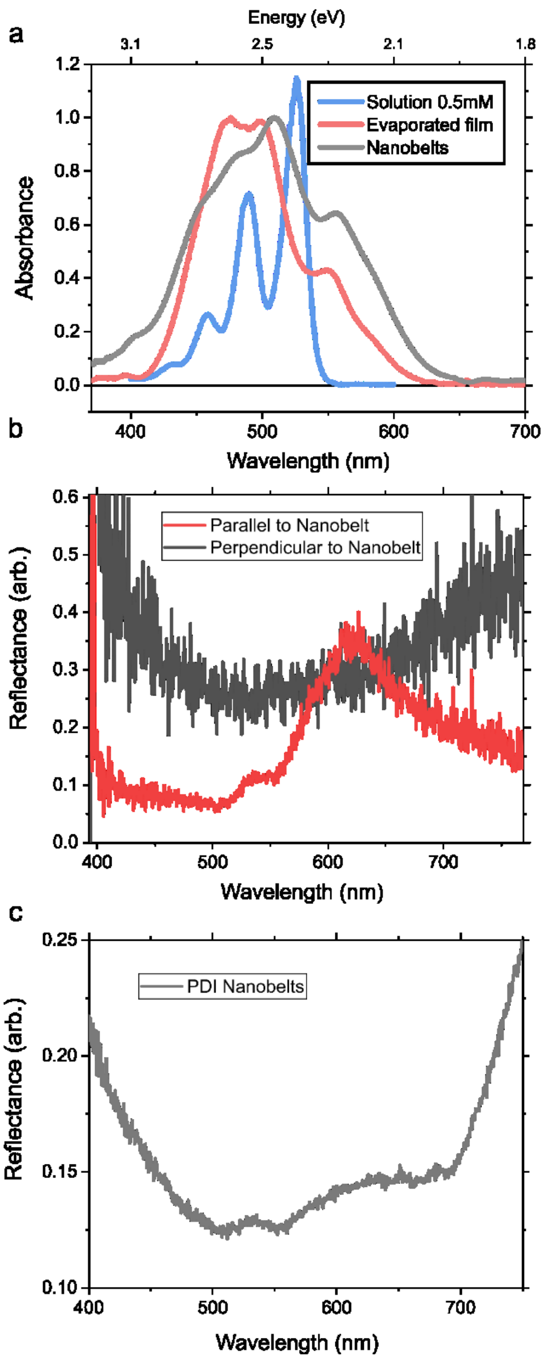

Supplementary Figure 25: a. Absorption spectrum of PDI monomer molecules diluted in chloroform (blue spectrum; 0.5 mM; 1 mm path length cuvette Hellma), a clear progression of vibronic peaks can be observed. The red and black curves show the absorption spectrum of an evaporated thin film of PDI and PDI nanobelts. The red shift is indicative of H-aggregation. **b.** Reflection spectrum of PDI nanobelts with light polarized parallel (red) and perpendicular to the long axis of the nanobelts. **c.** Reflectance spectrum of PDI nanowires with unpolarised light, the broad featureless spectrum precludes the observation of any Rabi splitting.

Supplementary Note 3: Transfer Matrix Simulations

The optical properties of thin films of PIC and PDA were explored in detail using transfer matrix simulations as previously reported [18], parameterised from the measured absorption of the organic films. The absorption spectra were fitted to a series of Lorentzian peaks, with the oscillator strength of each individually tuned to correctly reproduce the extinction of the measured film. Because the main absorption band in these materials is particularly strong and narrow, we found that we could satisfactorily describe the optical properties with consideration of only that band. We simulated a uniform slab of this absorber, on top of a 200 nm layer of SiO_2 and covered by 500 nm of air, to approximate the experimental situation (using tabulated values for the SiO_2 index of refraction, 1.0008 for air). The optical properties of the entire system (transmission, absorption, reflectivity) were calculated as a function of angle. Our aim was to determine whether and in what regimes these absorbers could reproduce the splitting in reflectivity noted in main-text Figure 1d-e, for which purpose we focus only on the modelled normal-incidence reflectivity. On the length scales relevant to our experiments, it is clear that the PIC and PDA films are highly inhomogeneous (see e.g. Supplementary Figure 20). The bulk thin-film absorption measurement reflects the contribution of highly concentrated bundles of active material, within a much more weakly absorbing matrix. In order to describe the effects of this, we have incorporated linear scaling factor to the oscillator strength in our model (effectively equivalent to scaling the concentration). Setting the scaling factor to zero (Supplementary Figure 26a below) allows us to directly visualise the Fresnel modes of the material slab in the absence of any light-matter interactions.

As summarised in Supplementary Figure 26, we find that when PIC is treated as uniformly distributed (scaling factor = 1) or weakly bundled (scaling factor = 2) there is no reflectivity splitting at the main absorption peak (dashed line) for any slab thickness. However, on increasing the bundling (scaling factor = 5) we find a slight splitting for slabs of 2.3 and 2.5 µm. Further increasing the scaling factor results in the splitting becoming evident for still thinner slabs. The onset of the splitting is a product of both 'concentration' (scaling factor) and thickness. For a 1.7 µm film (Supplementary Figure 26g) we require a 15x local variation in absorption to account for our observed splitting, while at 2.3 µm thickness (Supplementary Figure 26h) even a 5× factor results in detectable splitting. To confirm the origin of this splitting as a polaritonic (i.e. light-matter coupling) effect, we have extracted the separation between peaks for a 2.3 µm film (arrows in panel h) as a function of scaling factor. Supplementary Figure 26i shows that this splitting closely follows the square root of the scaling factor, equivalent to the sqrt(N) dependence typical of the light-matter coupling regime. We thus consider these peaks to be a signature of bulk polaritons[19–21], formed as a consequence of the extremely high oscillator strength in these aggregates.

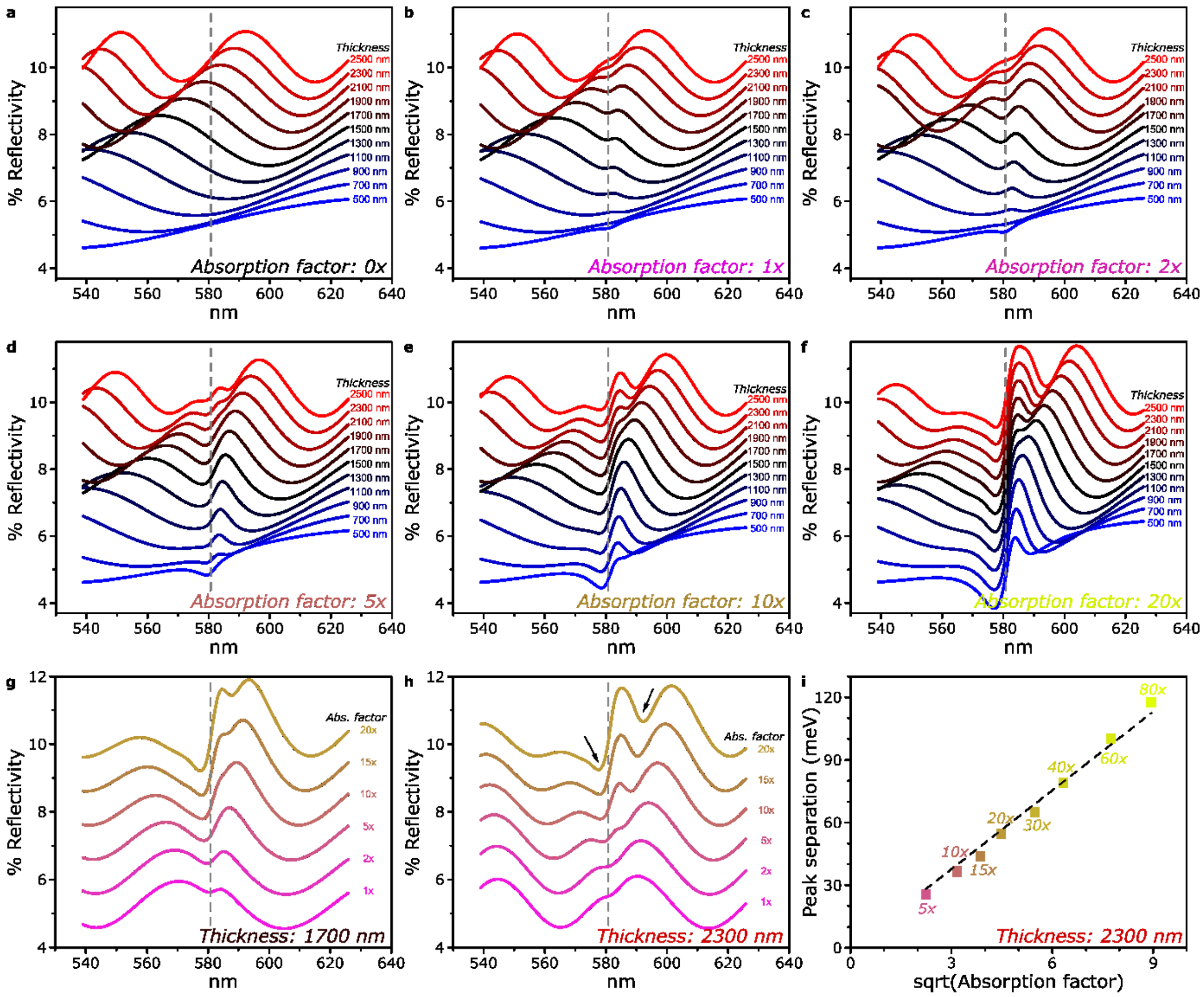

Supplementary Figure 26: Transfer-matrix simulations of normal-incidence reflectivity for PIC film. **a** Thickness-dependent Fresnel modes are evident when the dye oscillator strength is set to zero. **b,c** Weak absorption/bundling results in a slight dip at the exciton energy (dashed line), but only at a single transition. **d,e,f** At still higher oscillator strength, for sufficiently thick films, the dip in reflectivity splits into two peaks on either side of the exciton energy, a sign of exciton-polariton formation. **g,h** . Reflectivity at fixed film thickness as a function of scaling factor, showing the onset of splitting is both thickness- and absorption-dependent. **i.** Degree of peak separation, plotted versus the square root of the absorption scaling factor (equivalent to concentration). The linearity (dashed line) confirms that the origin of these peaks is polaritonic, i.e. light-matter interactions.

Our simulations do not depend on the particular chemical identity of the material, and we thus expect the result from PIC to be generalizable: for a given transition linewidth, there should be some region in the thickness/scaling factor space where a splitting is observable (though whether that regime is practically achievable depends very much on material). Thus we have obtained comparable splitting using our model for the strongest absorption peak of PDA. However, to reproduce the small splitting suggested in the main text, we had to consider a region of reduced oscillator strength and narrowed transition linewidth (Supplementary Figure 27), suggesting that our measurement might report on a subset of particularly well-ordered regions within the bulk.

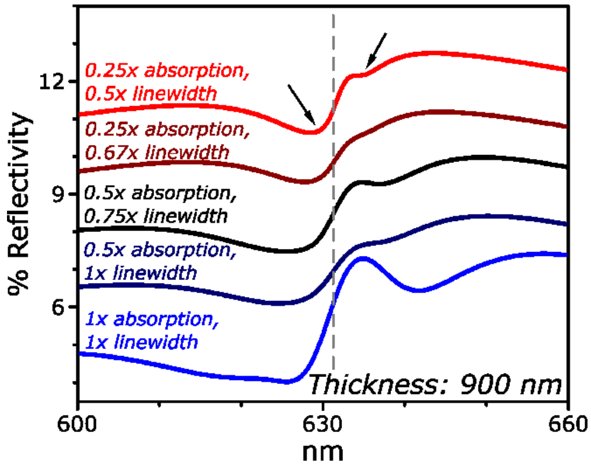

Supplementary Figure 27: Transfer-matrix simulations of normal-incidence reflectivity for PDA thin film. Only for a significant reduction in the oscillator strength and linewidth can we reproduce a narrow splitting (arrows) comparable to that tentatively assigned in the main text.

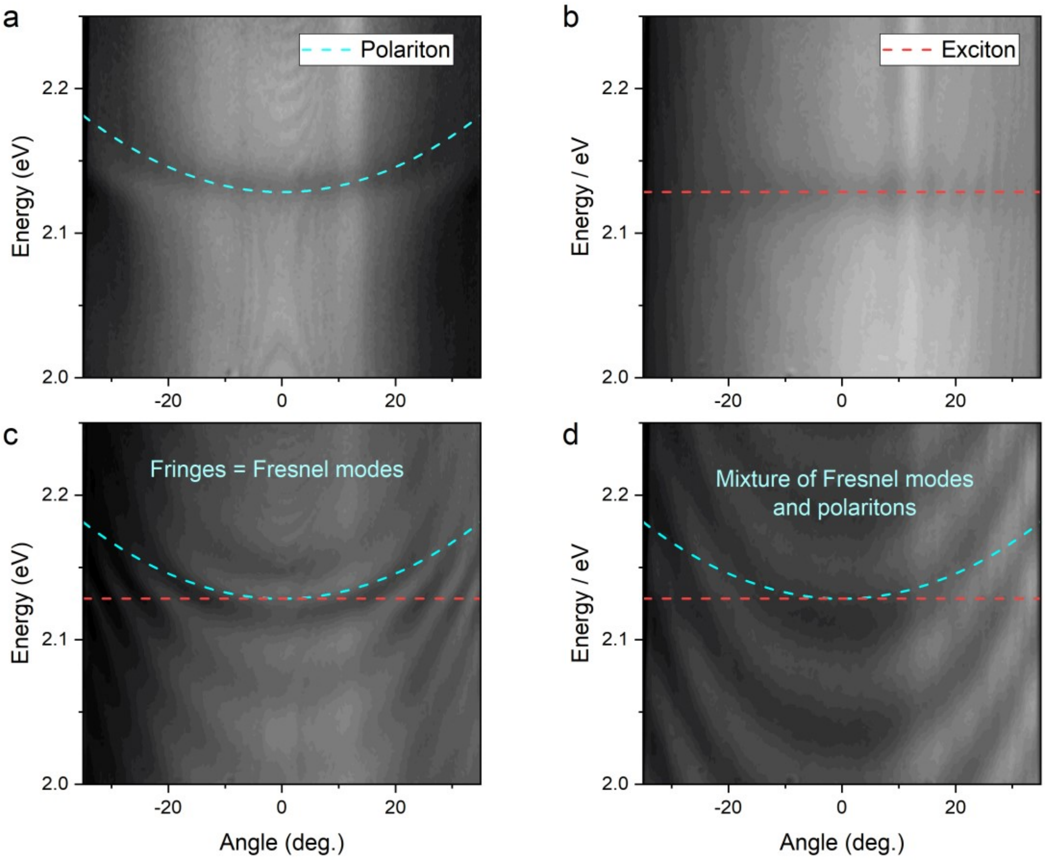

Supplementary Figure 28: Angle-dependent white-light reflectivity of PIC film, measured in k-space using a high-NA lens. a. In isolated areas of the film we primarily resolve a single clean dispersion which closely approaches the bare exciton energy (2.13 eV) at 0 degrees. Very faint signatures of underlying Fabry-Perot modes with steeper dispersion can be identified at high angles and/or low energies. **b** On other areas of the same sample where no optical modes can be supported, we detect the dispersionless signature of the exciton. **c** In other regions of the film we primarily detect a

ladder of intrinsic Fabry-Perot modes with relatively steep dispersion. These cross directly over the exciton absorption energy. **d.** Complex mixtures of these features can also be detected.

To probe the nature of the splitting of the exciton transition identified with normal-incidence reflectivity measurements, we investigated the angular dependence of the reflectivity. These measurements were performed on PIC films, where the splitting is most pronounced, using the k-space setup described in Methods. Spatial filtering was used to select areas ranging from 10-50 μm, and thus the signal is highly sensitive to the inhomogeneity of the film. Accordingly, we observe a range of different behaviours as we scan across the film surface. In some locations (Supplementary Figure 29a) we recover a single shallow dispersion, positioned at slightly higher energy than the exciton transition (panel b). This dispersion never crosses the energy of the exciton transition, and from the analysis below we can assign it to the upper polariton. In many locations, where the oscillator strength is too low to support polaritons, we instead detect a ladder of photonic modes. These have distinctly steeper dispersion than the band in panel a, confirming they have a different physical origin.

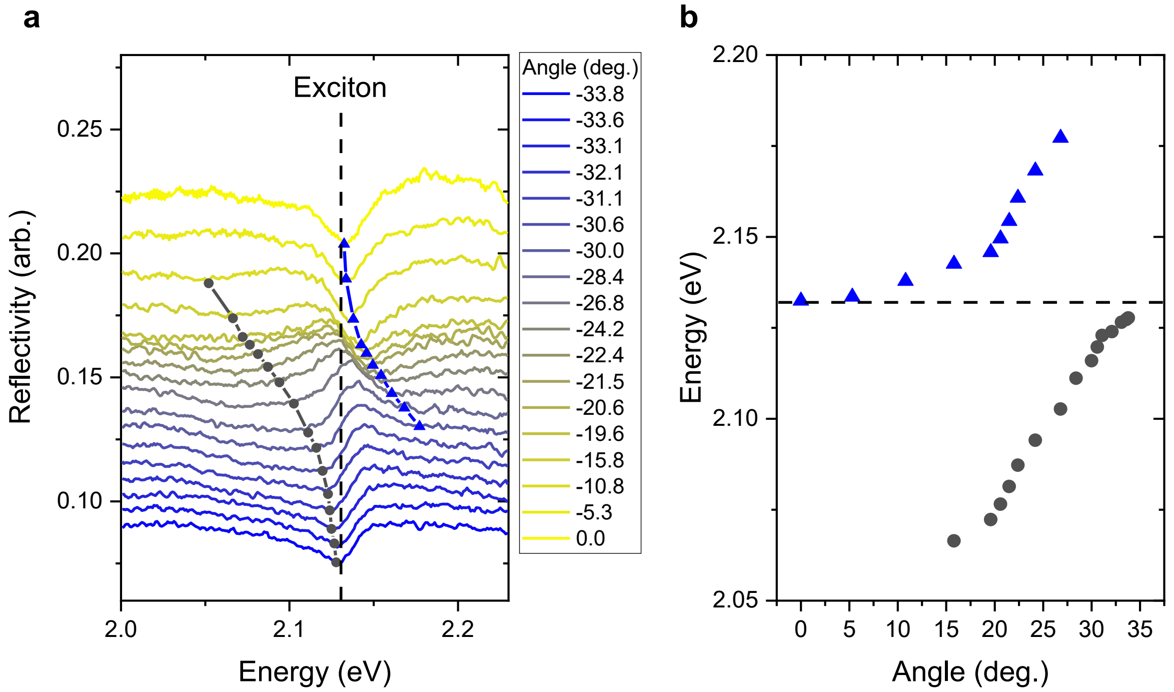

Supplementary Figure 29: Reflectivity as a function of photon energy at different incident angles is shown in panel a. Each curve has been displaced vertically for clarity. The solid black and blue lines are only guides to the eye to highlight the angle-dependent dips in reflectivity below and above the excitonic transition respectively. The energies of these dips are also plotted against their angles in panel **b**. resembling a typical anti-crossing of a cavity photon mode and an excitonic transition.

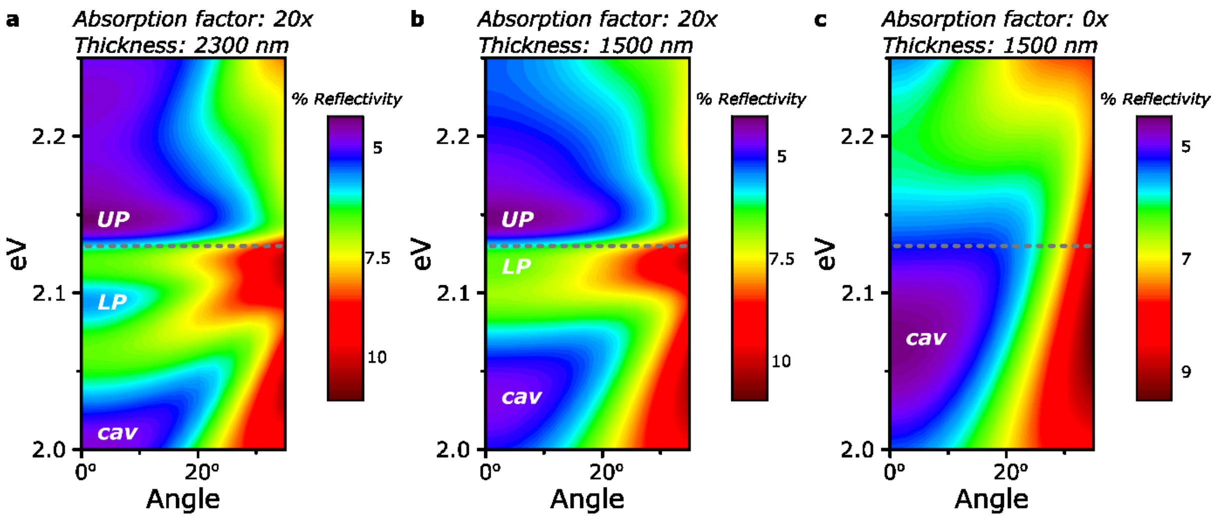

Supplementary Figure 30: Reflectivity dispersion for PIC films, calculated using transfer matrix model. Varying film thickness in **a** and **b** shifts the energetic positions of polariton (UP, LP) and photonic (cav) modes, as in normal-incidence calculations in Supplementary Figure 63. **c** When the PIC oscillator strength is set to zero, the steeper angular dispersion of the intrinsic Fabry-Perot modes can be discerned. Upper and lower polariton are labelled based on their identification in oscillator strength-dependent calculations in Supplementary Figure 63i. Energy of the primary PIC exciton transition is shown in grey dash.

In order to understand the appearance of these features we have performed further transfer matrix modelling (Supplementary Figure 30). We have used the same sample structure as in the normal-incidence reflectivity calculations above (Supplementary Figure 26). Here we focus on the regime of strong 'bundling' with an absorption scaling factor of 20, but the same conclusions apply to many other combinations of parameters. In panel **a** we show the angle-dependent reflectivity over the same range measured above, for a film of 2300 nm thickness where the upper and lower polariton peaks can be readily identified from normal-incidence calculations (Supplementary Figure 26h). We compare to calculations for a 1500 nm film, where the lower polariton can only be weakly discerned at normal incidence (Supplementary Figure 26f). In both structures the simulations show a low-energy photonic mode with relatively steep dispersion, labelled cav. By contrast, the upper polariton (UP) band exhibits very weak angle dependence. The lower polariton (LP) has a similarly shallow dispersion and additionally exhibits a much weaker modulation in overall reflectivity. Indeed, depending on the film thickness (i.e. detuning) the lower polariton can be almost impossible to detect despite the upper polariton branch remaining prominent. By comparison, if the oscillator strength of the material is set to zero (panel c) the simulation returns a single well-defined optical mode with steep dispersion. In light of these simulations, we find that the behaviour evident in Supplementary Figure 30a – a single prominent dispersion slightly above the exciton energy, with much slighter angle dependence than the Fabry-Perot modes – is most consistent with light-matter interactions, leading to the formation of distinct polariton states.

Supplementary Note 4: MEEP Calculations

The characteristic spectral signature of light-matter coupling between a photonic mode and a resonant emitter excitation is the presence of two peaks that are respectively redshifted and blueshifted from the interacting resonances. This distinctive peak splitting is observed in the reflectance spectra for nanostructures of PDA (Figure 1e) and PIC (Figure 1d) but not of PDI (Supplementary Figure 25b). However, the experiments probing long-range energy transport (Figure 3 of main text) suggest that light-matter coupling is present in all three materials; presumably, the polariton states cannot be spectrally resolved for PDI due to its progression of substantially damped vibronic transitions (Supplementary Figure 25a, blue curve). In this section, we report calculated reflectance and absorbance spectra for a model system resembling the PDI nanobelts. These simulations are carried out with the finite-difference time-domain (FDTD) method using the software package MEEP [22]. Assuming one absorptive resonance to represent the first few PDI vibronic transitions, as well as considering only the major geometric features of the nanobelts, our computational treatment shows that light-matter coupling is possible in the PDI nanobelts. Specifically, the results provide insight on the nature of the photonic modes and their coupling to PDI.

The model system consists of a single nanobelt, treated as a rectangular prism that extends infinitely and possesses translational invariance along the *z*-axis. In light of this size assumption, which is motivated by the fact that the actual nanobelt length is much larger than any dimension of the beam spot, we restrict our computational cell to the *xy*-plane, i.e., the plane containing the short-axes cross section (width × height = 50 nm × 800 nm) of the structure; the essential spectral features are expected to be preserved upon this dimensional reduction [23]. The nanobelt material is modelled with the orientationally isotropic Lorentzian dielectric function (e.g., see [24]; in this section, $\hbar$ = 1 hereafter)

$$\epsilon(\omega) = \epsilon_\infty + \frac{f\omega_0^2}{\omega_0^2 - \omega^2 - i\omega\gamma_0}, \quad (2)$$

where $\epsilon_\infty = n_\infty^2 = 1.62^2$ is the high-frequency dielectric constant, ω_0 = 2.422 eV (corresponding to wavelength of 512 nm) is the frequency of the molecular transition, γ_0 = 40 meV is the damping, and $f = \frac{2\mu^2\rho}{\epsilon_\infty\epsilon_0\omega_0}$ is the dimensionless oscillator strength. In calculating f, we actually use $\mu^2\rho$ = 60 D^2/nm^3 (see Supplementary Note 7), where μ is the magnitude of the transition dipole moment along either *x* or *z* and ρ is the number of molecules per unit volume. Furthermore, since our simulations only consider

light polarized along the nanobelt long-axis, we would obtain the same results if we use an anisotropic dielectric tensor polarized along z. All points below the nanobelt lie in borosilicate glass (n = 1.517), while the remainder of the surrounding medium lies in air (n = 1). The absorbing boundaries of the computational cell are of the perfectly matched layer (PML) type. For all calculations (i.e., near-field intensity distributions and optical spectra), we do the following once with all materials present (sample run) and once with only vacuum (background run): first propagate the fields produced by a Gaussian line source—which only has field component E_z—placed above the nanobelt until all fields have sufficiently decayed, and then Fourier transform the fields. Using the frequency-domain fields, the total flux passing through reflectance and absorbance power monitors is calculated. At each frequency, reflectance and absorbance are calculated by dividing the total flux through their corresponding power monitors for the sample run by the total flux through the reflectance power monitor for the background run. Lastly, the employed setup and fixed electric polarization of E_z cause the simulated fields to be symmetric with respect to the yz-plane; specifying this reflection symmetry in MEEP shrinks the simulation cell even more, leading to additional gains in computational efficiency. See Supplementary Figure 31 for other geometric parameters and a summary of the above simulation details.

To understand the nature of the electromagnetic modes coupled to PDI, we first analyze the reflectance spectrum (Supplementary Figure 25a) when the single molecular transition at 512 nm is not coupled to light, i.e., f = 0 and $\epsilon(\omega) = \epsilon_\infty$. There are four peaks, two redshifted and two blueshifted with respect to 512 nm. Upon calculating the near-field intensity distributions at each of the peak frequencies (Supplementary Figure 32), we find that the photonic eigenmodes correspond to standing waves with m = 2, 3, 4, and 5 antinodes confined along the nanobelt height. Observed in the simulated reflectance spectra for PIC film (Supplementary Figure 26) and also referred to as Fresnel modes (see Supplementary Note 9), these electromagnetic resonances are well-known in photolithography [25]: for a stack of three dielectric slabs, where the middle slab has a different refractive index than the other slabs, light traveling from first to third slab will produce standing waves in the middle slab. The standing waves arise from interference between the wave transmitted from the first slab to the second slab and the wave reflected from the third slab to the second slab.

As a control, we also calculate the absorbance spectrum (Supplementary Figure 32b) of the system when the oscillator strength of the molecular transition is low, namely at 1% of its full value. The single observed peak is just a Lorentzian curve centred at the molecular transition energy. This spectral profile is exactly what we expect for weak light-matter coupling. Indeed, the observed full width at half maximum (FWHM) is 44 meV, only a little more than the linewidth of 40 meV for the excitation without any coupling to light, i.e., zero oscillator strength.

We now determine how strongly the standing-wave electromagnetic modes interact with the effective PDI transition at 512 nm when the oscillator strength is set to its full value. The computed reflectance spectrum (Figure **SI26c)** displays four peaks that resemble those of the spectrum in the absence of light-matter coupling, as well as a peak around 512 nm with nontrivial lineshape. In particular, each of the former four peaks is either redshifted or blueshifted if the corresponding peak in the bare spectrum is higher or lower in energy, respectively, than the molecular transition. These observations suggest that each shifted peak represents a polariton state with mostly photonic character, formed from the off-resonant interaction between a standing-wave mode and a molecular excitation of the same spatial symmetry.

To verify this claim, we first confirm that the energy shifts are due to light-matter coupling. For each photonic eigenmode m corresponding to the peak with energy $\omega_{c,m}$ in the reflectance spectrum with no light-matter coupling (Supplementary Figure 32a), we fit the appropriate eigenvalue of

$$H_m = \begin{pmatrix} \omega_{c,m} & g_m\sqrt{N} \\ g_m\sqrt{N} & \omega_{e,m} \end{pmatrix} \quad (3)$$

to the energy of the corresponding shifted peak (Supplementary Figure 32c), with the light-matter coupling strength

$$g_m\sqrt{N} = \sqrt{\frac{\omega_{c,m}\rho}{2\epsilon_\infty\epsilon_0 V_m/V_{sample}}} \quad (4)$$

as the only fitting parameter. N is the number of coupled molecules, V_m/V_{sample} is the ratio of the volume of photonic mode m to the volume occupied by these molecules, and $\omega_{e,m} = \omega_0$ is the energy of the molecular transition interacting with electromagnetic resonance m. The other eigenvalues of the fitted Hamiltonians H_m are well-aligned with the peak and shoulders around 512 nm (Supplementary Figure 25c). Therefore, these spectral contributions are those of the matter states in the presence of the same off-resonant light-matter interaction.

We now discuss the achievement of light-matter coupling. Summarized in Supplementary Table 2, the fitted $g_m\sqrt{N}$ are on the order of 100 meV and the associated V_m/V_{sample} are order 1-10. The latter

values indicate moderate field confinement, a typical prerequisite for achieving light-matter coupling [26]. To more accurately assess if this interaction regime has been reached, we must also consider damping processes. This necessity rests on the following intuition [26]: hybrid light-matter states form when the constituent states decay slow enough to allow coherent energy exchange. Thus, we also determine an effective strength $\kappa_m/2$ of coupling between each electromagnetic resonance and decay-inducing bath modes (Supplementary Table 2). We evaluate $\kappa_m/2$ as the half width at half maximum (HWHM) of the reflectance peak for mode m in the absence of light-matter coupling (Supplementary Figure 32a). The HWHM for each peak is calculated using the side (left or right) that reaches lowest in signal. That is, we use the right, left, and left sides of the peaks at 442, 572, and 833 nm. Since neither side of the peak at 368 nm has points whose signal is less than the maximum signal, we do not determine $\kappa_m/2$ for m = 5. Comparing $g_m\sqrt{N}$ and $\kappa_m/2$, we conclude that photonic modes m = 3 and 4 couple to PDI almost as much, if not more, than they do to their respective damping degrees of freedom. Moreover, $g_m\sqrt{N}$ is much larger than the strength $\gamma_0/2$ at which PDI couples to its bath. Therefore, the light-matter subsystems for m = 3, 4 are near or at the limit of light-matter coupling. Further evidence that this interaction regime has been reached is the absorbance spectrum (Supplementary Figure 32d), which demonstrates splitting of the peak from the spectrum with relatively weak light-matter coupling (Supplementary Figure 32b) into a peak and shoulder that are shifted in energy from the original molecular transition. As an aside, note that there is a lack of a definitive Rabi splitting around the molecular transition in both simulated and experimental (Supplementary Figure 33b, red curve) spectra for PDI nanobelts; this result suggests that the characteristic signature of polariton formation is precluded not only by the vibronic progression of PDI, but also the significant detuning between photonic and molecular states. In addition, while coupling between PDI and standing-wave modes $m \neq$ 2, 3, 4, 5 can also contribute to optical spectra, these contributions are expected to be insignificant compared to the more resonant light-matter interactions of the modes considered above.

We now discuss the design parameters that enable light-matter interactions in the PDI nanobelt system. If we increase the nanobelt width from 50 to 800 nm, standing-wave modes and their large coupling to the molecular transition are still obtained. However, if instead the nanobelt height is reduced from 800 to 50 nm, eigenmodes in the visible and near-IR regions lose standing-wave character, and light-matter coupling is apparent from neither the reflectance nor the absorbance spectra. These observations indicate that a minimum height of approximately half a wavelength of incident light is required for light-matter coupling to be observed in simulated reflectance spectra. Therefore, as stated in Supplementary Note 3, light-matter coupling and long-range energy transport should be a general feature of dielectric media with absorptive transitions. Indeed, the light-matter interaction in both PDI nanobelts and PIC film (Supplementary Figure 26) depends on nanostructure height in the same way that such interaction in microcavity exciton-polaritons depends on cavity transverse length: the

nanostructure height should be chosen to allow formation of standing-wave modes that are at or near resonance with the molecular transition to be coupled. In summary, the realization of light-matter coupling relies on the high oscillator strength inherent in J-aggregate dyes, a difference in refractive index of the dye from those of the surrounding, and optimal height of the dye layer [26].

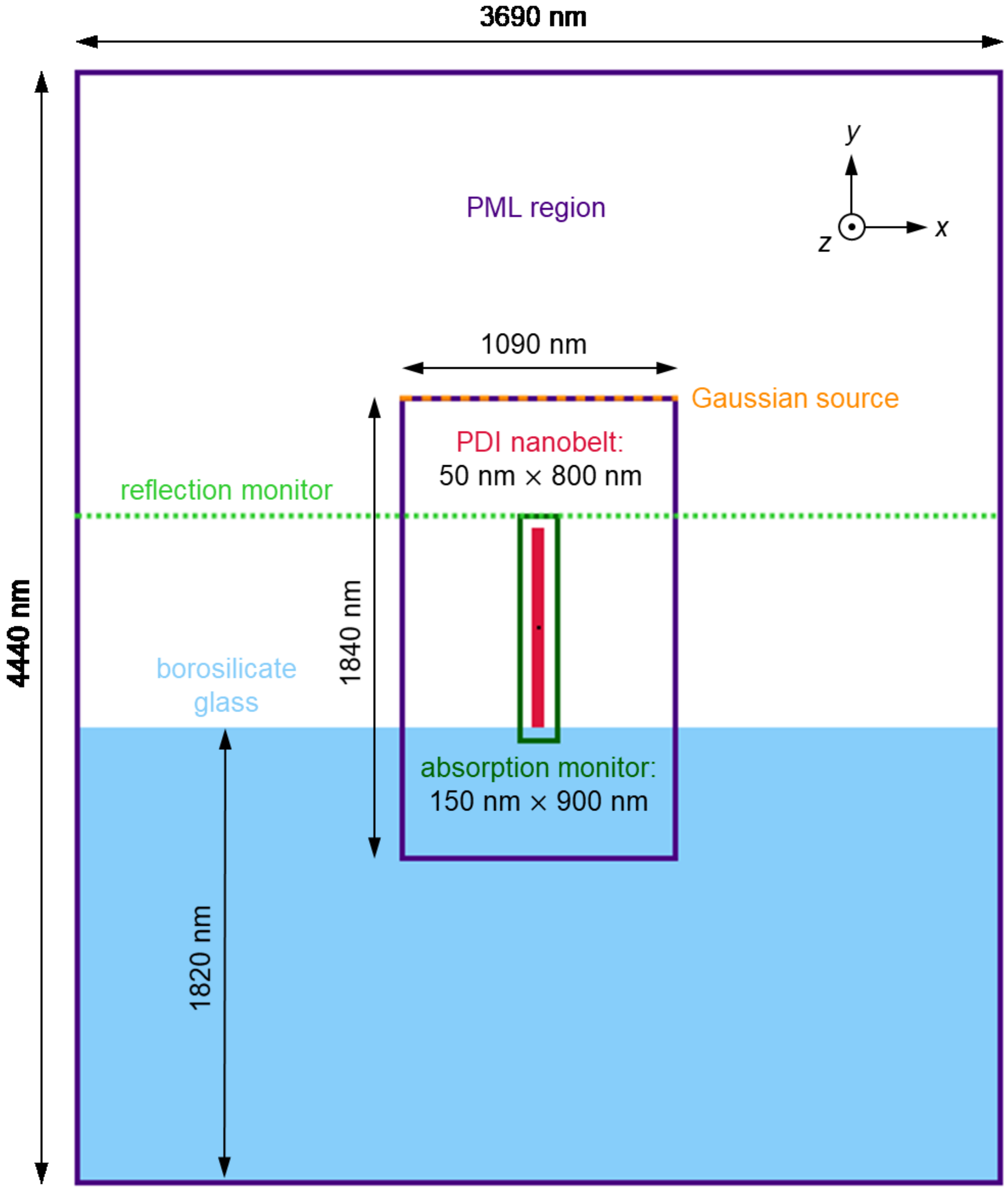

Supplmentary Figure 31: FDTD simulation cell. All rectangles shown, except for the rectangle representing borosilicate glass, are centred at the origin (black dot in the middle). The grid resolution in the simulation is 0.1 pixels/nm. Upon indicating *yz*-mirror symmetry in MEEP, the simulation cell is halved, and additional pixels may be added to facilitate this halving. For other software packages, the user may have to specify different and/or additional parameters pertaining to object lengths, numerical mesh, etc.

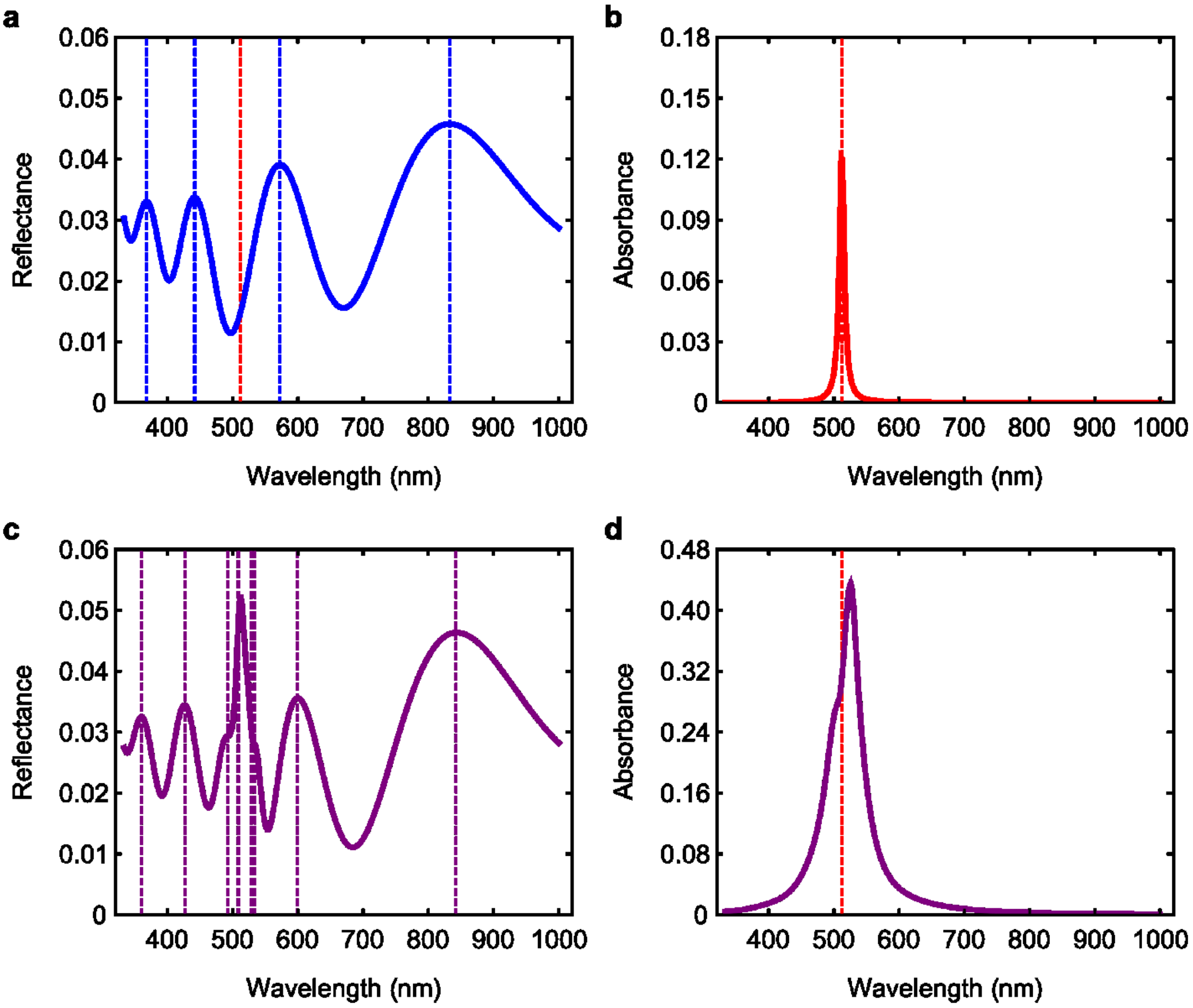

Supplementary Figure 32: FDTD-simulated spectra. a. Reflectance spectrum for J-aggregate material on glass without light-matter interaction between the electromagnetic modes of the material and the molecular transition at 512 nm. The blue dashed lines indicate the peak energies of the electromagnetic modes. The red dashed line indicates the energy of the molecular transition at 512 nm. **b.** Absorbance spectrum for system with relatively weak light-matter interaction, namely with the oscillator strength of the molecular transition being 1% of that in the system characterized in **c.** and **d**. **c.** Reflectance spectrum for the same system but with light-matter interaction between the electromagnetic modes of the material and the molecular transition at 512 nm. The purple dashed lines indicate the fitted polariton energies (see SI text). **d.** Absorbance spectrum for the same system as **c**. The red dashed line indicates the energy of the molecular transition at 512 nm.

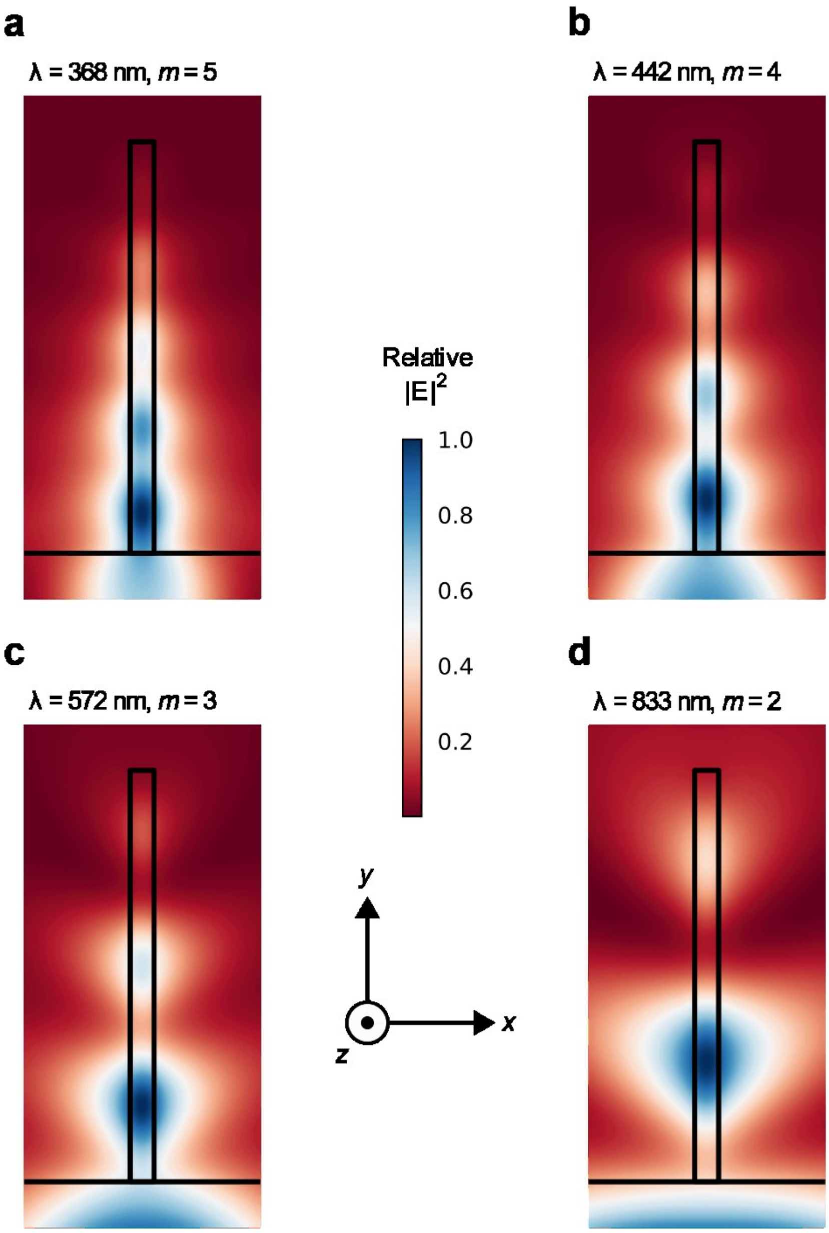

Supplementary Figure 33: Near-field distributions. Intensity of electric-field Fourier component with wavelength **a.** 368 nm, **b.** 442 nm, **c.** 572 nm, and **d.** 833 nm. The standing-wave index m characterizes the mode excited at each wavelength. For each plot, the intensities are normalized to the maximum, and the simulation was carried out using a Gaussian source whose centre and width equal the energy corresponding to the specified wavelength. A black rectangle denotes the PDI nanobelt, and all points below a spanning horizontal line lie in borosilicate glass. All points above the line but not in the black rectangle lie in vacuum.

Mode index m	Wavelength (nm)	Light-matter coupling strength $g_m\sqrt{N}$ (meV)	Mode volume V_m (V_{sample})	Strength of coupling to bath modes $\kappa_m/2$ (meV)

2	833	125	8.47	248
3	572	187	5.54	209
4	442	220	5.21	214
5	368	276	3.95	not determined [a]

[a]See SI text for details.

Supplementary Table 2: Light-matter coupling strengths, mode volumes, and strength of coupling to bath modes for photonic modes in system simulated in Supplementary Figure 32.

Supplementary Note 5: Rabi Flopping measurements

In Supplementary Figure 34-36 we show the results of laser fluence dependent measurements on the PL intensity of PDI nanobelts and isolated PDI monomers. In the case of multiple PDI wires no Rabi flopping can be observed due to ensemble averaging effects. Isolated PDI molecules in solution no Rabi flopping either because of rapid competing decay processes which are not mitigated by light-matter coupling.

To access the frequency content of the Rabi flopping, we first truncated the data to exclude field strengths < 9 x 10^8 Vm^{-1} and subtracted the slowly varying intensity with a 4-point B-spline fit. The residual was zero-padded to 256 data points before Fourier-transformation (Figure 2b). The same approach was carried out for the model.

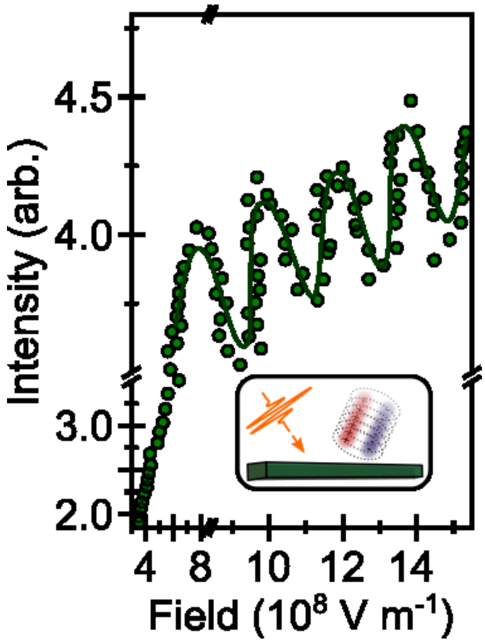

Supplementary Figure 34: Photoluminescence counts as a function of optical field for isolated PDI nanobelts as shown in the main text Figure 2c. The PL intensity initially increases linearly before oscillating as described in the main text. The fitted model (green overlay) shows good agreement with the experimental data

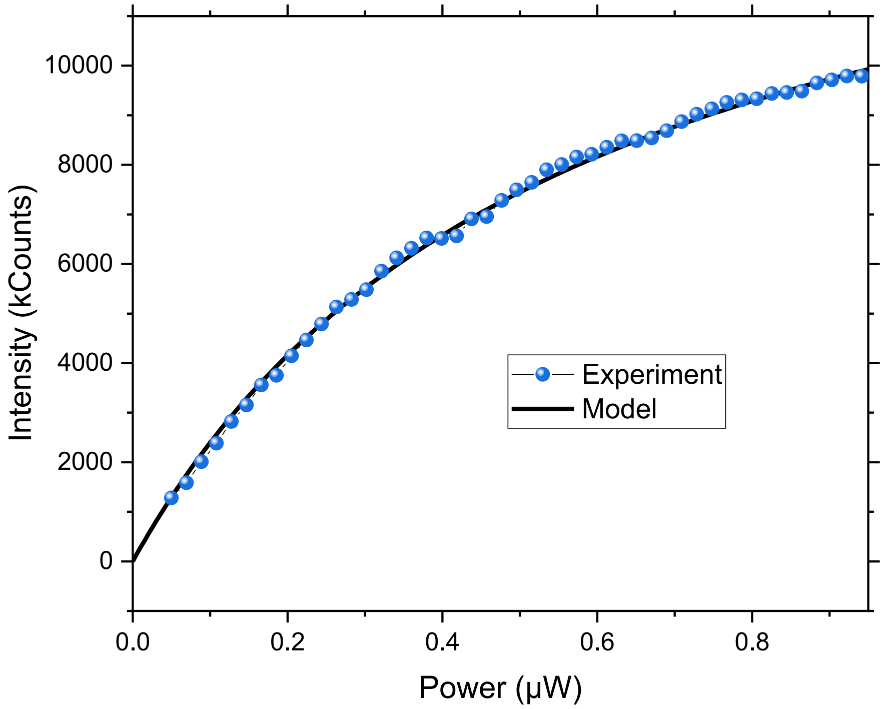

Supplementary Figure 35: Photoluminescence counts as a function of power for bundles of PDI nanobelts. Measurements were performed using a manner to that described in S1. The PL intensity initially shows quadratic increase (as expected from the fitted model – solid black line), before saturating beyond 0.8 μW.

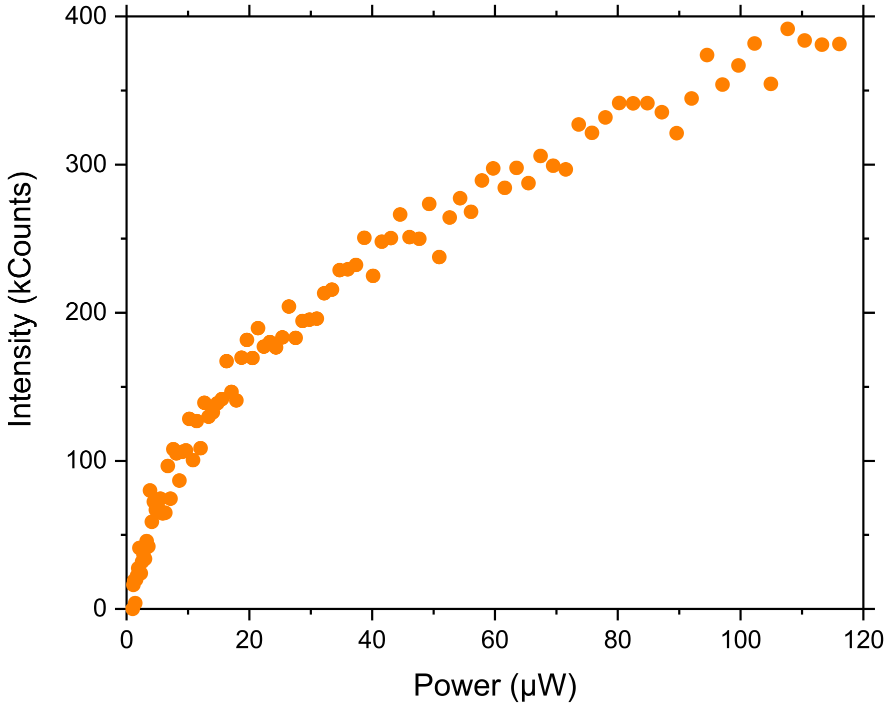

Supplementary Figure 36: Photoluminescence counts as a function of power for isolated PDI molecules. Measurements were performed using a manner to that described in S1but with focussing *via* a f=50 mm focussing lens and dye in a 1 mm cuvette (Hellma) that was sealed in Ar glovebox. All solvent used in preparation of the isolated dye molecules was degassed before usage. Data was obtained by averaging 3 up/down power sweeps. We assume negligible intersystem crossing occurs and the emission is from the singlet state. The PL intensity initially increases linearly before saturating after 30 μW.

Supplementary Note 6: Data analysis

Data acquisition

Consecutive $pump_{ON}$ and $pump_{OFF}$ images for each time delay were first corrected for camera background and then converted to a single $\Delta T/T$ image using the equation below:

$$\frac{\Delta T}{T} = \frac{pump_{ON}}{pump_{OFF}} - 1 \quad (5)$$

where $pump_{ON/OFF}$ denotes the background-corrected transmission image with and without the presence of the pump pulse. The extraction of time zero (t_0) is discussed later.

Self-referencing method

Due to the random nature of photon emission in a laser, fluctuations in the laser output power (i.e. shot noise) is unavoidable and may introduce significant noise to the final images. Although our system is shot-noise limited and the 15 Hz mechanical chopper should average out high-frequency fluctuations, low-frequency fluctuations should be observable through the varying background photon counts in the transmission. In order to mitigate these effects, we defined a correction factor for each pair of images, ε, as the ratio of averaged photon counts on the camera between the $pump_{ON}$ and the $pump_{OFF}$images and recalculated every $\Delta T/T$ image using a revised equation:

$$\left(\frac{\Delta T}{T}\right)_{new} = \frac{1}{\varepsilon} \frac{pump_{ON}}{pump_{OFF}} - 1 \quad (6)$$

Supplementary Figure 37 compares the $\Delta T/T$ intensity at a user-defined signal centre as a function of the sweep number before and after applying the self-referencing correction. It is evident that the average amplitude of the signal is not affected but the spikes are supressed. Applying this correction before averaging over multiple sweeps should further improve our SNR, as shown in Supplementary Figure 37 where we plot the $\Delta T/T$ signals before and after correction against time. The SNR can be improved by up to a factor of three depending on the experimental conditions. For example, airflow and vibrations induced body movement in the laboratory are sufficient to introduce significant fluctuations in the numbers of photons reaching the camera. This self-referencing correction was applied to all fs-TAM data presented in this manuscript.

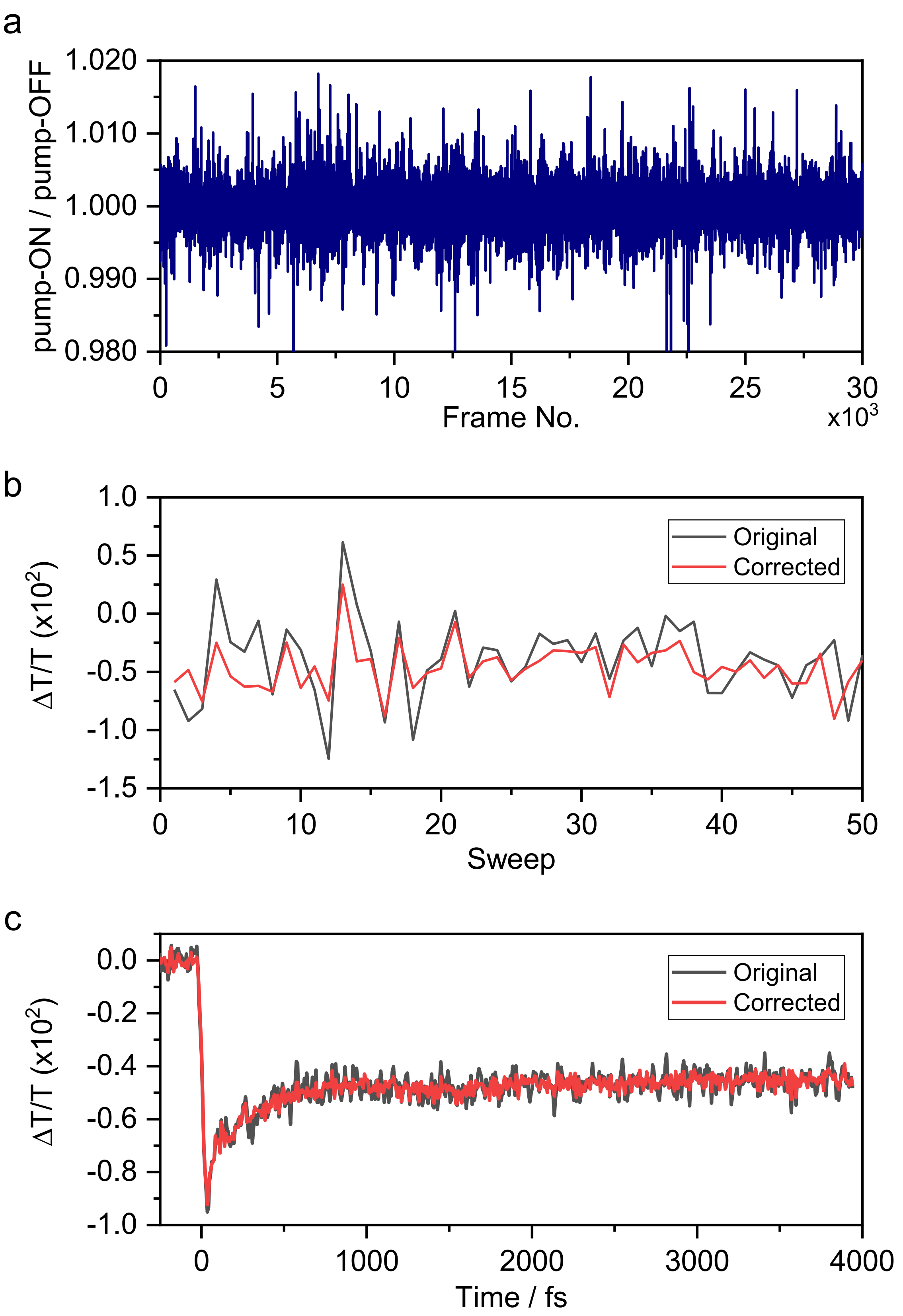

Supplementary Figure 37: a. The ratio of averaged photon counts between each pair of $pump_{ON}$ and $pump_{OFF}$ images, excluding all pixels affected by the actual signal and associated optical artefacts. This shows an averaged 0.4% fluctuation in the measured laser output with numerous spikes of over 1% in magnitude spanning the whole measurement window. **b.** $\Delta T/T$ response at a user-defined centre

in the image at t = 300 fs, plotted against the sweep number before and after applying the self-referencing correction. **c.** Kinetics at the same spot averaged over 50 sweeps before and after applying the self-referencing correction, showing a noticeable improvement in the sign-to-noise ratio.

A further background correction was performed on the averaged $\Delta T/T$ images to make sure that the average $\Delta T/T$ is zero before time zero. In practice, we estimated the background $\Delta T/T$ by averaging the first five images (i.e. 64 × 64 × 5 = 20480 pixels), which was subsequently subtracted from all images. The estimated background $\Delta T/T$ is usually on the 0.5×10^{-5} order of magnitude and has little effect on data analysis. Nevertheless, all TAM data presented in this work were background corrected.

Data smoothing - moving average

In order to suppress the random noise whilst preserving the real signal, we investigated the effects of applying a two-dimensional moving average filter to our data.

The moving average is the most common filter in digital signal processing. It operates by averaging several points from the input signal to produce each point in the output signal. For example, in a one-dimensional problem the output signal can be expressed mathematically as

$$y[i] = \frac{1}{N}\sum_{j=0}^{N-1} x[i+j] \quad (7)$$

where $x[i]$ and $y[i]$ are the i-th data point in the input and output signal respectively. This equation only uses a total of N points on one side of the signal. However, it is straightforward to use an equal number of points on either side of the output data point. For a two-dimensional dataset, the equation can be modified to average over data points surrounding the target point:

$$y[i,j] = \frac{1}{N^2}\sum_{k=0}^{N-1} x[i+k, j+k]$$

We applied the moving average filter to average over N^2 input data points surrounding the output point and assessed its performance as a function of N. Unsurprisingly, increasing N improves the noise reduction but the signal becomes less sharp at the edge. Similarly, applying the filter again further reduces the noise at the expense of signal strength and sharpness. We found that applying the filter once with $N = 3$ produces the ideal result with the best balance between preservation of the original signal and elimination of random noise.

Despite its simplicity, we find the 2D moving average filter consistently outperforms other conventional image analysis filter such as the 2D Fourier filter. As a result, we decided to adopt the moving average as our default post-processing filter. Unless otherwise stated, all TAM data was averaged once with the 2D moving average filter with $N = 3$.

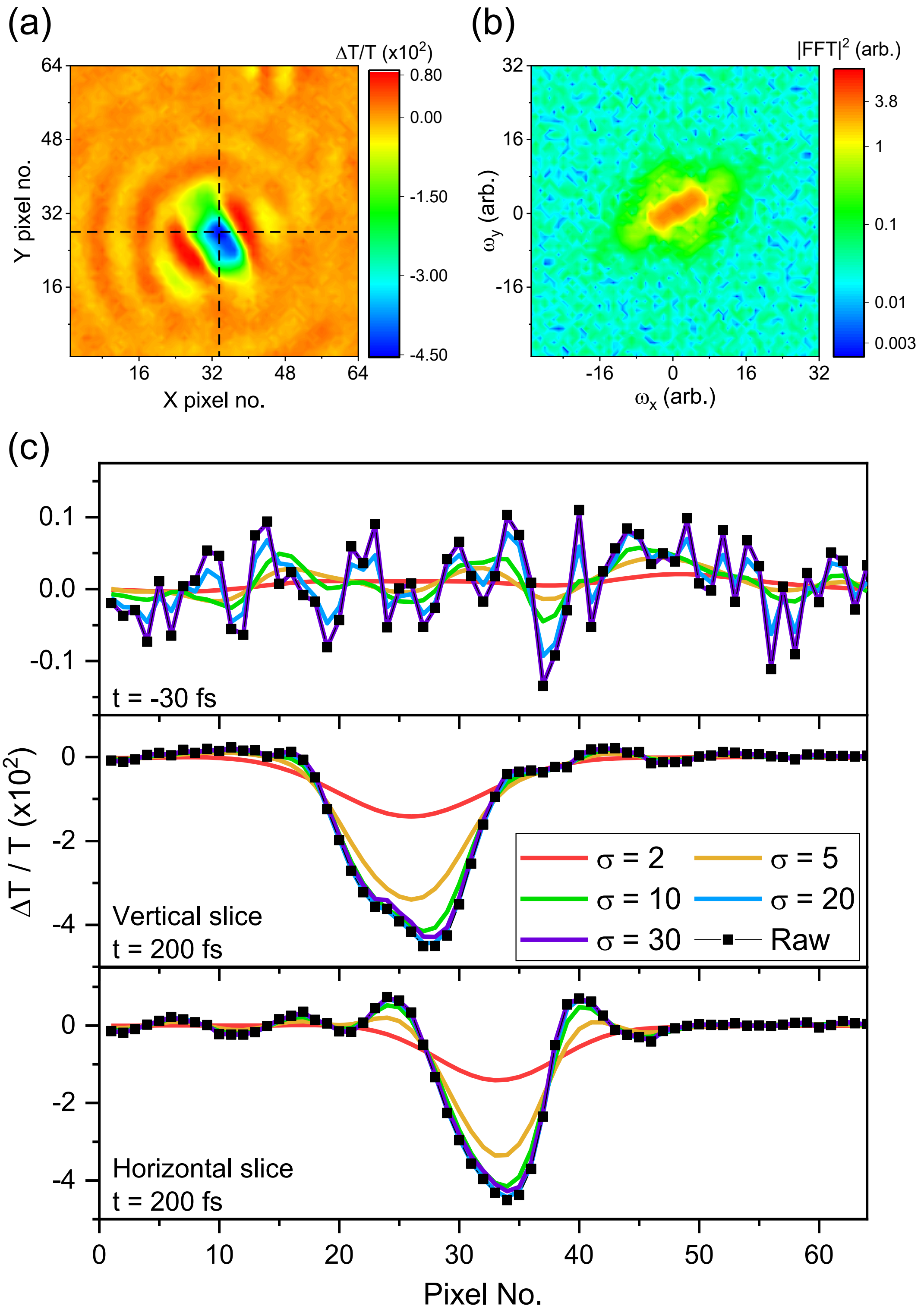

Supplementary Figure 38: a. A typical $\Delta T/T$ image from PDI sample with a pump-probe delay of 200 fs, showing a clear photo-induced absorption feature in the middle and the surrounding optical artefacts. The image was obtained by averaging over 50 sweeps. **b.** The same $\Delta T/T$ image in the frequency domain after a Fast Fourier Transform operation. The logarithmic colour scale helps to highlight the high-frequency noise, which is much weaker in amplitude. **c.** Effect of applying a Gaussian Fourier filter of a range of variances on the original signal. The top panel compares the ability of the different filters to remove the random noise from an image before time zero whereas the middle and the bottom panels

compare the effect of the filters on the actual signal amplitude and shape, plotted along the black dashed lines in **a**. It is clear that a filter with a large σ is able to preserve the signal better but it also lets more noise through.

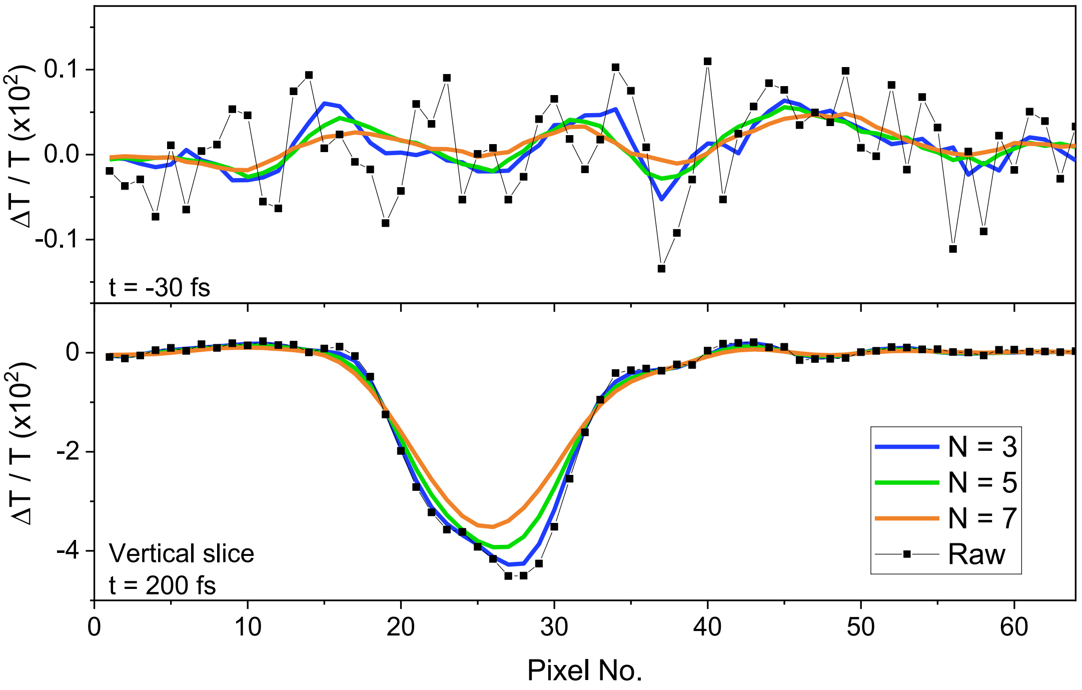

Supplementary Figure 39:.3Effects of applying a two-dimensional moving average filter of various window size N on the original signal (PDI). The top panel compares the ability of the different filters to remove the random noise from an image before time zero whereas the bottom panel compares the effect of the filters on the actual signal amplitude and shape. Surprisingly even with N = 3 the random noise is significantly suppressed and the improvement in noise reduction by increasing N is nullified by the loss in signal strength and shape as shown in the bottom panel.

Accurate determination of time zero, signal centre and sigma propagation

The most common method employed in analysis of TAM data is by the fitting of a two dimensional (2D) Gaussian [27,28]. To extract the material's diffusion constant D along a principle axis, one simply fits the diffusion length equation to the sigma-time graph. Assuming the initial population at $t = t_0$ is Gaussian in space with an initial width $\sigma(t_0)$ and undergoes anomalous diffusion, the sigma at any later time $t \geq t_0$ can be described by

$$\sigma^2(t) = \sigma^2(t_0) + D(t - t_0)^n \ (8)$$

where $n = 1$ for a typical diffusion process. $n < 1$ and $n > 1$ represent a sub-diffusion and super-diffusion process respectively. Sub-diffusion usually suggests the existence of carrier traps whereas super-diffusion is commonly found in active cellular transport. If $n = 2$ a particle undergoes ballistic transport with the transport length determined by its mean free path.

However, although this method produces quantitatively similar results it is not necessarily the most applicable to the quasi one-dimensional systems investigated, due to their inhomogeneous geometry. We therefore need to analyse our data differently, yet the extracted diffusion coefficient must still be comparable to those acquired from a 2D Gaussian fitting.

General method

The above equation describes how the width of the Gaussian grows in time. In a similar fashion, we can define σ as the distance between the centre of the signal and the point in the image plane (in any direction) where the signal strength is $e^{-1/2}$ of that at the centre. Mathematically this is expressed by

$$\sigma^2(\theta, t) = \left(x_{\frac{1}{2}}(\theta, t) - x_0\right)^2 + \left(y_{\frac{1}{2}}(\theta, t) - y_0\right)^2 \ (9)$$

where (x_0, y_0) and $(x_{\frac{1}{2}}(\theta, t), y_{\frac{1}{2}}(\theta, t))$ are the coordinates of the signal centre and the points where the signal strength at time t drops to $e^{-1/2}$ of that at the centre at angle θ relative to the positive x-axis, respectively. We will refer to $(x_{\frac{1}{2}}(\theta, t), y_{\frac{1}{2}}(\theta, t))$ as the 'sigma points' from now on. We can then extract the diffusion coefficient and investigate if the carriers diffuse more in a certain direction.

Any scientific statement or comparison of parameters is valid only when it is significant compared to the relevant uncertainties. Therefore, it is important that we evaluate the uncertainties in our derived quantities and minimise them by adopting the best methods in data analysis. In the presence of random noise, one can estimate the uncertainty in the x- and y-coordinates to be 1 pixel (i.e. 55.5 nm on the

image plane). Therefore, each direction in equation 9 will have a combined error of about 1.4 pixels (i.e. 80 nm) and the overall uncertainty σ^2 will be over 110 nm. Consequently, all the coordinates must be determined with an accuracy and precision better than our pixel resolution, or else the large uncertainties in the coordinates will render σ^2 and any other fitted parameters un-usable. Another reason for correct determination of the centre is that equation 9 is non-linear, which means that an inaccurate centre may skew the fitted parameters differently as angle θ varies and makes fair comparisons difficult.

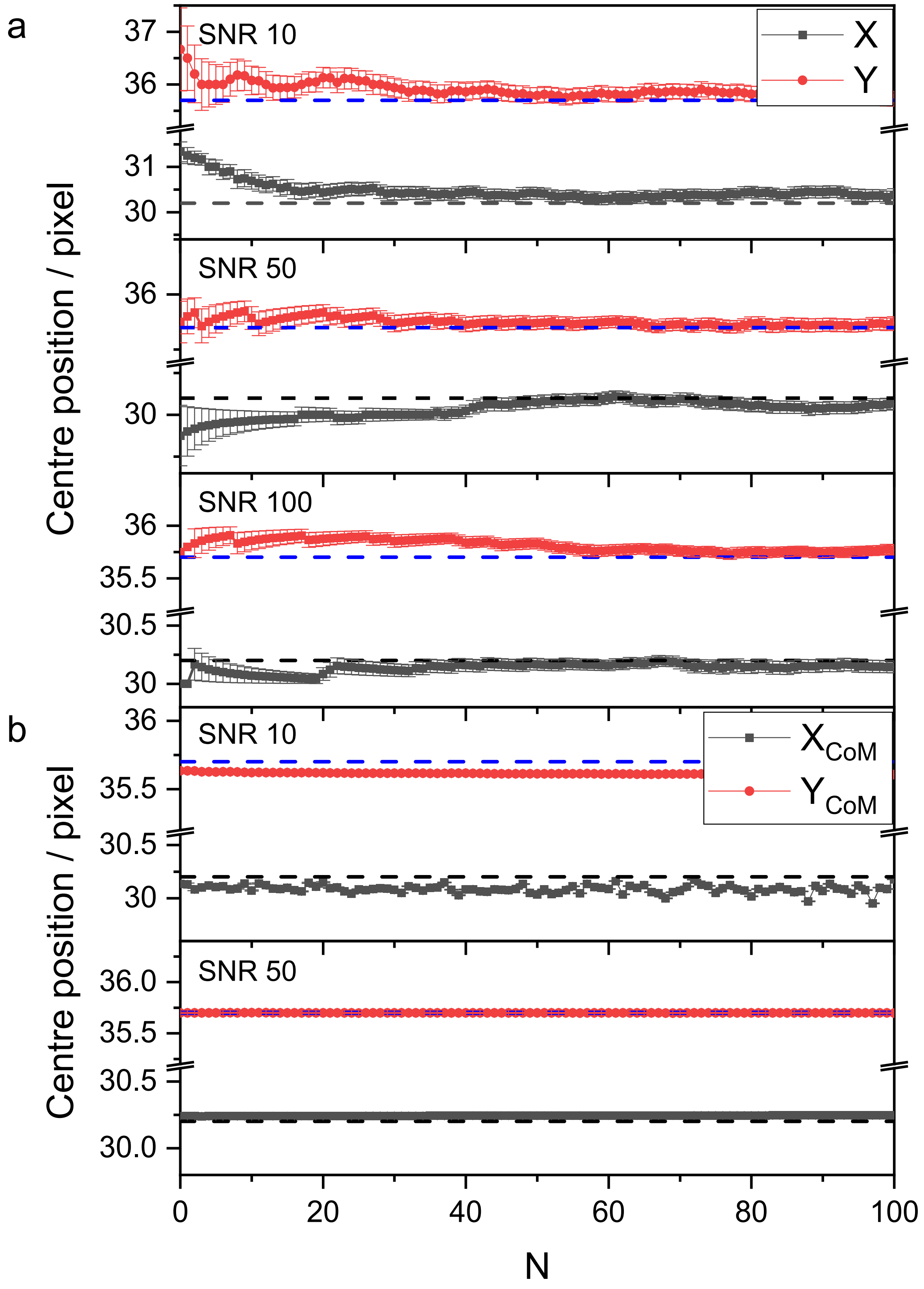

a
SNR 10
X
Y
37
36
31
30
SNR 50
36
30
SNR 100
36
35.5
30.5
30
b
SNR 10
X_{CoM}
Y_{CoM}
36
35.5
30.5
30
SNR 50
36.0
35.5
30.5
30.0
Centre position / pixel
Centre position / pixel
0
20
40
60
80
100
N

Supplementary Figure 40: Estimated position and uncertainties of the signal centre in datasets with various signal-to-noise ratios, using **a.** Method 1: averaging the positions of the pixel with maximum signal and **b.** Method 2: calculating the centre of mass (CoM) in each frame, then averaging over N images after time zero. The blue and black dashed lines represent the true positions of the centre: (30.2, 35.7). Method 1 is much more sensitive to N, and requires a much larger number of frames (more than 50) to achieve a stable and accurate estimate.

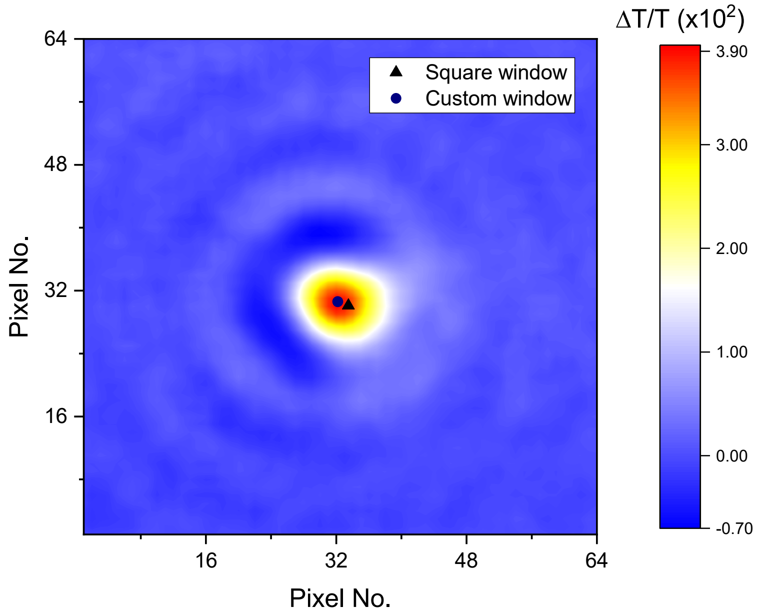

Supplementary Figure 41: A typical TAM image from PDA, showing the effect of using a square window (i.e. including pixels with a strong negative $\Delta T/T$) and manually selecting pixels in the centre-of-mass (CoM) calculations. The negative C-shaped signal on the left is probably an optical artefact due to the microscope objective or inhomogeneous surface morphology. Note that it pushes the CoM away from where we would believe the true pump focus was. By manually selecting the pixels with a strong positive $\Delta T/T$ signal, we are able to retrieve a CoM much closer to our expectations.

Determination the signal centre

It is tempting to use the pixel with the strongest signal in each frame as the centre, as one might expect its position to be constant over time. However, since one pixel on the EMCCD camera corresponds to 55.5×55.5 nm^2 in real space and the pump spot has a FWHM of ~270 nm, the central four pixels often receive similar numbers of photons. In practice, the position of the maximum signal pixel fluctuates between 1-2 pixels in either direction. This is undesirable for not only the reasons described in the previous section, but physically the centre of the signal is defined as the centre of the pump pulse, which has a well-defined position that stays constant throughout the measurement window. There are two

common ways to achieve sub-pixel resolution and improve accuracy: 1) Average the coordinates of the pixel with maximum signal strength over a number of frames; 2) Determine the centre using multiple pixels within the same image. Method 1 works in a similar way to dithering in digital signal processing, where uncorrelated noise is added purposefully to an analogue signal before digitisation to reduce quantisation noise. The random noise present in our data pushes the signal maximum to neighbouring pixels in time. Therefore, averaging over many frames should yield a more precise estimate of the true position of the signal centre. We tested the method using simulated TAM data with a known centre at (30.2, 32.5) and a range of maximum SNR from 10 to 100. The estimated centre coordinates are plotted in Supplementary Figure 40 as a function of N, which is the number of frames averaged. The errors on each averaged coordinates is taken as the standard error in mean given by $\sigma_{\bar{x}} = \frac{\sigma_x}{\sqrt{N}}$. We found that a large number of frames (more than 50) is required to achieve a stable and accurate average, even for a dataset with a high SNR. As a result, the averaged position will not reflect the true pump centre. This method also requires significant noise to push the signal to neighbouring pixels efficiently, which is not always available if the SNR is too high especially after smoothing. Method 2, on the other hand, utilises a number of pixels around the maximum to estimate the centre through a weighted average. It works almost identically to calculating the centre of mass (CoM) of N discrete masses on a plane, which can be expressed algebraically using the following equations:

$$X_{CoM} = \frac{1}{M}\sum_{i=1} m_i x_i \quad (10)$$

$$Y_{CoM} = \frac{1}{M}\sum_{i=1} m_i y_i \quad (11)$$

where $(X, Y)_{CoM}$ is the coordinates of the centre of mass, M is the total mass, m_i and (x_i, y_i) are the mass and coordinates of the i-th individual mass respectively. To calculate the centre of our signal, we substitute the $\Delta T/T$ data points for the individual masses in a 10 × 10 square window surrounding the maximum signal pixel (i.e. $i_{max} = 100$). We can then average the CoM over several frames and calculate its uncertainty in a similar fashion. Using the same set of data, it is clear from Supplementary Figure 25 that the CoM calculated from each frame is much closer to true value and the frame-to-frame fluctuation is much reduced (less than 0.1 pixels in each direction) when compared to that of Method 1 (1 pixel in both directions). Therefore, a stable CoM was achieved using a significantly smaller number of frames. In fact, there is no need to average over multiple frames as the estimated centre is very close to the true value using a single frame. The slight drifts in the estimated centre are most likely caused by a slightly asymmetrical selection of pixels around the true maximum. However, in most cases, the magnitude of such drift is less than 0.1 pixel (around 5 nm in the sample) so this is not of serious concern.

It is important to note that if the $\Delta T/T$ value of a pixel has an opposite sign to the main signal, it acts as a 'negative' mass which pushes the centre of mass away from the true position. This can sometimes significantly skew the centre when 'Airy disk rings' are much more pronounced on one side of the

signal. The solution to this problem is to exclude the pixels heavily influenced by the 'Airy disk rings'. As a result, we adopted method 2 as our default method to define our signal centre (Supplementary Figure 41).

<u>Determine sigma at different time points and angles</u>

After we have estimated the signal centre, we need to extract the sigma points (i.e. coordinates where the signal strength is $e^{-1/2}$ of that at the centre), at any angle and time with sub-pixel resolution. This is achieved by mapping the smoothed data onto a fine polar grid centred at $(X, Y)_{CoM}$, followed by selecting in each direction the point which has an amplitude closest to $e^{-1/2}$ of that at the centre. $\sigma^2(\theta, t)$ can then be calculated.

We investigated the effect of noise on the errors in the CoM and $\sigma^2(\theta, t)$. A circular Gaussian signal with a constant width and strength in time (a minimum of 250 frames) was corrupted with random noise such that the signal-to-noise ratio ranges from 1 to 100. The CoM and $\sigma^2(\theta, t)$ for each frame are calculated as above. The uncertainty in the CoM is taken as the standard deviation of the coordinates computed from 250 frames. σ was calculated for 180 angles in each frame and the uncertainty is taken as the standard deviation of the 250 × 180 = 45000 values obtained. Supplementary Figure 42 summarises the findings and it is clear that the uncertainties decreases rapidly as the SNR improves (almost linearly on a log-log scale plot). The CoM has an impressively low uncertainty even when the SNR is 1. On the other hand, we need a minimum SNR of 10 to achieve an uncertainty of less than 1 pixel in σ. This suggests the importance of having a good SNR if we wish to extract the diffusion parameters accurately from TAM data. It is important to note that these uncertainties are associated with individual CoM coordinates and σ points at a particular angle. By averaging the CoM and σ points over multiple frames and angles respectively will further reduce the errors by a factor of $\sqrt{N}$.

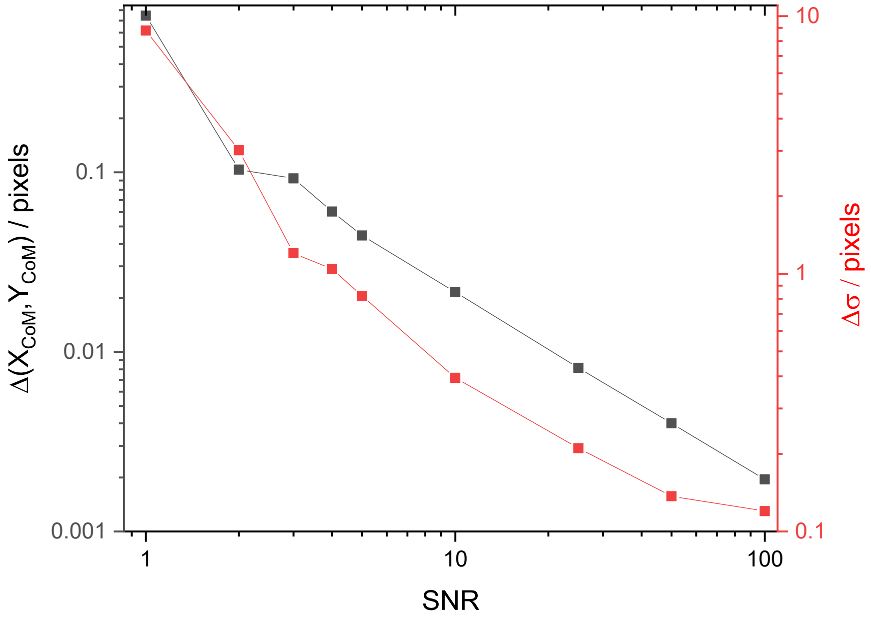

Supplementary Figure 42: Uncertainties in the centre of mass (CoM) and σ plotted against signal-to-noise ratio (SNR). The uncertainties fall off rapidly as the SNR improves from 1 to 3. Random noise has little effects on the CoM even for a low SNR of 1. On the other hand, a minimum SNR of 10 is required to reduce the uncertainty in σ to below 1 pixel.

Extraction of time zero (t_0)

In order to estimate the diffusion coefficient using E1, we need to define time zero (i.e. the moment when the pump pulse arrives at the sample), for which three common definitions exist: The moment when the $\Delta T/T$ signal 1) appears through the noise; 2) is half-way to the maximum and; 3) is at the maximum. Placing time zero too early may result in sigma increasing rapidly at early times and an illusion of ultrafast diffusion process as the signal grows through the noise. On the contrary, putting time zero too late in time would mask any ultrafast carrier processes (e.g. ballistic transport). Hence, it is crucial we can detect time zero with confidence. It is not always straightforward to choose one of the above definitions to find time zero. For example, the observed $\Delta T/T$ signal could keep growing in for hundreds of fs (or even longer) if we were probing a photo-induced absorption whose growth in space and time depends on the exact dynamics of two different populations (e.g. individual lifetimes, rates of interconversion and annihilation, diffusion properties, etc.).

One interesting observation when we plot the extracted sigma points on top of the TAM images, as illustrated in Supplementary Figure 43, is that the algorithm is unable to define the sigma points before

time zero. Before the signal comes in, the sigma points are scattered randomly over the whole image, giving a large average distance from the estimated centre $\bar{\sigma}$, as shown in Supplementary Figure 44. As the signal starts to grow in, as shown in the middle image in Supplementary Figure 44 (Frame 43), the sigma points gradually aggregate into an elliptical ring. The smoothness improves and $\bar{\sigma}$ increases, as the signal gets stronger within a few frames (i.e. within 20 fs). We decided to define time zero as the first frame where the ring formed by the sigma points is smooth and has a $\bar{\sigma}$ closest to the diffraction-limited pump spot size ($2\bar{\sigma} \approx 1.7 \times FWHM \approx 1.7 \times \frac{\lambda}{1.1NA}$). This is crosschecked with the signal strength at the CoM and also the t_0 obtained by fitting the electronic response y ($\Delta T/T$ at CoM pixels) with the following multi-exponential equation:

$$y = \sum_n \frac{1}{2} \cdot A_n \cdot e^{\frac{s^2}{2\tau_n^2}} \cdot e^{\frac{-(t-t_0)}{\tau_n}} \cdot \left(1 + erf\left(\frac{t-t_0-\frac{s^2}{\tau_n}}{\sqrt{2}s}\right)\right) \quad (12)$$

here n is the number of lifetimes τ to be extracted, s is the instrumental response and A_n is the amplitude of the n-th exponential decay. The error function considers the probe arriving at $t = t_0$ is Gaussian temporally. For all data considered in this manuscript t_0 is obtained from this method lies at or beyond the half way point in the signal rise. Consequently, we find this to be a reasonable and consist approach to find time zero.

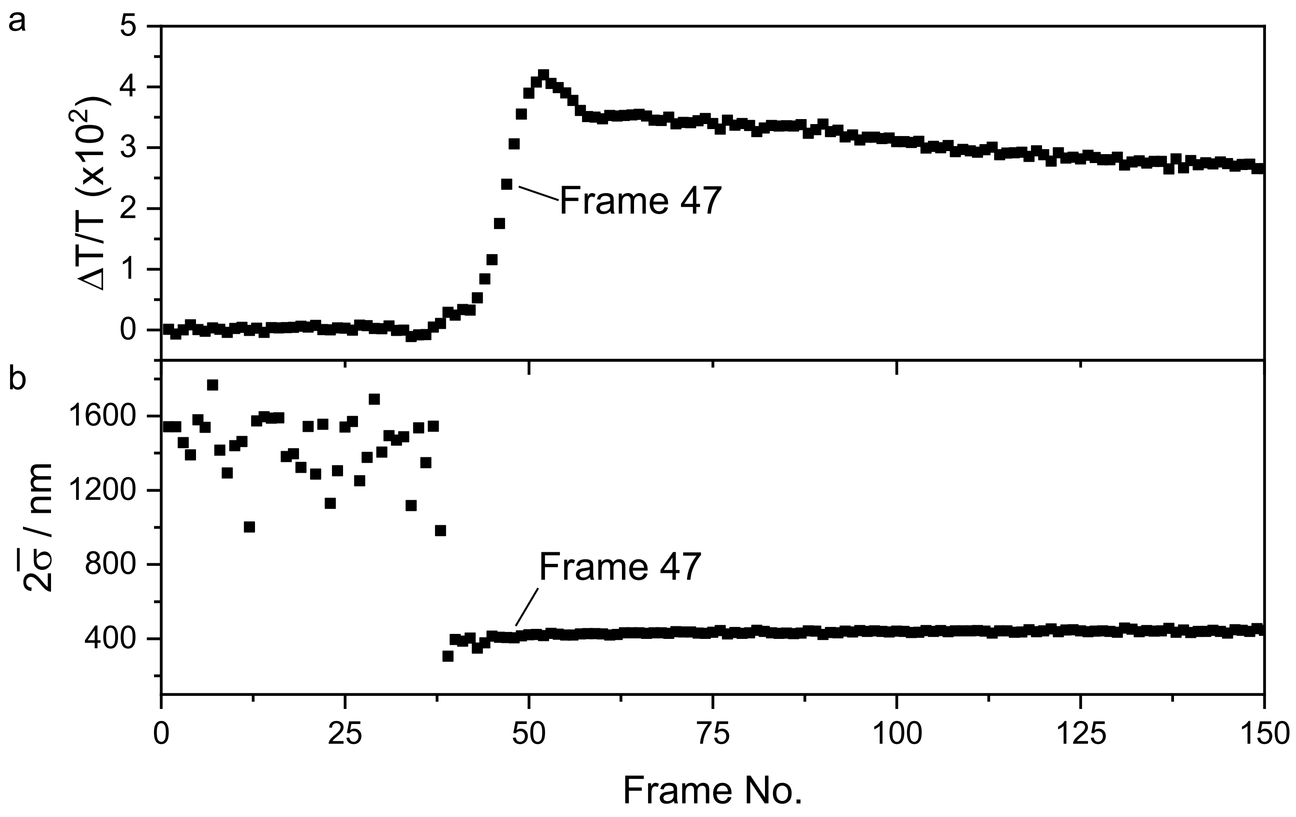

Supplementary Figure 43: a. Signal strength at the centre of mass. **b.** average diameter of the ring formed by the sigma points plotted against the frame number. Before time zero, the sigma points are randomly scattered across the image resulting in a large average distance from the centre. As the signal starts to appear, a ring is formed with its diameter growing with the signal. In the case with no carrier diffusion, the signal should have a constant size approximately equal to that of the pump spot ($2\bar{\sigma}{\sim}400\ nm$) throughout the measurement window.

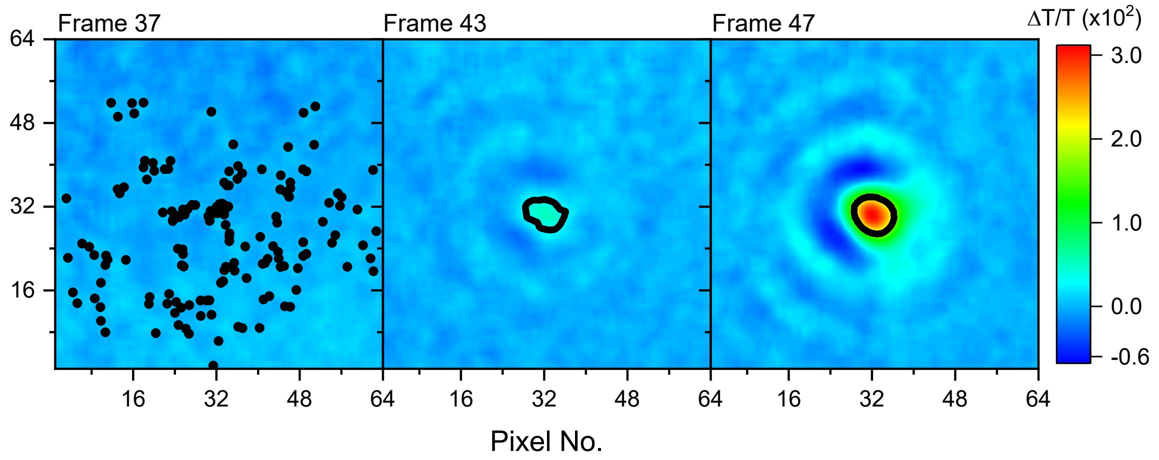

Supplementary Figure 44: Sigma points plotted on top of the corresponding TAM images with the same colour scale. (Left) The sigma points are scatter randomly over the whole image before time zero; (middle) a ring starts to form as the signal begins to grow; (right) the first frame when the ring is smooth and has a spot size very close to that of the diffraction-limited pump. Consequently, we define Frame 47 as our time zero.

<u>Spot size at time zero</u>

The spot size at time zero is determined from the method outlined in the above sections. The values obtained across all measurements of PDA, PIC and PDI are shown in Figure **SI29**. In all cases the FWHM at t_0 lies in the range 300 – 460 nm, with mean values of $\langle FWHM(t_0)_{PDA}\rangle$ = 362 ± 25 nm, $\langle FWHM(t_0)_{PIC}\rangle$ = 367 ± 25 nm and $\langle FWHM(t_0)_{PDI}\rangle$ = 365 ± 25 nm obtained. These values lie in the expected range based on the numerical aperture of the objective, pump/probe wavelength and calibration of the pump spot size using fluorescent microspheres, showing this method for determining $FWHM(t_0)$ to be robust.

a

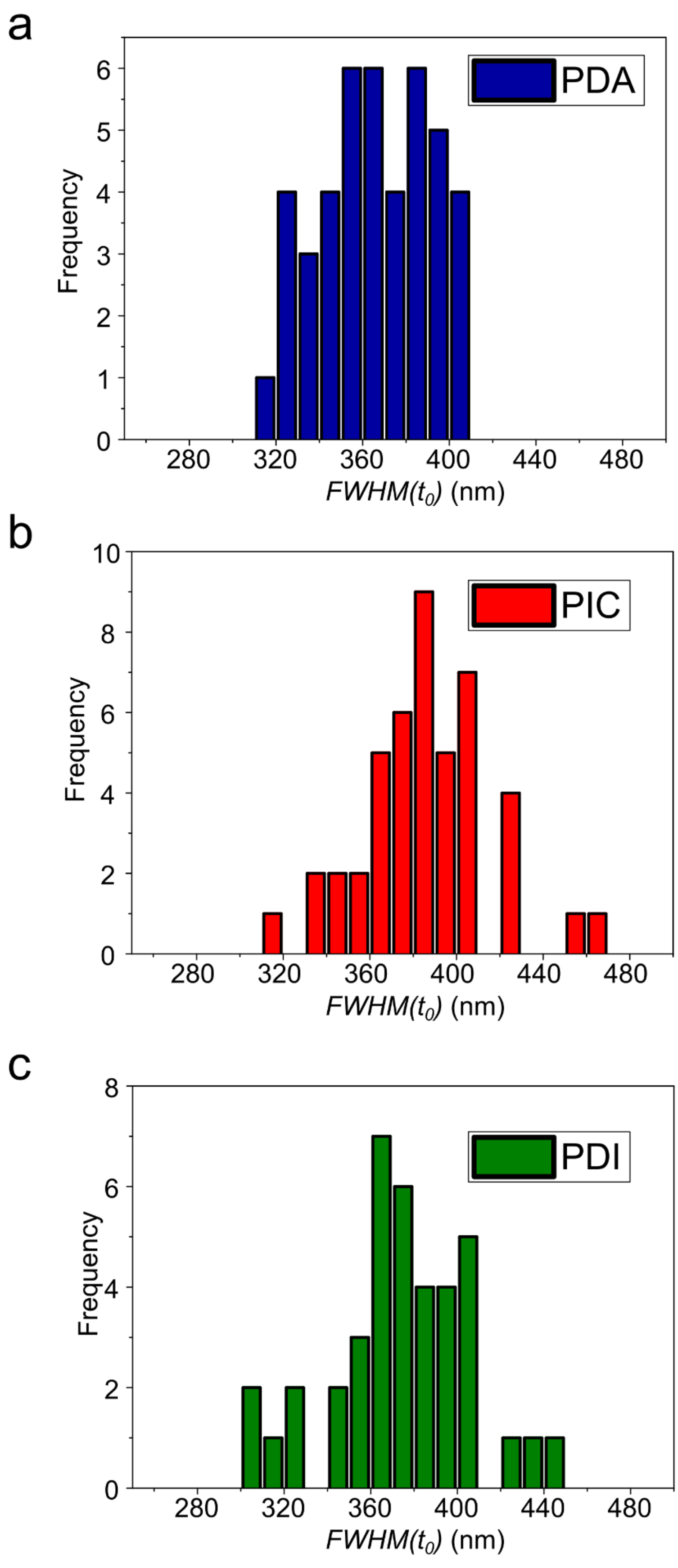
PDA
Frequency
0
1
2
3
4
5
6
280
320
360
400
440
480
FWHM(t_0) (nm)

b

PIC
Frequency
0
2
4
6
8
10
280
320
360
400
440
480
FWHM(t_0) (nm)

c

PDI
Frequency
0
2
4
6
8
280
320
360
400
440
480
FWHM(t_0) (nm)

Supplementary Figure 45: a-c. Histograms showing distribution of pump spot size at t_0 for all sample locations reported in the main text. In all cases, the mean size lies close to the diffraction limit based on the numerical aperture of the objective and pump wavelength.

Radial analysis of TAM kinetics

To track the rise and decay of spatially migrating population it is possible to take a ring cut through a stack of fs-TAM images. Because the signals arising from the structures considered in this manuscript are typically highly anisotropic, ellipsoidal as opposed to circular cuts were taken. The results of such a fitting method are shown in Supplementary Figure 46-48. The line thickness of ellipses in each reflect the number of pixels averaged over to obtain kinetic for that particular ellipse, typically 0.5 - 1.5 pixels radially. The radius of the ellipse reported in the legend is that for the semi-major axis, as determined from the centre of mass pixel.

For PDA, PIC and PDI the growth of the kinetic from the outermost ellipse is delayed compared to that obtained by averaging pixels around the centre, and reflects the time taken for spatial transport of population. For PDA specifically, the signal at 500 nm or 240 nm (Supplementary Figure 46) shows a significantly slower rise (τ = 70 ± 5 fs - a; 100 ± 5 fs - b) compared to that at the centre, suggesting that in this material there are intermediate electronic relaxation processes involved in the transport of energy. The decay rate of the signal at 500 nm or 240 nm is also much slower than that at the centre (τ = 230 ± 10 fs - a; 620 ± 5 fs - b), where the kinetic of the decay is similar to that obtained from ensemble spectroscopy. We note simulations (Supplementary Note 9) show that annihilation effects play little role in the observed dynamics and constantly cannot be responsible for the fast decay in the central region. Consequently, it is suggested that in PDA the transported signal at the edge pixels of the profile could arise from a state that is different to that which is within the pump excitation area.

For PIC the signal from the edge pixels decays at a similar rate to that in the centre, with the rise off-set due to energy migration. However, the decay of the kinetic, as discussed later, is much slower that obtained from ensemble spectroscopy. In PDI depending on the exact sample location very different kinetic profiles can be obtained. For example, in Supplementary Figure 47a the pump-probe signal at 10 nm and 190 nm are off-set but decay at a similar rate (τ = 220 ± 2 fs), with the rise of the former marginally slower (τ = 15 ± 2 fs - 10 nm; 28 ± 2 fs – 190 nm). However, for the sample location examined in Supplementary Figure 47b the signal at 250 nm decays far slower than that at 10 nm (τ =

95 ± 10 fs - 10 nm; 930 ± 10 fs – 250 nm) suggesting either a decay from a different electronic state at the edge pixels or a slow underlying transport of population to the ends of the wire that is convoluted with decay of the signal.

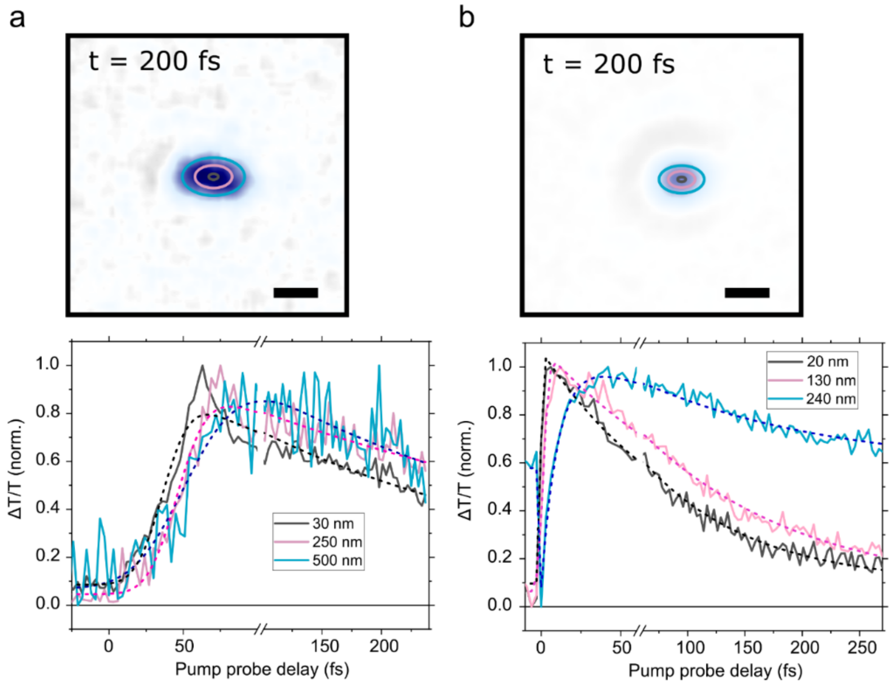

Supplementary Figure 46: a-b. Radial analysis of fs-TAM data for PDA at two different sample locations. Images are shown for a constant time delay of 200 fs. Ellipsoidal ring cuts are taken through the image and pixels on the ellipse averaged to generate the kinetics shown beneath. We average pixels along the lines shown in the top images. The distance shown in legend corresponds to the radius along the semi-major axis ellipse. The rise and decay of the kinetic at increasing distances from the centre is delayed with respect to that at the centre. In some cases, the strong Airy disks prevent absolute extraction of the signal (e.g. the signal before 0 fs in the 240 nm kinetic of panel b normalizes to 0.6). Solid lines show raw data whereas dotted lines are fits. Scale bars in both top images corresponds to 500 nm.

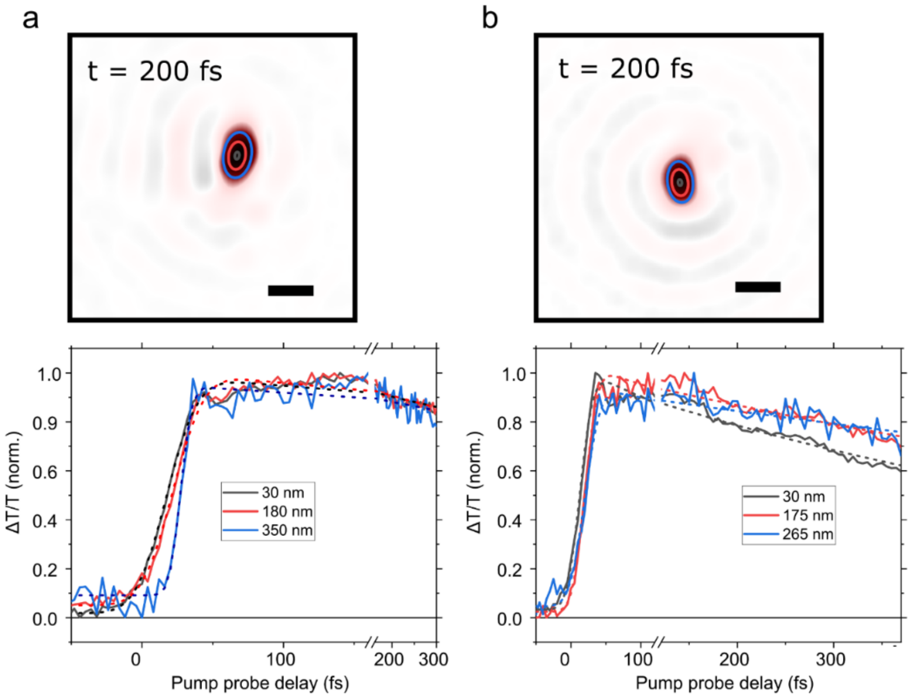

Supplementary Figure 47: a-b. Radial analysis of fs-TAM data for PIC at two different sample locations. Images are shown for a constant time delay of 200 fs. Ellipsoidal ring cuts are taken through the image and pixels on the ellipse averaged to generate the kinetic shown beneath. We average pixels along the lines shown in the top images. The distance shown in legend corresponds to the radius along the semi major axis of the ellipse. The decay of the kinetics is approximately the same at different distances from the centre but the rise of the signal away from the centre is delayed, indicative of ultrafast energy migration. Scale bars correspond to 500 nm.

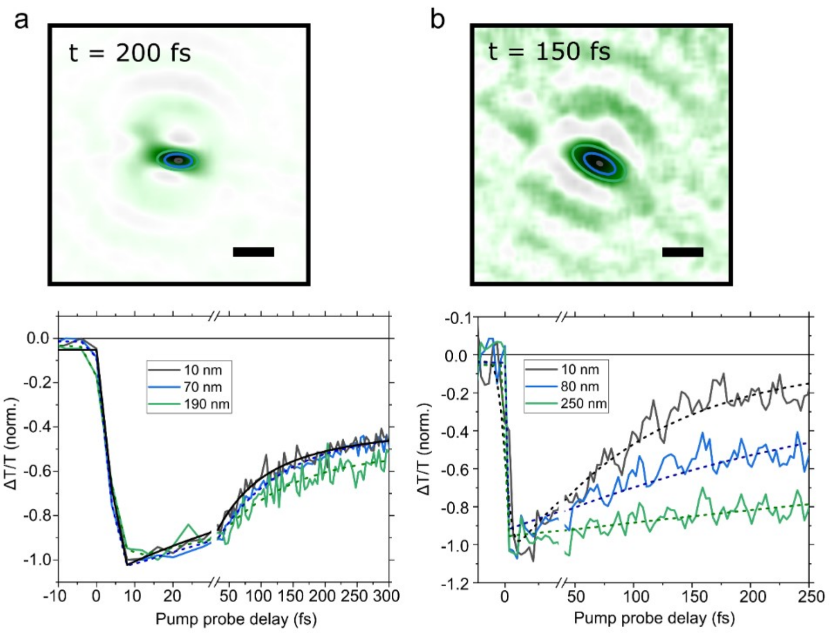

Supplementary Figure 48: a-b. Radial analysis of fs-TAM data for PDI. Ellipsoidal ring cuts are taken through the image and pixels on the ellipse averaged to generate the kinetic shown beneath. We average pixels along lines shown in the top image. The distance shown in legend corresponds to the radius of the ellipse along the semi major axis. The decay of the kinetics can be approximately the same (**a**) at different major radii or very different (**b**) depending on the sample location, but the rise of the signal is delayed in all cases, indicative of ultrafast energy migration. Scale bars correspond to 500 nm. The strong oscillations atop of the kinetic in (**b**) are Raman modes. The analysis and discussion of this is however beyond the scope of this work.

Fitting with two-dimensional Gaussian function

A two-dimensional (2D) rotational Gaussian function can alternatively be fit to each frame and used to analyse the spatial expansion of the $\Delta T/T$ signal. Extracting the Gaussian standard deviation (σ) along the two principle components of the 2D Gaussian, nominally referred to as x and y and plotting the mean square displacement, $\sigma(t)^2 - \sigma(t_0)^2$, against time allows us to determine the transport velocities and diffusion coefficients, $D_{x,y}$, in the two principle directions. In Supplementary Figure 49 we compare the D values obtained from this analysis, $D_{2D\ Guass}$, to the radial method D_{Radial} (along the wire axis), detailed previously in the main text. As the growth of the signals is typically anisotropic, we take the x direction as that in which there is maximal growth. The solid straight line denotes $D_{2D\ Guass} = D_{Radial}$. It is clear that the D values obtained from the two methods are roughly equivalent with some small variation in the exact nature of the MSD plot.

We emphasise here that order to fit a 2D Gaussian the excited species must not see boundaries which might affect the distribution of population in space. As we are dealing with 1D nanostructures whose boundaries inherently lie within the area of the pump spot the boundaries of the material can skew the 2D Gaussian and hence it is not an appropriate function to capture the physics, despite the reasonable fit.

The error on the fits is not shown on the plots for clarity, but was estimated using the MATLAB curve fitting tool box; fits were considered acceptable when the error was less than 5%.

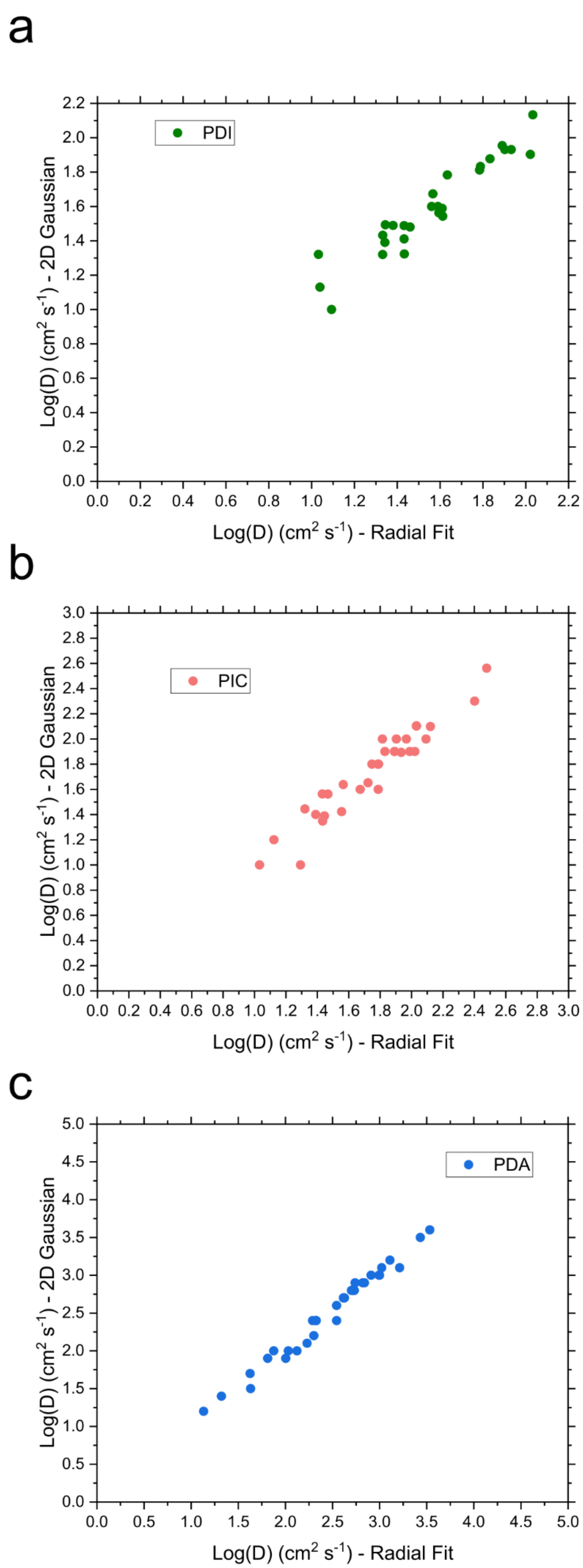
a
PDI
Log(D) (cm^2 s^{-1}) - 2D Gaussian
Log(D) (cm^2 s^{-1}) - Radial Fit
b
PIC
Log(D) (cm^2 s^{-1}) - 2D Gaussian
Log(D) (cm^2 s^{-1}) - Radial Fit
c
PDA
Log(D) (cm^2 s^{-1}) - 2D Gaussian
Log(D) (cm^2 s^{-1}) - Radial Fit

Supplementary Figure 49: Comparison of diffusion coefficient obtained from radial and 2D Gaussian fitting, for **a.** PDA (blue), **b.** PIC, (red) and **c.** PDI (green). Diffusion constants are obtained for R_2 region. There is a near one-to-one correspondence between the two methods.

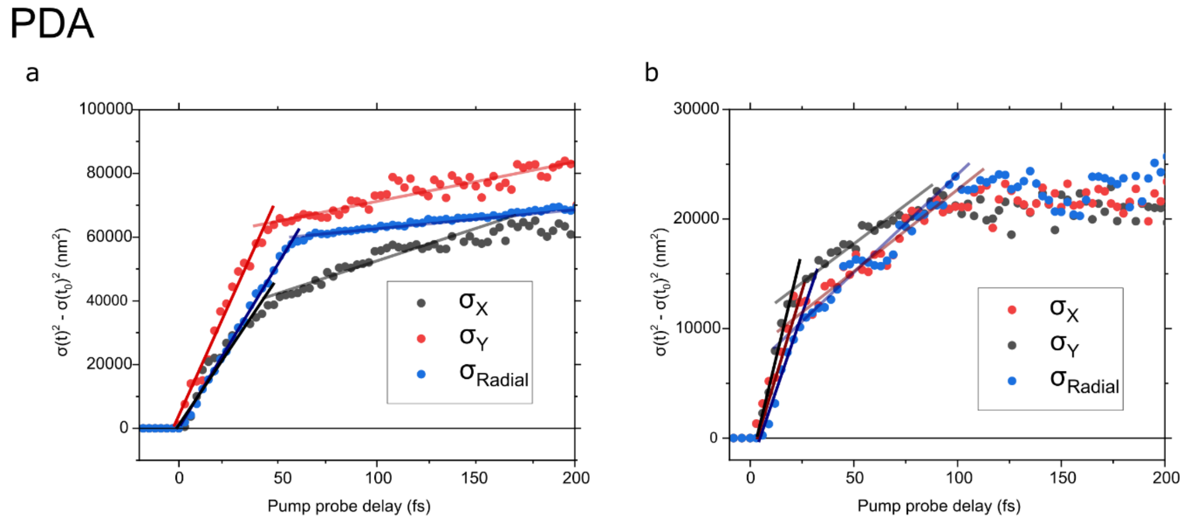

Supplementary Figure 50: a-b. Mean square displacement for the two different sample locations in PDA shown above. There is a good agreement between σ_X and σ_{radial} (value obtained from fitting method detailed above and used in the main text).

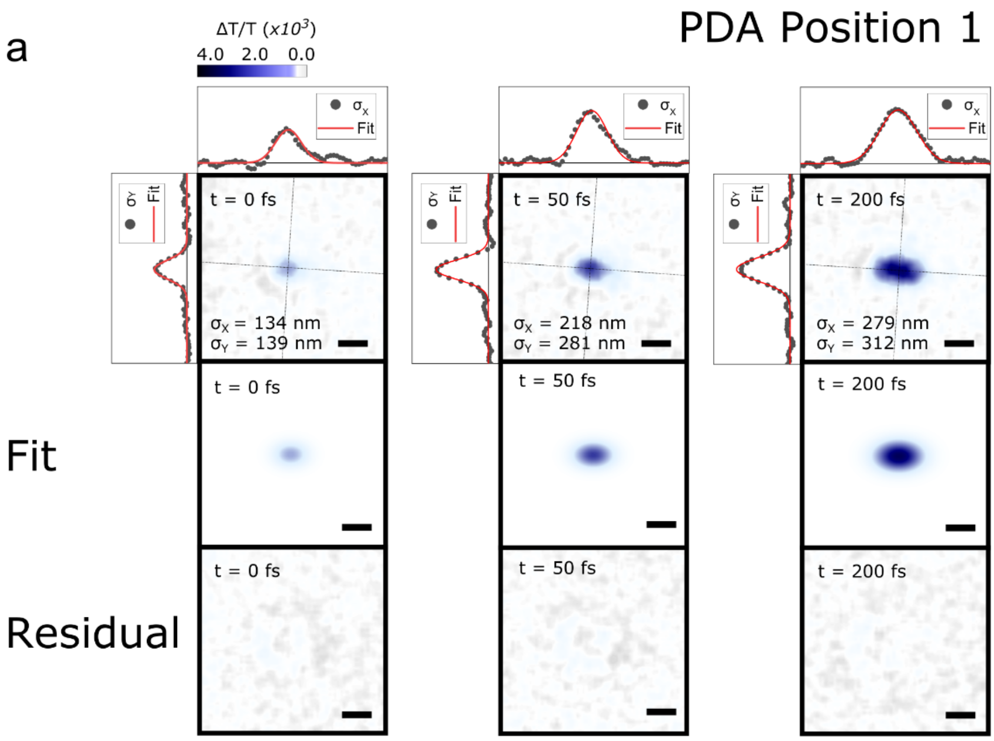

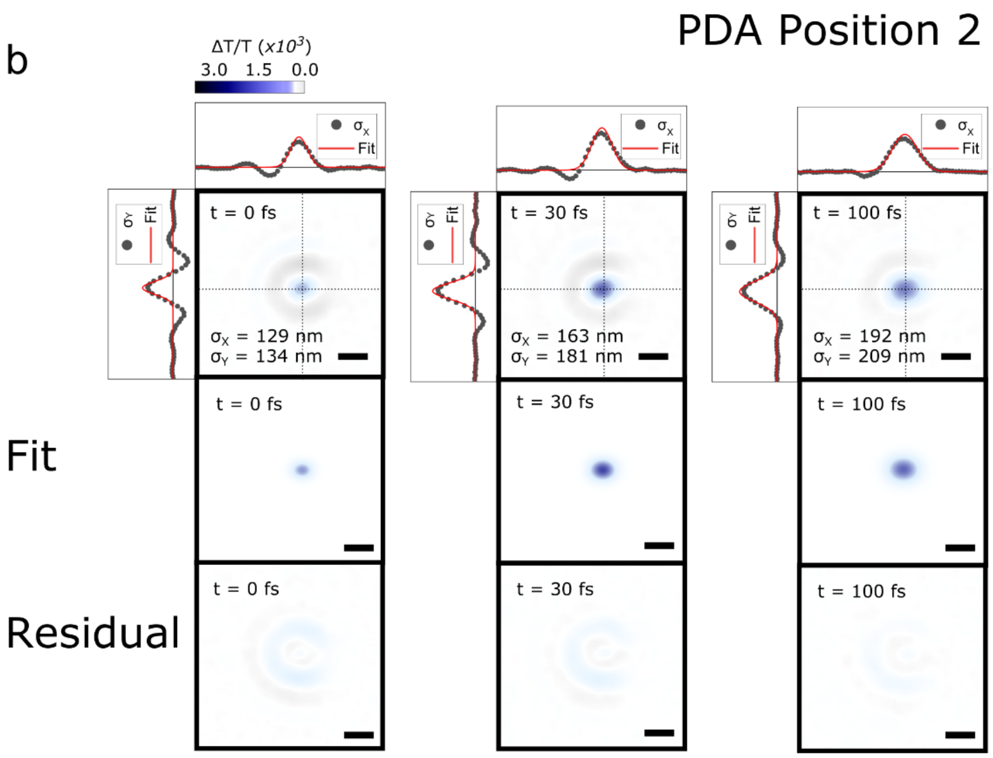

Supplementary Figure 51: a-b. fs-TAM images of PDA at three time delays, overlaid with the principle axes . Side panels show data along the principle axes (black dots) and the corresponding fits

(red line). The fits and residuals are shown in the bottom half; the residuals are dominated by strong Airy disks. The scale bar is 500 nm in all images.

PIC

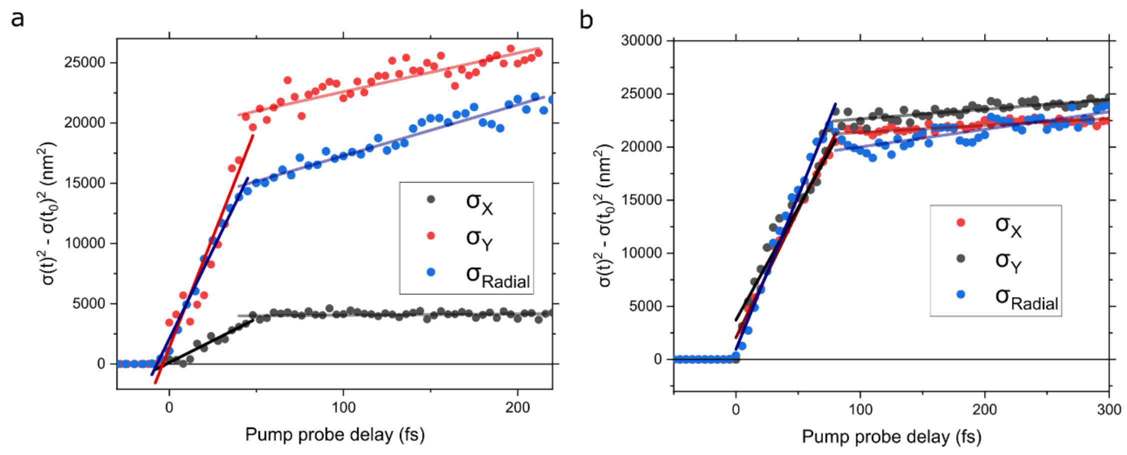

Supplementary Figure 52: a-b. Mean square displacement for the two different sample locations in PIC shown above. The x and y directions also correspond to those in the above. There is a good agreement between σ_X and σ_{radial} (value obtained from fitting method detailed above and used in the main text), with a small degree of overestimation in the case of the 2D Gaussian. When the motion is particular anisotropic displacement in the direction orthogonal to transport is small, in this case σ_Y.

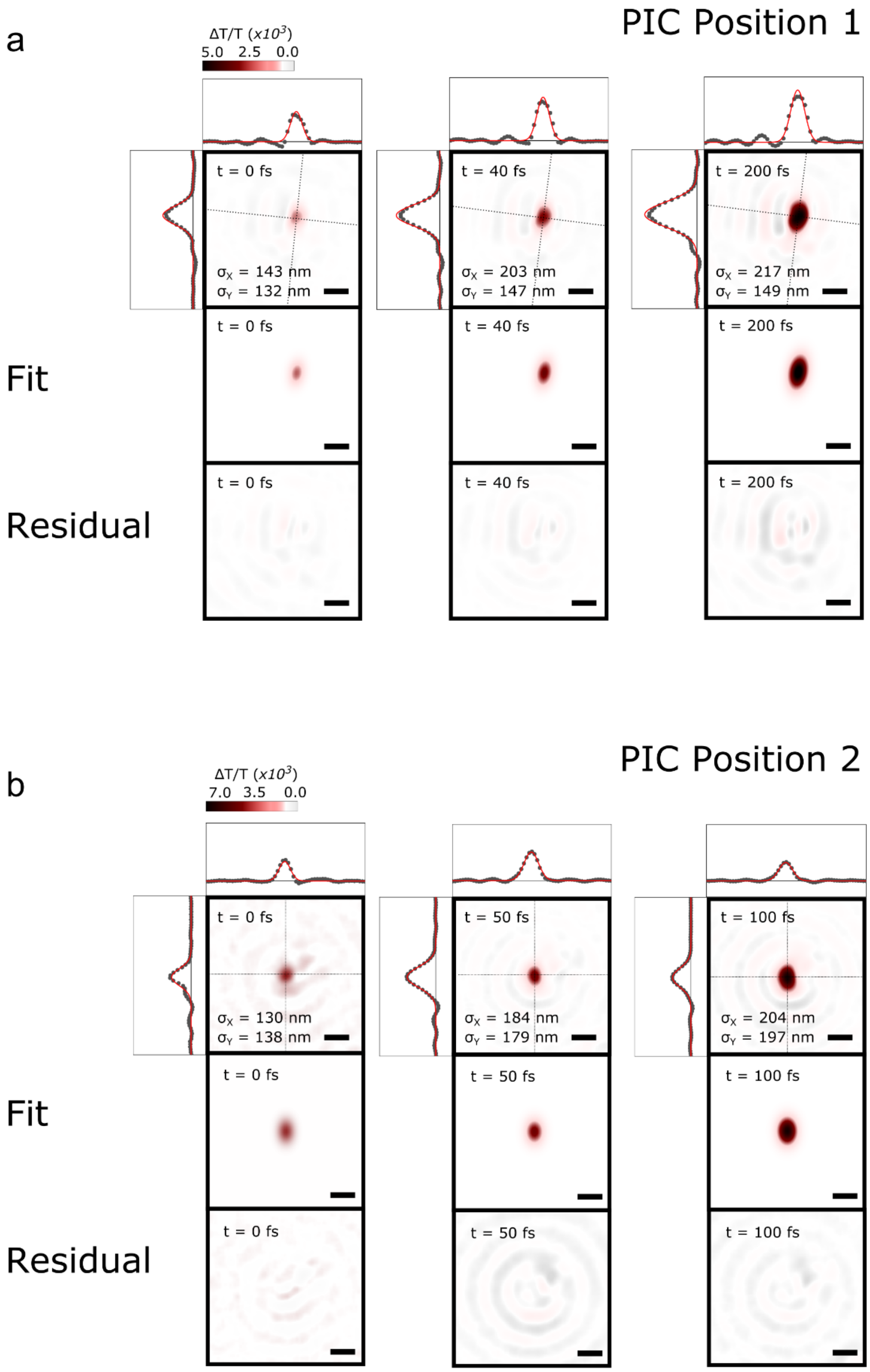

a
PIC Position 1
ΔT/T (x10^3)
5.0 2.5 0.0
t = 0 fs
σX = 143 nm
σY = 132 nm
t = 40 fs
σX = 203 nm
σY = 147 nm
t = 200 fs
σX = 217 nm
σY = 149 nm
Fit
Residual
b
PIC Position 2
ΔT/T (x10^3)
7.0 3.5 0.0
t = 0 fs
σX = 130 nm
σY = 138 nm
t = 50 fs
σX = 184 nm
σY = 179 nm
t = 100 fs
σX = 204 nm
σY = 197 nm
Fit
Residual

Supplementary Figure 53: a-b. fs-TAM images of PIC at three time delays and two different positions. Dotted lines show principle axis of the 2D Gaussian fit to data. Side panels show data along the principle axes (black dots) and the corresponding fits (red line). The scale bar is 500 nm in all images.

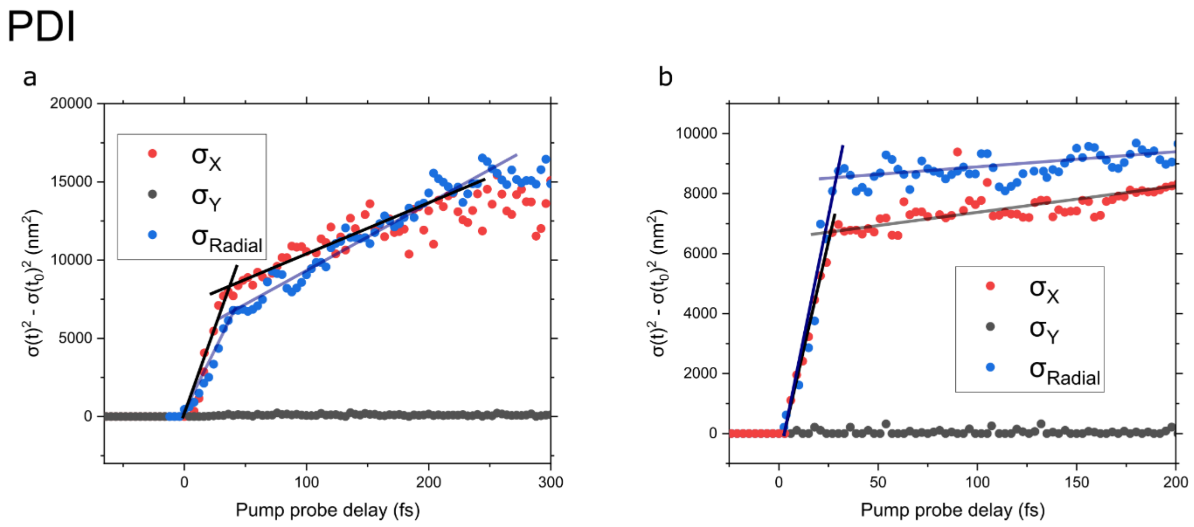

Supplementary Figure 54: a-b. Mean square displacement for the two different sample locations in PDI shown above. There is a good agreement between σ_X and σ_{radial}, with a small degree of underestimation in the case of the 2D Gaussian. When the motion is particularly anisotropic displacement in the direction orthogonal to transport is small.

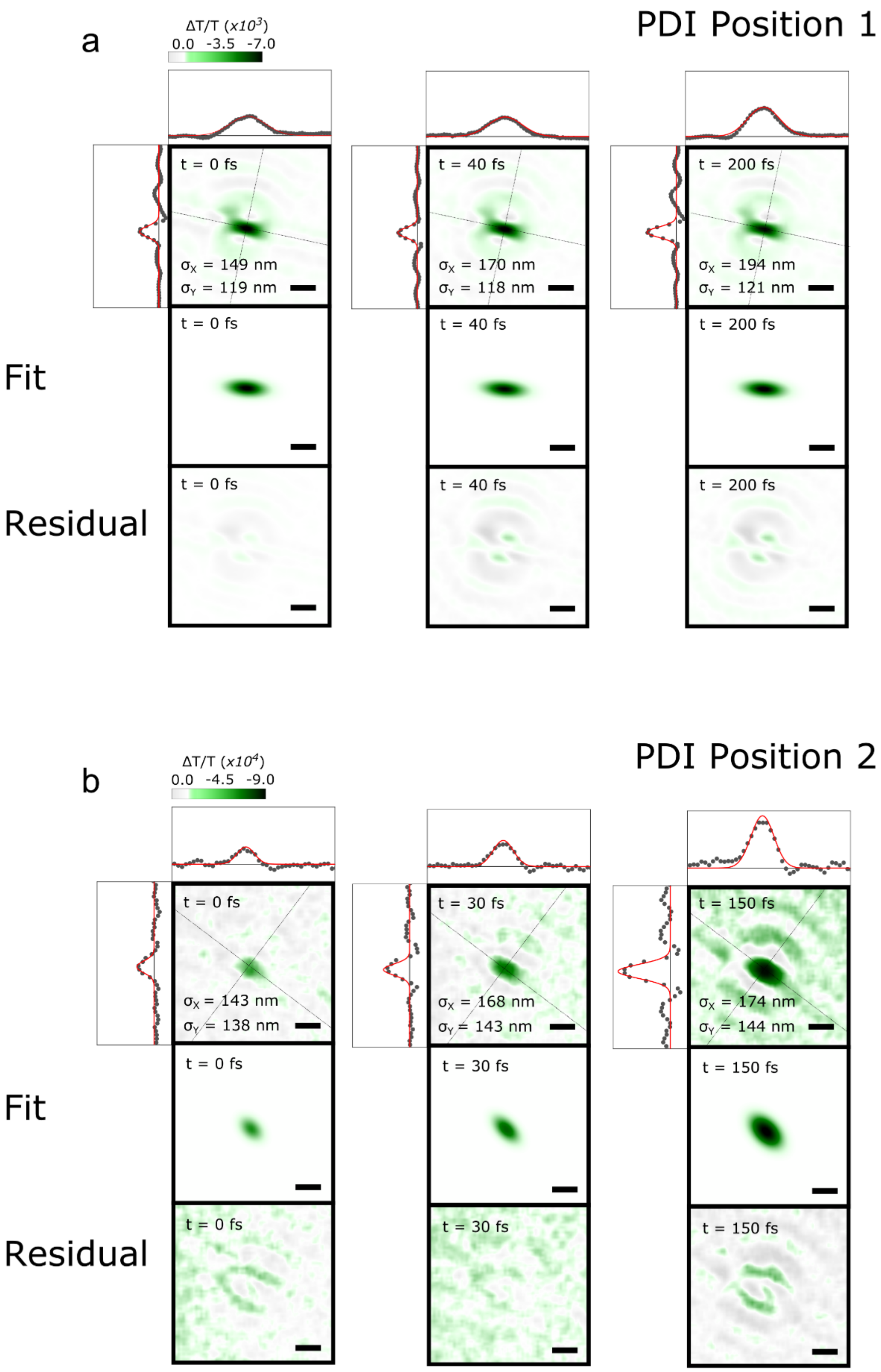

a
ΔT/T (x10^3)
0.0 -3.5 -7.0
PDI Position 1
t = 0 fs
σX = 149 nm
σY = 119 nm
t = 40 fs
σX = 170 nm
σY = 118 nm
t = 200 fs
σX = 194 nm
σY = 121 nm
Fit
Residual
b
ΔT/T (x10^4)
0.0 -4.5 -9.0
PDI Position 2
t = 0 fs
σX = 143 nm
σY = 138 nm
t = 30 fs
σX = 168 nm
σY = 143 nm
t = 150 fs
σX = 174 nm
σY = 144 nm
Fit
Residual

Supplementary Figure 55: a-b. fs-TAM images of PDI at three time delays and two different positions. The scale bar is 500 nm in all images.

Supplementary Note 7: Probe wavelength dependence

For PDA and PIC the spectrally sharp stimulated emission bands represents the only transitions we could probe because of the limited probe wavelength range and the pump wavelength. However, in PDI where we probed the broad PIA feature, we measured the dynamics of a different wavelength (780 nm), in order to check whether the probe wavelength had any effects on the reported signal diffusion. As can be seen from Supplementary Figure 56 there appears to be little dependence on probe wavelength.

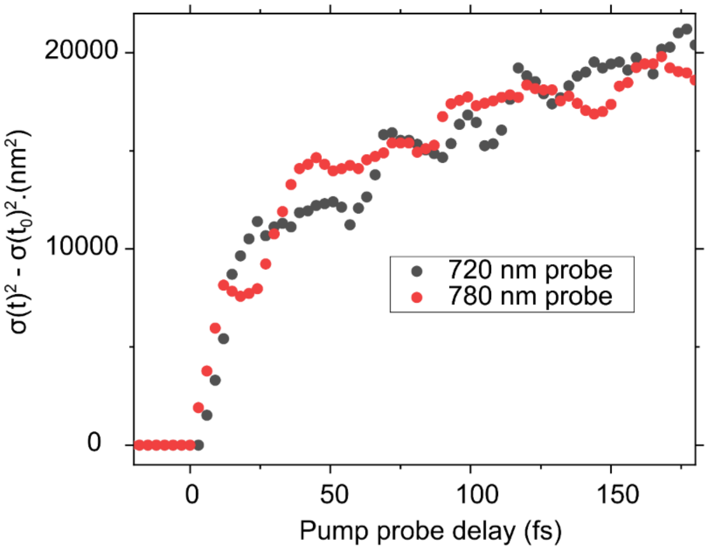

Supplementary Figure 56: Mean square displacement in PDI wires at 720 nm (black) and 780 nm (red) probe wavelengths. No difference can be observed and hence we conclude that within the same band the probe wavelength has little effect on the observed dynamics.

Femtosecond pump-probe spectra of PDA

PDA is an archetypal conjugated polymer which has been extensively studied via time-resolved spectroscopies. In Supplementary Figure 57 we present the pump-probe spectra of PDA; all experiments are carried out with the pump and probe polarized parallel to the long axis of the PDA chains. There are three distinct species in the spectra whose decays can all be fitted with a single exponential. Between 650 – 800 nm, we observe a rapid (~320 ± 5 fs) decay of the PDA SE from the $1B_u$ state. The SE consists of a vibrational progression of peaks. Additionally, between 820 – 900 nm there is an energetically broad band that has been previously assigned to the $2A_g$ photo-induced absorption (PIA). Vibronic relaxation from $1B_u$ to $2A_g$ means the growth of the $2A_g$ PIA is delayed with respect to that of the SE; $2A_g$ then decays with an exponential time constant of ~380 ± 5 fs. The results above are fully consistent with previous pump-probe data and state assignments on a range of 'blue' PDA. A prominent

third species between 675 – 750 nm grows from the $1B_u$ and $2A_g$ states (τ_{rise} ~350 ± 10 fs). This state has been recently assigned to arise from a vibrationally 'hot' ground state. TAM measurements were performed at a probe wavelength of 670 nm on the edge of the stimulated emission band of PDA. Discussion of results at other probe wavelengths e.g. within the $2A_g$ band or 'hot' ground state are beyond the scope of this work.

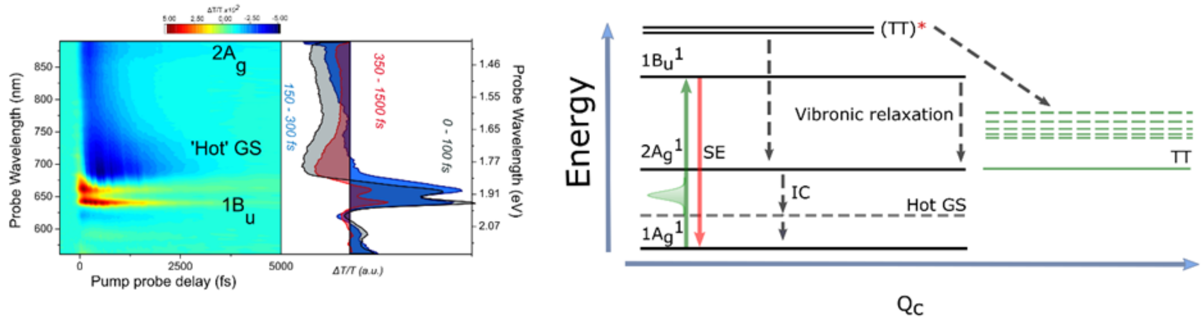

Supplementary Figure 57: Pump-probe spectrum and corresponding spectral cuts at indicated time delays of PDA crystal. The positive (red) regions at 640 nm and 670 nm correspond to stimulated emission from the first vibronic and zero phonon transitions. Additionally, at 720 nm and 870 nm there are two photo-induced absorption features (PIA), which we assign to the vibrationally 'hot' ground state (GS) and the $2A_g$ state respectively. The PIA below 630 nm are not assigned. The right hand figure shows an energy diagram summarizing the electronic states of PDA. The x-axis Q_c represents the reaction coordinate. The pump pulse (green Gaussian) directly populates $1B_u$, which is followed by a rapid (~150 fs in PDA) vibronic relaxation to $2A_g$. $1B_u$ and $2A_g$ then relax back to $1A_g$ via the 'hot' ground state (Hot GS).

Femtosecond pump-probe spectra of PIC

The pump-probe response of PIC thin films has been described extensively in the literature, hence here we only provide a brief description. Thin films were excited with a broadband pump pulse (520 nm centre, ~60 nm FWHM, 10 fs) with the polarization of the white light continuum probe set to 'magic angle' to avoid photoselection effects. Supplementary Figure 58 shows the pump probe spectrum contains two sharp transitions: a positive band at ~583 nm associated with the overlapping ground state bleach (GSB)/stimulated emission (SE) of the nanotubes and a negative photo-induced absorption (PIA) band at 575 nm resulting from S_1-S_2 transitions. The congested spectrum precludes absolute determination of the rate constants for individual relaxation processes but we note the decay appears approximately uniform across the spectrum with a fast component τ = 130 ± 20 fs and a slow component τ = 5.2 ± 0.2 ps. Within the range of excitation densities considered here, $(\Delta T/T)_{max}$ = 0.008, the kinetic dynamics is independent of fluence. In fs-TAM, to achieve the required bandwidth for pump and probe

pulse compression we are limited to probing solely the stimulated emission band at ~600 nm which is strongly overlapped with GSB albeit on the low energy red edge.

For completeness, transient reflectivity measurements were performed on sample locations that showed a splitting in their reflection spectra and that those did not. The pulse configuration used was similar to that detailed in (S1), however the sample was tilted slightly with respect to the incident beams in order to collect the reflected signal shown in Supplementary Figure 58a. A high resolution 2700 lines per mm (550 nm blaze) grating (Andor) was used. The resulting ΔR/R spectrum contains similar features to that obtained in transmission measurements but for regions that do show a splitting the GSB/SE peak around 580 nm is divided into two branches, assigned to an upper and lower polariton respectively. The congested spectrum prevents any splitting within the PIAs from being observed.

In Supplementary Figure 58d we compare the kinetic at an integrated probe wavelength 590 – 610 nm (GSB/SE) for a sample location that does show splitting and one that does not. In the former case the decay and rise of the signal is much slower than where there is no splitting (τ_{rise} = 120 ± 10, τ = 280 ± 20, τ = 5.9 ± 0.3 ps – no splitting; τ_{rise} = 170 ± 10, τ = 375 ± 10, τ = 7.2 ± 0.2 ps – splitting), as was observed in fs-TAM measurements. The origin of this is unknown and investigation beyond the scope of this work, but for example may represent some relaxation time between the upper and lower polariton branches or the lower polariton branch and the exciton reservoir.

Similar experiments were performed on PDA thin films. However, the smaller splitting precluded observation of split bands in transient reflection experiments within the signal-to-noise limit.

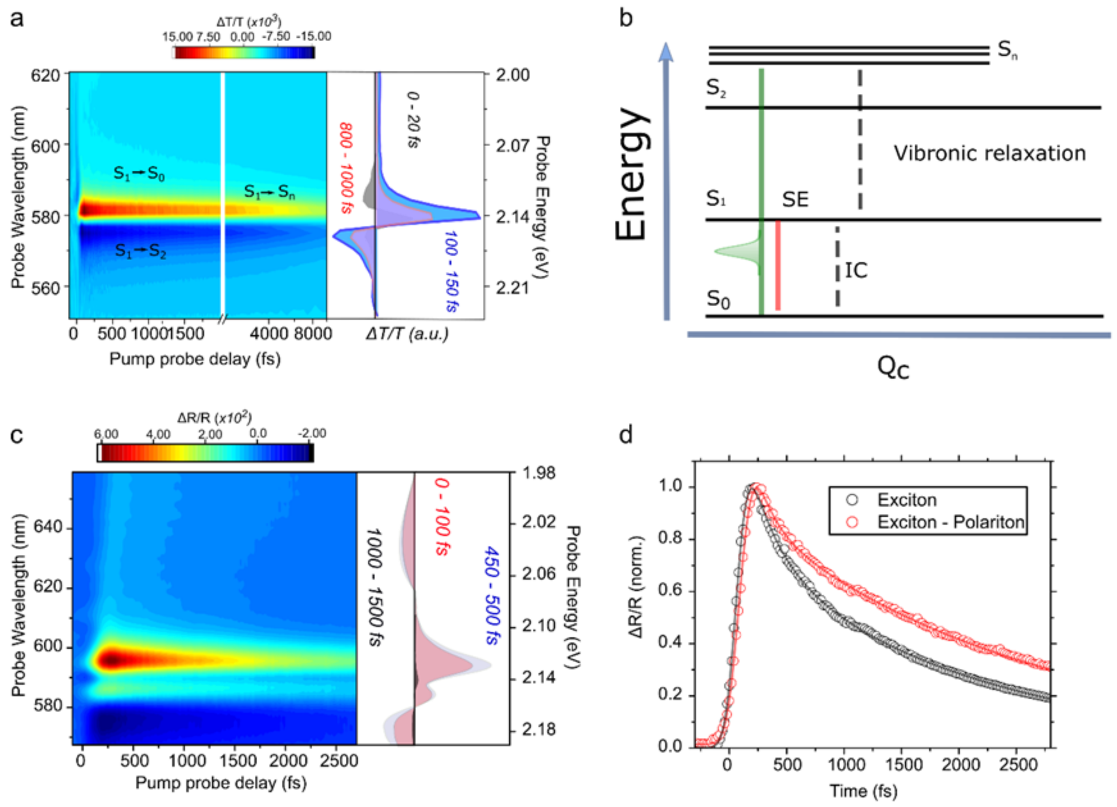

Supplementary Figure 58: a. Pump probe spectrum and corresponding spectral cuts at indicated time delays of PIC thin film. The positive (red) regions correspond to the highly overlapped ground state bleach (GSB)/stimulated emission (SE), with a sharp photo-induced absorption band centred around 577 nm. **b.** The right hand figure shows a simplified cartoon of the energy levels in the system. Excitation occurs into a S_n manifold from which there is ultrafast vibronic relaxation to S_1. Stimulated emission, which is slightly red shifted from the GSB, occurs from this state. The PIA corresponds to transitions between $S_2 - S_1$. **c.** Transient reflection spectrum of PIC in a sample location that shows a splitting of the main J-band in steady state reflectivity. The GSB/SE transition is split into bands assigned to the upper (UP) and lower polariton (LP). **d.** The decays of the UP and LP are bi-exponential with the fast component having a higher amplitude. The faster component of UP decays significantly shorter than the LP; τ_{UP} = 220 ± 10 fs versus τ_{LP} = 375 ± 10 fs.

Femtosecond pump-probe spectra of PDI nanobelts

Extensive description and analysis of the pump probe spectra of PDI nanobelts is beyond the scope of this work, hence we limit the following to a brief description of the spectra. Thin films consisting of large clusters of PDI nanowires were excited with a broadband pump pulse (530 nm centre, ~55 nm FWHM, 12 fs). Within the probe range considered the spectrum shows a broad positive feature 550 nm

– 650 nm which is well overlapped with emission of the nanowires and hence assign to stimulated emission (SE). Between 650 nm – 800 nm there is a broad negative photo-induced absorption band which we tentatively ascribe to an S_1-S_2 transition. At later time delays there may be relaxation into excimer sub-levels, however based on previous reports this would be expected to be on nanosecond time scales. Furthermore, although there have been several reports of intersystem crossing and singlet fission in perylene dimiide based semiconductors, we believe on the timescales considered here the effects of crossover into a triplet manifold are negligible. Consequently, we feel confident that in fs-TAM measurements that at 720 nm we are probing a singlet exciton transition.

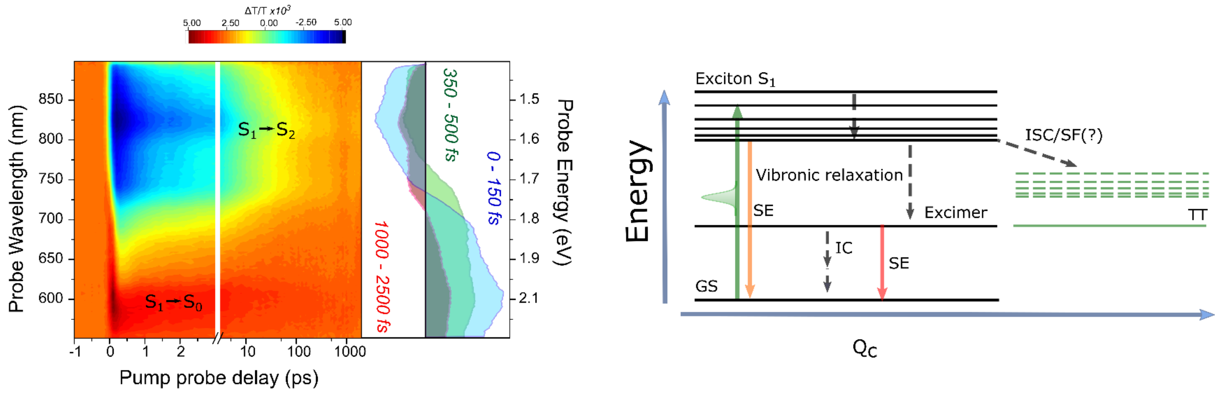

Supplementary Figure 59: Pump probe spectrum and corresponding spectral cuts at indicated time delays of PDI nanobelts. The positive (red) regions correspond to stimulated emission (SE) and the broad negative region (probed in TAM) is the S_1-S_2 photo-induced absorption band (PIA). On the right is shown a cartoon of the corresponding energy levels (Q_c – indicates reaction coordinate). For the timescales studied it is assumed there is no crossing to the triplet manifold. Furthermore, any vibronic relaxation or internal conversion (IC) to low energy excimer states is presumed to be relatively inefficient.

Kinetics of fs-TAM signal

As discussed in the main text, the fs-TAM response shows a large variation depending on the spot on the sample. This is particularly evident in the kinetics of response (averaged across the entire spatial signal) as shown in Supplementary Figure 60. For example, in PDA there is sometimes a slow ~70 fs growth to the signal (a and c) where in other positions the signal rise is instrument response limited (b). In all cases there is an initial fast ~250 fs decay component to the SE but for several positions an additional 1.2 – 1.8 ps lifetime is required to fit the decay. As seen in Supplementary Figure 60c there is also the potential for small coherent artefact contributions to the signals at some locations in the sample.

In PIC and PDI the response is slightly more homogenous. For PIC the signal shows a slow ~180 fs rise, followed by a bi-exponential decay (τ_1~350 fs and τ_2~7.6 ps). In PDI the rise of the signal is often instantaneous with an overall multi-exponential decay.

Understanding the origin of this behaviour goes beyond the scope of the present work. However, we highlight in all three samples that the spatial growth is independent of the signal strength, especially in the region of ultrafast expansion. Indeed, the signal expands outwards throughout the initial rise and the subsequent decay, proving that the observations described do not arise from an incorrect assignment of t_0.

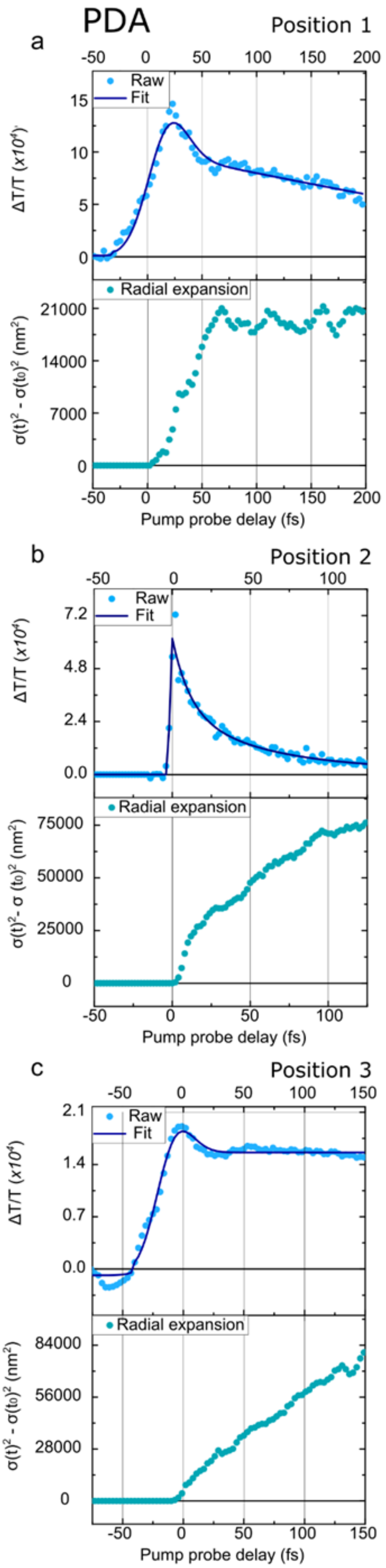

a
PDA
Position 1
Raw
Fit
ΔT/T (x10⁴)
Radial expansion
σ(t)² - σ(t₀)² (nm²)
Pump probe delay (fs)
b
Position 2
Raw
Fit
ΔT/T (x10⁴)
Radial expansion
σ(t)²- σ (t₀)² (nm²)
Pump probe delay (fs)
c
Position 3
Raw
Fit
ΔT/T (x10⁴)
Radial expansion
σ(t)² - σ(t₀)² (nm²)
Pump probe delay (fs)

Supplementary Figure 60: a-c. Kinetics (dots - raw data; solid lines - fit) and mean square displacement traces at three different spots in PDA. The $\sigma(t)^2 - \sigma(t_0)^2$ trace consistently shows a biphasic response but the kinetics vary significantly between different sample locations. In some cases, (**c**), a small coherent artefact can be observed before time zero. The vertical grid lines are added to allow for comparison between the two plots.

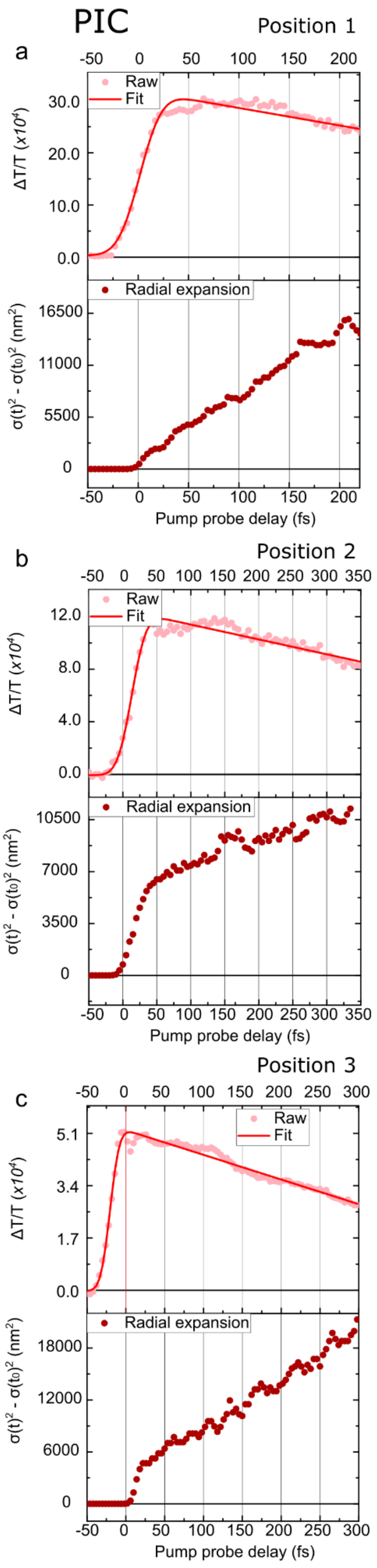
PIC
Position 1
a
Raw
Fit
ΔT/T (x10⁴)
Radial expansion
σ(t)² - σ(t₀)² (nm²)
Pump probe delay (fs)
Position 2
b
Raw
Fit
ΔT/T (x10⁴)
Radial expansion
σ(t)² - σ(t₀)² (nm²)
Pump probe delay (fs)
Position 3
c
Raw
Fit
ΔT/T (x10⁴)
Radial expansion
σ(t)² - σ(t₀)² (nm²)
Pump probe delay (fs)

Supplementary Figure 61: a-c. Kinetics (dots - raw data; solid lines - fit) and mean square displacement traces at three different spots in PIC. The $\sigma(t)^2 - \sigma(t_0)^2$ trace consistently shows a biphasic response and in this sample the kinetics are relatively consistent between sample locations.

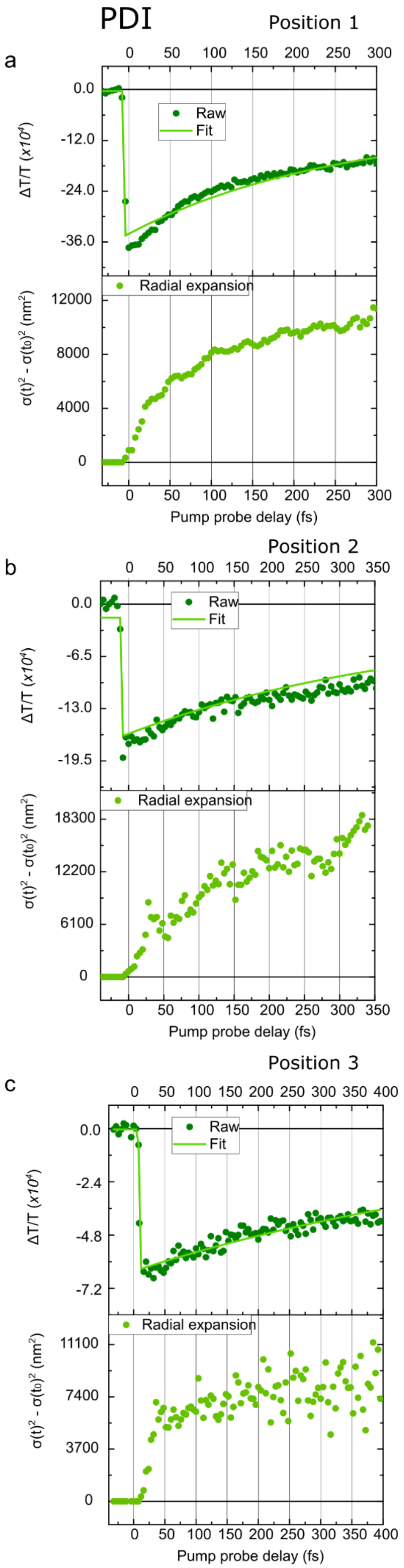
PDI
Position 1
a
Raw
Fit
ΔT/T (x10⁴)
Radial expansion
σ(t)² - σ(t₀)² (nm²)
Pump probe delay (fs)
Position 2
b
Raw
Fit
ΔT/T (x10⁴)
Radial expansion
σ(t)² - σ(t₀)² (nm²)
Pump probe delay (fs)
Position 3
c
Raw
Fit
ΔT/T (x10⁴)
Radial expansion
σ(t)² - σ(t₀)² (nm²)
Pump probe delay (fs)

Supplementary Figure 62: a-c. Kinetics (dots - raw data; solid lines - fit) and mean square displacement traces at three different spots in PIC. The $\sigma(t)^2 - \sigma(t_0)^2$ trace consistently shows a biphasic response and in this sample the kinetics are relatively consistent between sample locations.

Supplementary Note 8: Modelling coherent transport in fs-TAM data

At long times an initial Gaussian population will follow diffusive dynamics due to multiple scattering events effectively randomising the velocities of the carriers and giving the overall profile the dynamics of a Markovian random walk. It can be shown in this $t >> \tau$ limit that the characteristic diffusion constant in a 1D system appears as,

$$\sigma^2 = \sigma_0^2 + 2Dt \quad (13)$$

It is desirable to have a similar $\sigma(v,t)$ for the coherent transport regime in order to reliably extract the coherent group velocities v from the $\sigma(t)$. This is derived below.

We assume our crystal has the simplest possible 1D band structure - a two band model with parabolic valance and conduction band. We further assume that we are exciting above the bandgap with a delta function like excitation. We assume the crystal has inversion symmetry and the two **k** states we are exciting are at exactly +**k'** and -**k'**. As we are exciting Bloch states of a determinate **k**, they have a well defined group velocity v_g.

Our initial excitation is a Gaussian with a known σ_0. In the range of carrier densities we probe it it is safe to assume that exactly half of them have a velocity $+v_g$ and the other half a velocity $-v_g$. Hence we can

decompose our initial excitation's time evolution as,

$$n(x,t) = \frac{1}{2\sigma\sqrt{2\pi}}\left(e^{\left[-\frac{(x-v_g t)^2}{2\sigma_0^2}\right]} + e^{\left[-\frac{(x+v_g t)^2}{2\sigma_0^2}\right]}\right) \quad (14)$$

Examining the above equation it is conceivable that in the limit $v_g t/\sigma_0 << 1$ the profile still appears Gaussian and we still have an effective σ. The band velocities are typically $< 10^6$ m s^{-1} = 1 nm fs^{-1}, $\sigma_0 \approx 100$ nm for a diffraction limited spot and the times range in which this physics is relevant is ≤ 50 fs at room temperature. For these parameters $v_g t/\sigma_0 < 1$ is well justified. Experimentally it is also well known that in this regime our profiles are Gaussians and we can find $\sigma(t)$. This expression therefore can be Taylor expanded in $v_g t/\sigma_0$ to show that,

$$n(x,t) = 2Ae^{[-\frac{x^2}{2\sigma_0^2}]} - A\left(\frac{vt}{\sigma_0}\right)^2 e^{\left[-\frac{x^2}{2\sigma_0^2}\right]} + A\left(\frac{vt}{\sigma_0}\right)^2 e^{\left[-\frac{x^2}{2\sigma_0^2}\right]} \quad (15)$$

Taking note of initially assumed Gaussian excitation profile and equation 13, we can then derive the expression,

$$\sigma(v,t) = \sigma_0 + \frac{v^2t^2}{2\sigma_0} \quad (16)$$

We can add in scattering by hand as an exponential pre-factor the velocity term and write,

$$\sigma(v,t) = \sigma_0 + e^{\left(\frac{-t}{\tau}\right)}\left(\frac{v^2t^2}{2\sigma_0}\right) \quad (17)$$

The above equation however only reproduces the data accurately in the coherent sub-50 fs time regime as shown in Supplementary Figure 63 and discussed in the main text. The scattering times for the transport are shown in Supplementary Figure 64.

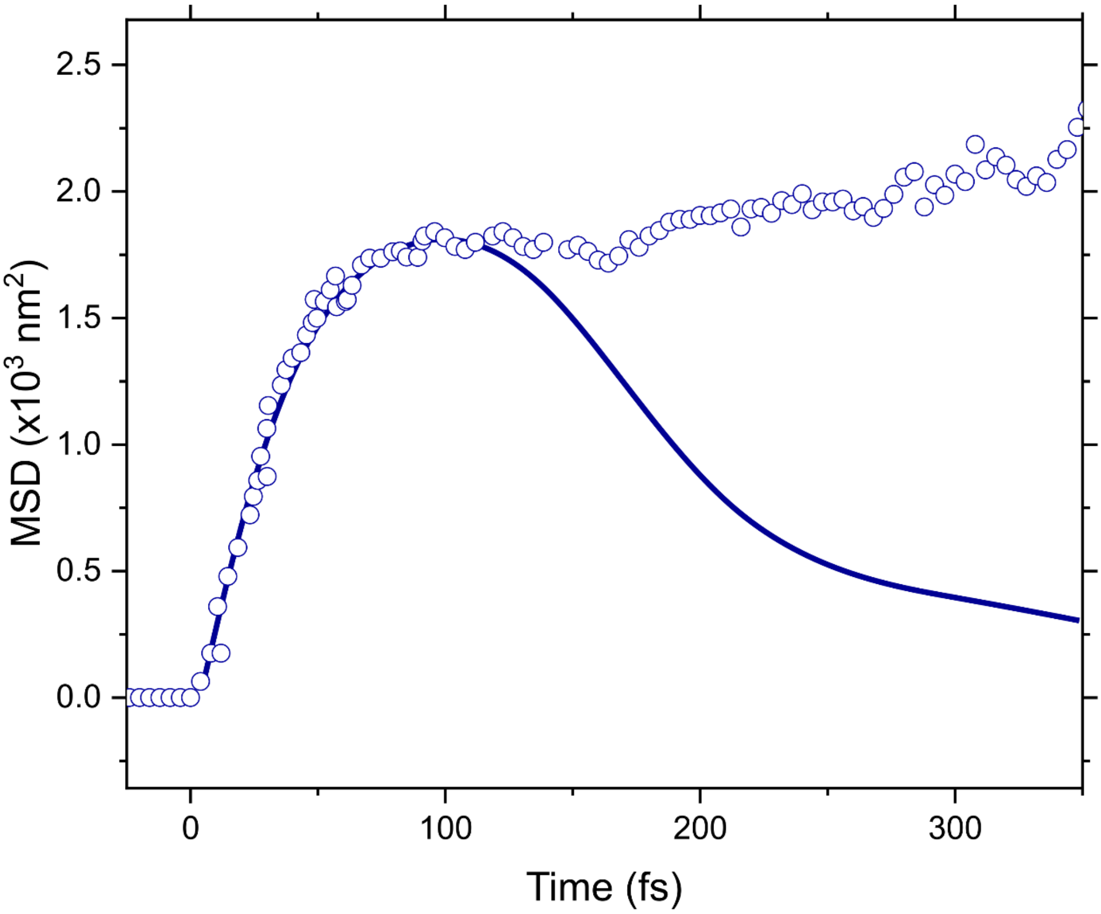

Supplementary Figure 63: Typical MSD trace (circles) for blue PDA with equations detailed above fit (solid line). The equation fits well in the early (sub-50 fs time regime), but deviates at later times in line with a switch from coherent to diffusive/hopping type energy transport.

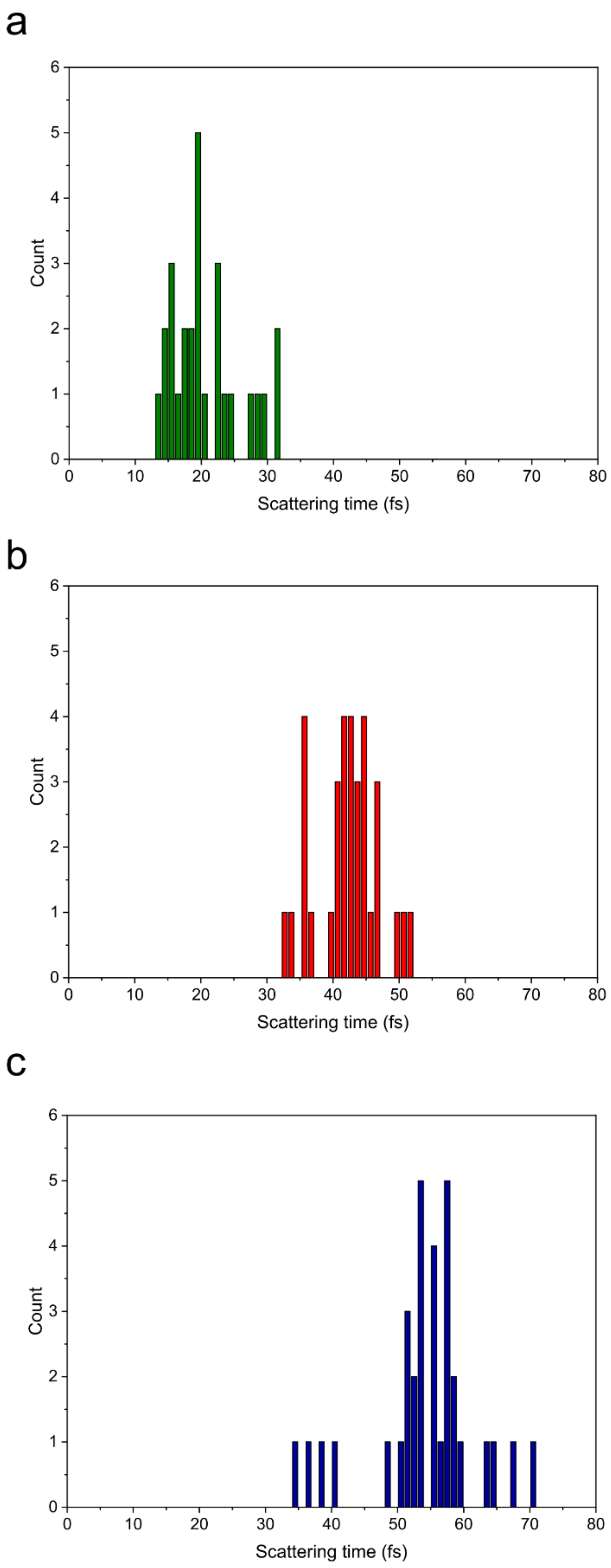

Supplementary Figure 64: a-c. Histogram of scattering times as derived from equations above.

Supplementary Note 9: Modelling annihilation in fs-TAM data

In order to unravel the relative contributions of diffusion and population decay in the observed TAM spatial and temporal dynamics, we attempted to fit the following second-order differential equation to our fs-TAM data:

$$\frac{dp(x,y,t)}{dt} = D\,\nabla^2 p(x,y,t) - \lambda_1 p(x,y,t) - \lambda_2\, p(x,y,t)^2 \quad (18)$$

where $p(x,y,t)$ is the population (or ΔT/T signal strength) at time t, and D is the diffusion coefficient, λ_1 is the one-excitonic decay rate and λ_2 is associated with the rate of bi-excitonic annihilation. The raw data was smoothed using a bilateral filter based on a Gaussian weighting. D was fixed using the value obtained from experiments in R_2. Background offset was corrected, and a plane was subtracted from each image to ensure the background is flat. The image with maximum signal strength was then used as the initial condition for fitting with the Dirichlet boundary condition $p(\pm\infty, \pm\infty, t) = 0$. Strictly speaking, this boundary condition is only valid when the pump spot size (310 nm FWHM) is much smaller than the sample dimensions (at least 1 micron) and hence the exciton population does not 'feel' the crystal boundary as they diffuse. However, this is the best approximation we have, since detailed morphological studies on our sample is beyond the scope of this work. The fit converges as residual sum of squares is minimized. As illustrated in Supplementary Figure 65, the model produces a good fit to our fs-TAM data with an outstanding goodness of fit ($R^2 > 0.95$). The graphs below summarise the fitted annihilation coefficients for each data set of PDA, PIC and PDI.

The magnitude of the annihilation coefficient is 1-3 orders of magnitude below the diffusion coefficient. As a result, we suggest that annihilation does not play a significant role in our fs-TAM experiments. This further demonstrates that the expansion in our signal is a result of carrier transport, not an 'artefact' due to annihilation effects at the centre of the pump spot. Indeed, we would expect if there was significant annihilation occurring that a collapse of the centre Gaussian would be observed. We note that in the fs-TAM kinetics (see Supplementary Figure 66) no annihilation effects are again observed in the fluence regime explored here, suggesting that in general annihilation effects are small.

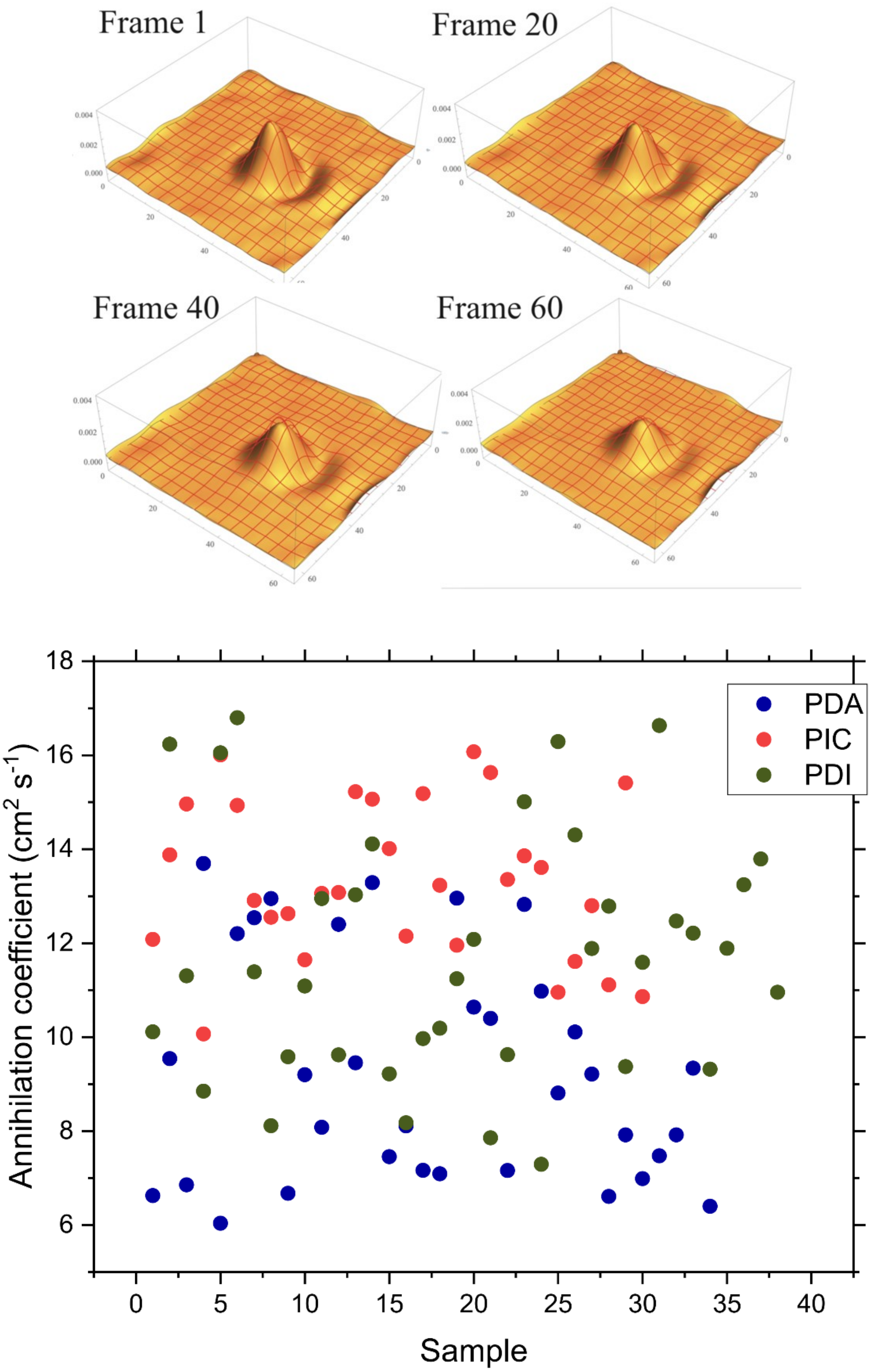

Supplementary Figure 65 (Top) The red mesh corresponds to experimental data and the filled yellow surface is the model fit. We find a good fit between the model and the measured data, with an R-squared value in excess of 0.95. (Bottom) Annihilation coefficient across all sample locations measured in PDA, PIC and PDI. In all cases the annihilation coefficient is between 5 – 20 cm^2 s^{-1} suggesting annihilation is not significant in the materials.

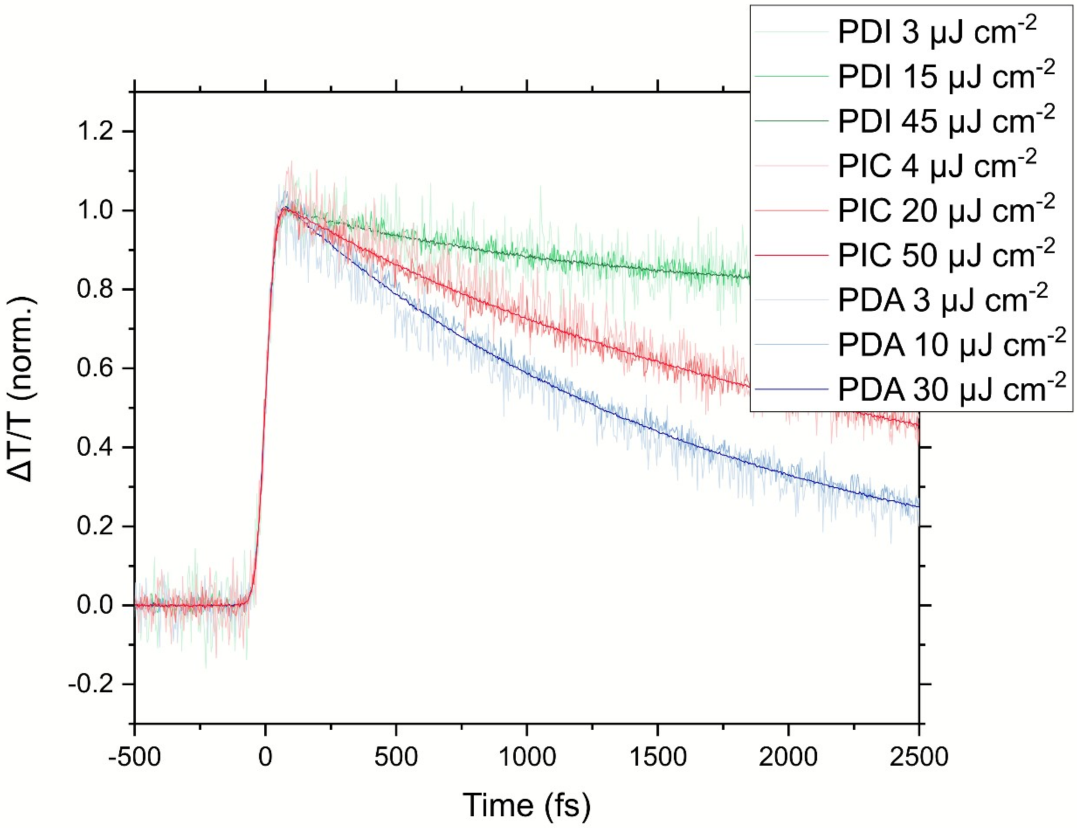

Supplementary Figure 66: Fluence dependence of pump probe kinetics for PDI, PIC and PDA taken at probe wavelengths in fs-TAM. The kinetics are normalised and fluence indicated in the legend.

Pump fluence dependence

For all three materials the effect of varying the fluence of the pump pulse was investigated. The mean square displacement traces in Supplementary Figure 67 show that the spatial dynamics of PDA, PIC and PDI have little dependence on the pump intensity. This is the case for both R_1 and R_2. In the former, this is expected because exciton-polaritons are formed from interaction of the optical dipoles with the vacuum electromagnetic field. Consequently, the polaritonic states are present and accessible even in the absence of excitation. In the low-excitation-density regime, these states and the transport they mediate should be independent of photon flux. In R_2 this is likely the case because we are in the low fluence regime of excitation where thermal and annihilation effects do not play a significant role. The independence of the diffusion coefficient in R_2 suggests that exciton-exciton annihilation effects play a negligible role in the observed behaviour. The differences in the absolute distance travelled are small do not follow a systematic trend and instigation of their origin is beyond the scope of the work.

Repeating the fluence series at 5 separate sample locations in addition to those detailed in the main text allows estimation of the fluence dependence of the mean transport velocity (R_1) and diffusion coefficient (R_2). As shown in Supplementary Figure 68, there is only small variation in the values obtained.

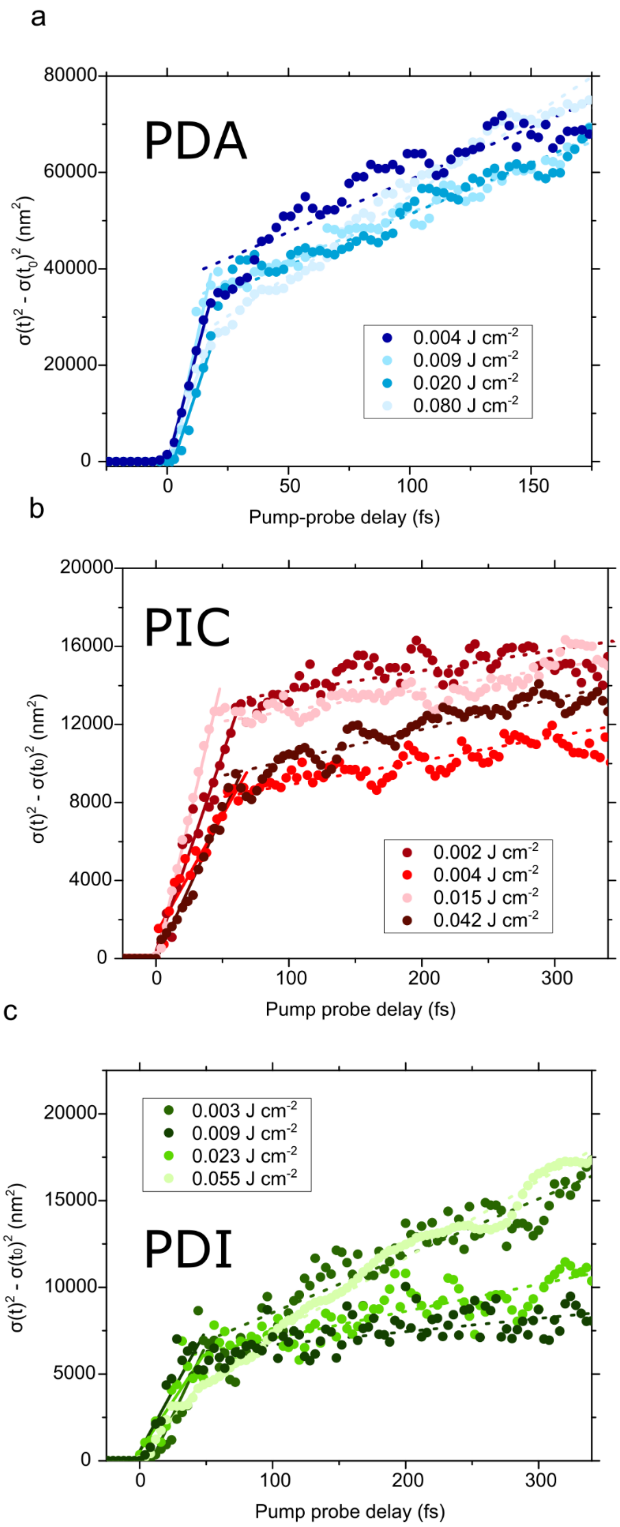

Supplementary Figure 67: Mean square displacement as a function of time at different pump fluences for **a.** PDA, **b.** PIC and **c.** PDI . The straight lines indicate a fit to the data in R_1 (solid) and R_2 (dashed). The absolute distance travelled varies to a small degree between the fluences but there appears to be no systematic trend and the diffusion coefficient (gradient of straight lines) is independent of the pump fluence.

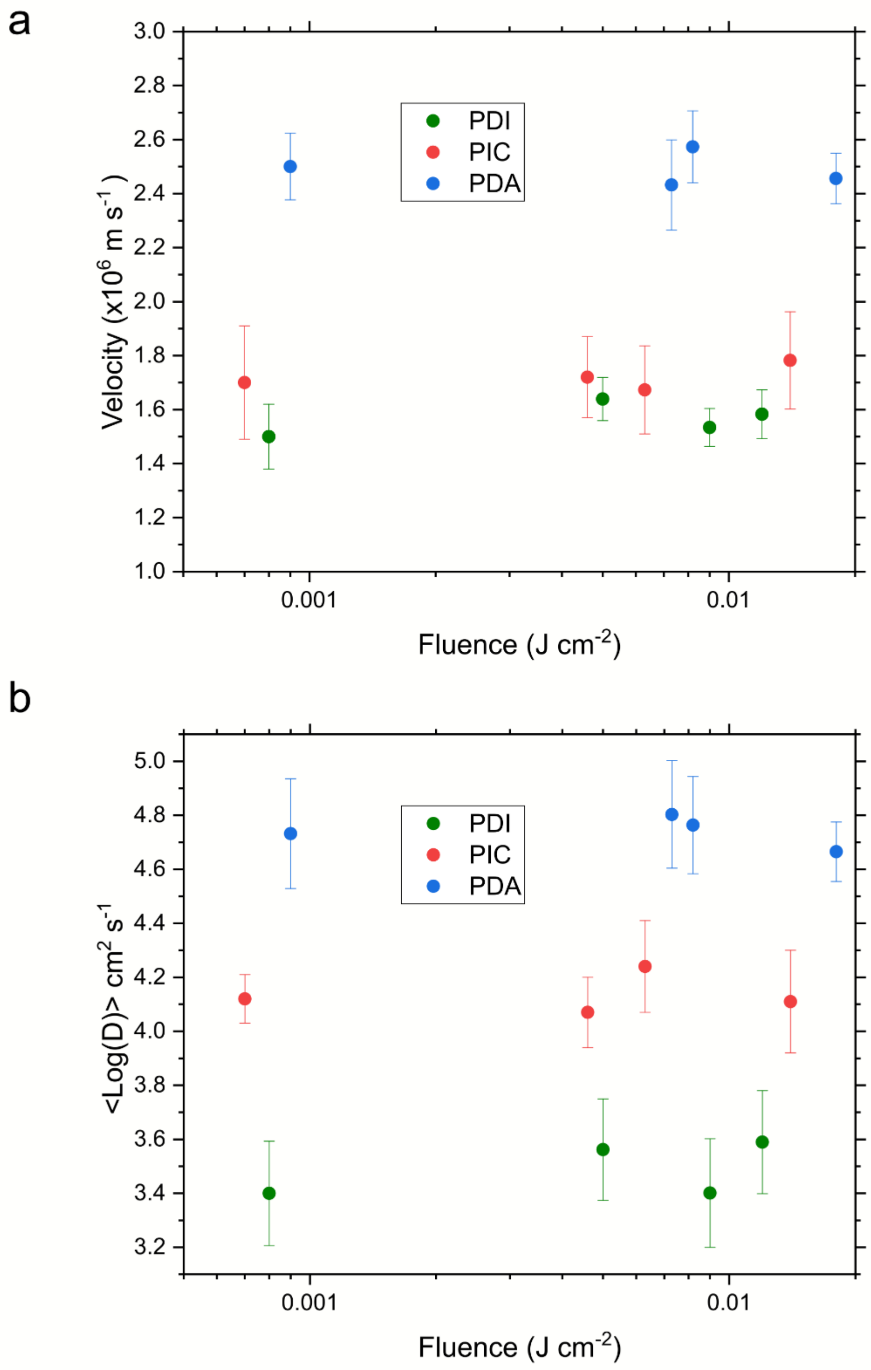

Supplementary Figure 68: a-b. Mean velocity and logarithm of the diffusion coefficient (D) for PDA, PDI and PIC at different laser fluences. At each fluence a minimum of at least 5 different mean square displacement traces are used to obtain the average diffusion coefficient. In general the velocity and D appear to be independent of fluence.

Supplementary Note 10: Correlation between diffusion length and decay time and error bars on MSD

Correlation of the diffusion length with morphology is highly challenging in the systems studied because, as shown in, e.g. Supplementary Note 2, disorder in these materials likely stem from nanoscopic molecular ordering. Information on this cannot be obtained from visible light microscopy techniques. The variation in the diffusion lengths observed hence likely then stems from intermolecular site-to-site couplings which cannot be experimentally obtained. In Supplementary Figure 69, we plot a correlation of the electronic lifetime versus diffusion length, as the reviewer suggests. There appears to be no correlation between these quantities, hence showing electronic lifetimes in this case are not sufficient in understanding diffusion behaviours in these materials.

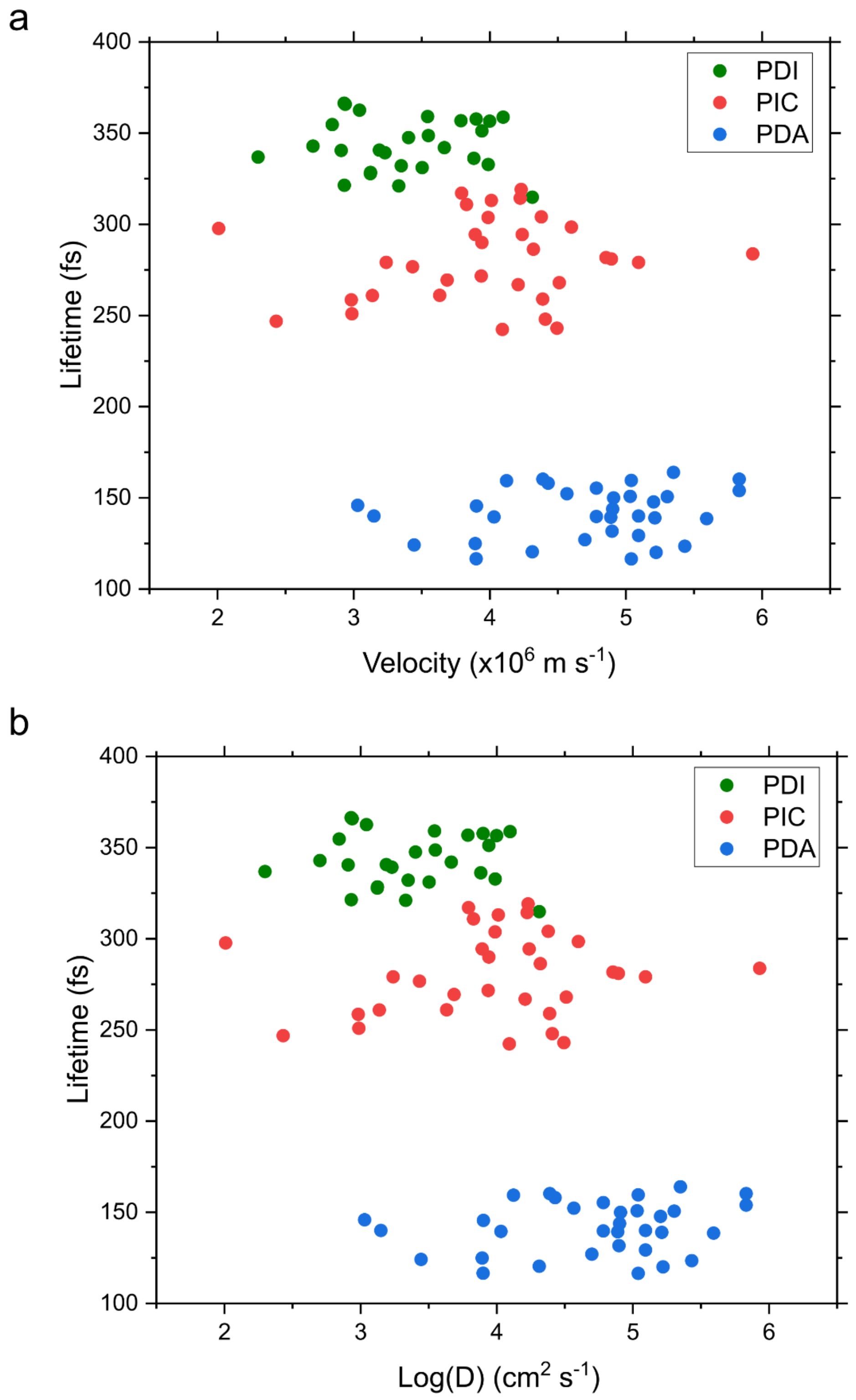

Supplementary Figure 69: a-b. Plot of decay time versus velocity and Log(D) (diffusion coefficient) for R_1 and R_2 regions. There appears to be no correlation between the decay time of the signal and either velocity or diffusion coefficient.

In Figure 4a-c of the main text we do not show error bars as they are sufficiently small and cannot be observed above the data. In Supplementary Figure 70 we show the error bars using the error analysis derived by Wan *et al.* [29]. The error on the point spread function to include noise; the values remain small. The error in Supplementary Figure 71 (bottom) is derived from $\Delta\sigma_{carrier,t}^2 = \sigma_{carrier,t}^2 - \sigma_{carrier,0}^2 = \sigma_{I,t}^2 - \sigma_{I,0}^2$. An error analysis for the combined uncertainty of measurement for the evolution in carrier distribution (Δ(Δσcarrier,t)) can then be calculated as $\Delta(\Delta\sigma_{carrier,t}) = \sqrt{}(\sigma_{I,t}\Delta\sigma_{I,t} + \sigma_{I,0}\Delta\sigma_{I,0})/(\Delta\sigma_{carrier,t})$.

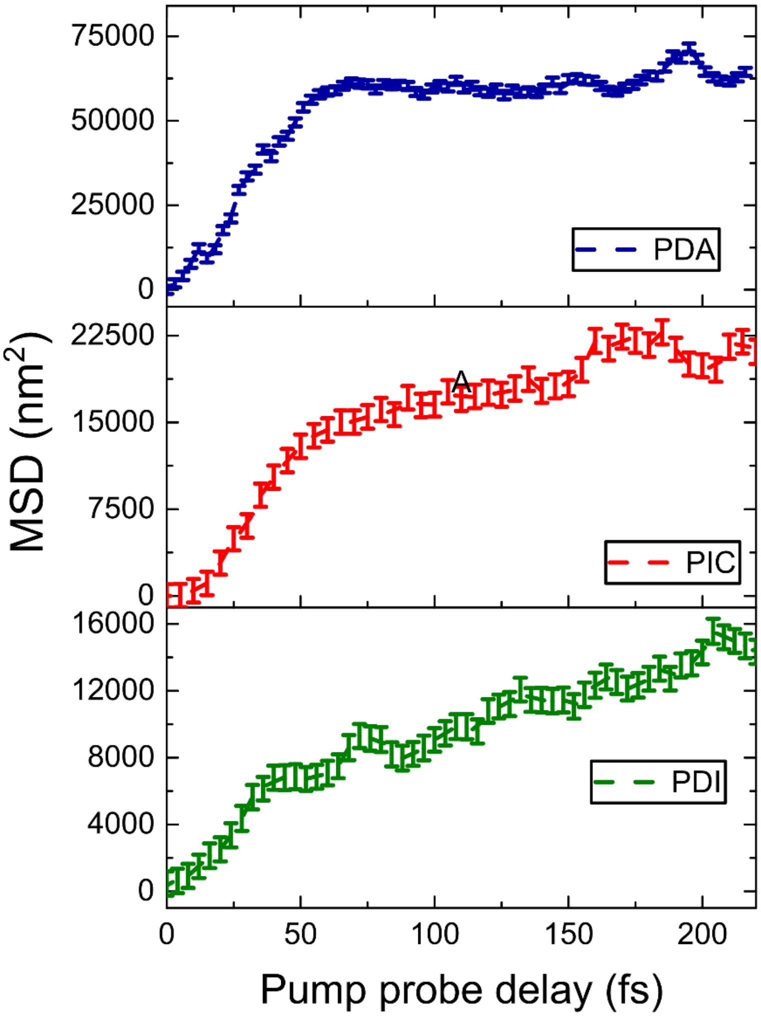

Supplementary Figure 70: Error bars on mean square displacement as derived using the equations in Wan *et al.*[29].

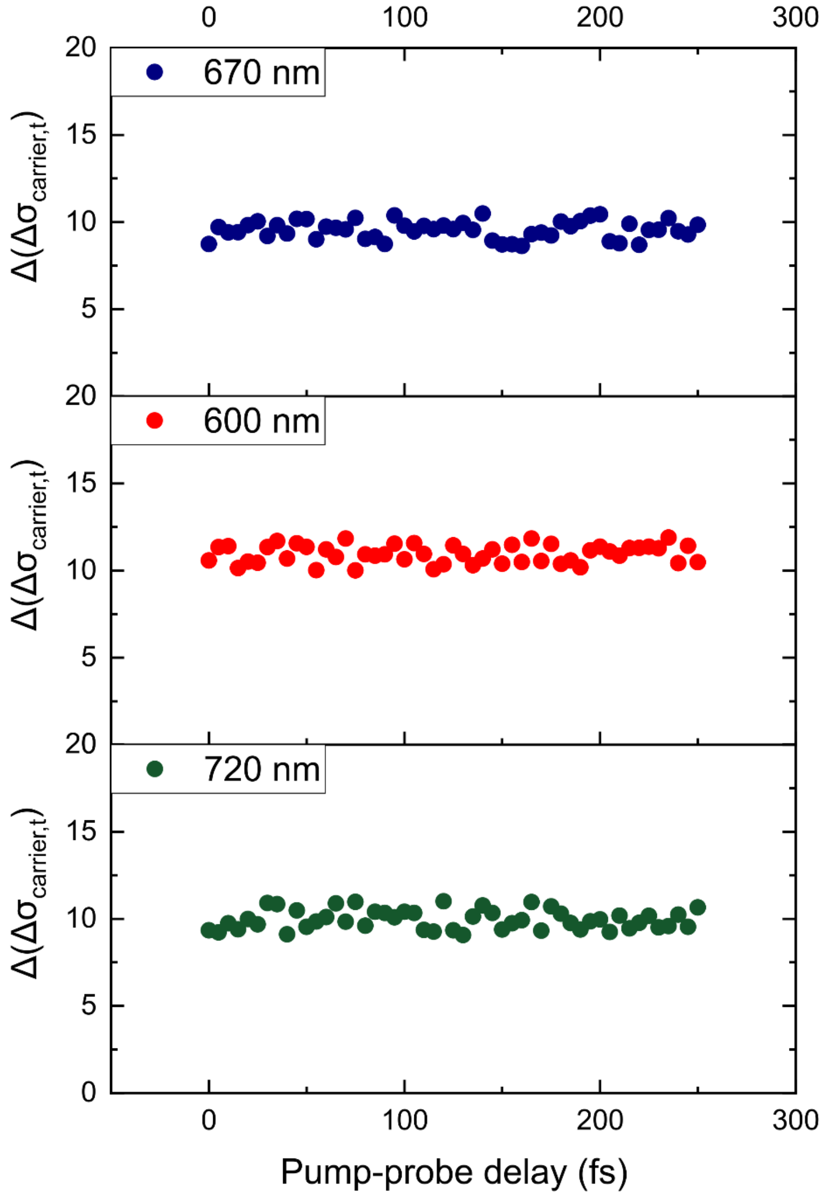

Supplementary Figure 71: (Top) Error bars on mean square displacement as derived using the equations in Wan *et al.*[29]. (Bottom) Combined measurement precision of the measurement system obtained at 670, 600 and 720 nm, respectively.

In the main text of the manuscript the transport observed is described by a biphasic response, with the initial fast and the later slower increases in MSD fit with two separate straight lines. The physical reasoning behind this is that exciton-polaritons are formed initially following photoexcitation, which then de-cohere into more excitonic like transport. Alternative equations such as a power law or stretched exponential can also be used to fit the data as shown in Figure SI78. The power law fit is of the form MSD = Dt^n. We obtain fit parameters of D = 4300 cm^2 s^{-1} and n values of ~0.6. The n value suggests the transport is sub-diffusive but is completely at odds with such a large diffusion coefficient. We hence suggest this model to be unphysical. A stretched exponential model (Supplementary Figure 72b) does not fit the MSD well hence we do not explore this fit further.

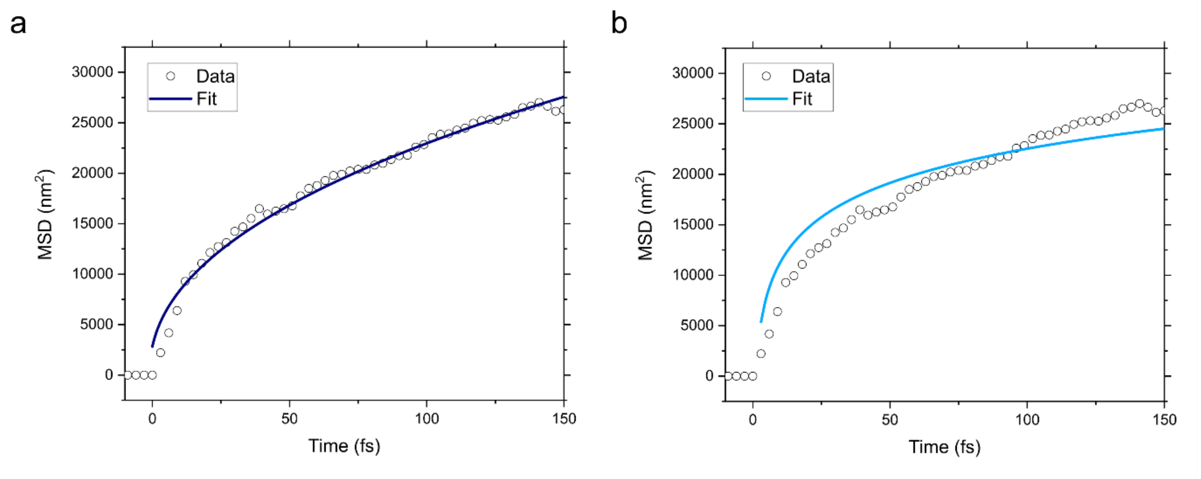

Supplementary Figure 72: Power law and stretched exponential fits to a typical mean square displacement trace for PDA: a. Power law fit (solid line – fit; circles – data). **b.** Stretched exponential fit.

The R_1 region observed and described in the main text arises from the ballistic transport of polaritons. An alternative approach to that taken in Supplementary Note 8 could be to fit the mean free path trace with a quadratic dependence in time i.e. MSD = Dt^2. In Supplementary Figure 73 we show a quadratic fit to the first 20 – 25 fs of transport. Although this equation fits the data we note that the behaviour is close to the instrument response and hence do not use it. Furthermore, the approach taken in Supplementary Note 8 gives a physically motivated scattering time and velocity (a better parameter than the diffusion coefficient which relates to strictly coherent transport). As a result, we do not use this approach.

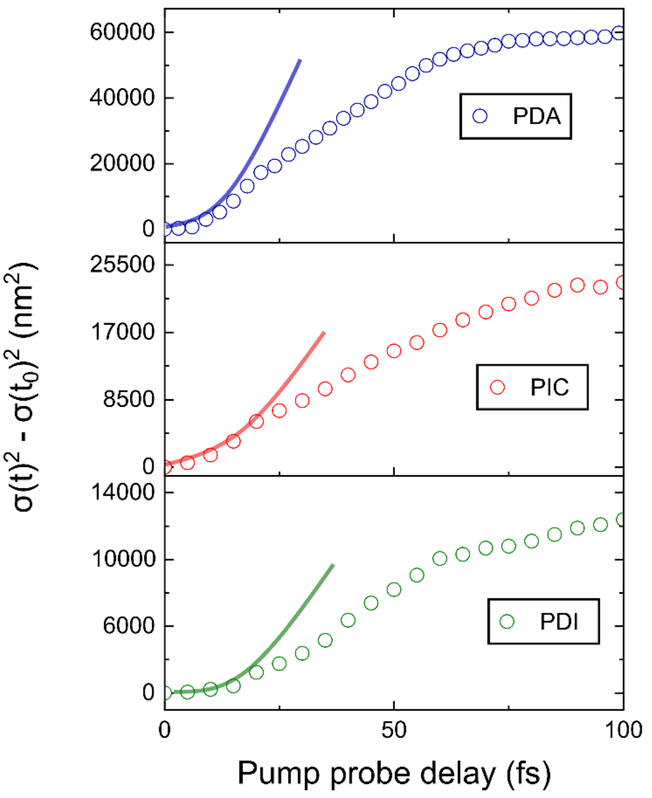

Supplementary Figure 73: Mean square displacement for PDA, PIC and PDI showing a quadratic fit to the initial time points. This is in keeping with the proposed ballistic transport of the initial polariton wavefuction. However, we refrain from extracting a diffusion coefficient from the data in this region as it is close to the instrument response of the setup and unphysical.

Supplementary Note 11: Theoretical modelling of exciton diffusion in PDI, PIC and PDA

Ab initio calculations of lowest electronic transitions and transition densities of PDI molecules

Structure of a single PDI molecule, Supplementary Figure 74a, was optimized using DFT as implemented in Turbomole 6.0 [30]. For the structure optimization, we used the B3LYP hybrid functional [31] and the triple-ζ def2-TZVP basis set [32] that provide a reasonable balance between the precision of the results and computation time. Five lowest singlet electronic excitations of PDI molecule were computed using TDDFT with the same functional and the basis set. The lowest electronic excitation is optically active with the corresponding transition dipole about $\mu = 9.2$ Debye and the transition frequency $\hbar\omega = 2.37\text{eV}$. The computed frequency agrees well with the frequency measured experimentally in a solution of PDI molecules, see Supplementary Figure 74b-c. This transition is dominated by the HOMO → LUMO. The next four electronic transitions are separated from the lowest one by the gap of the order of 0.8 eV and are optically dark, see dashed lines in Supplementary Figure 74b.

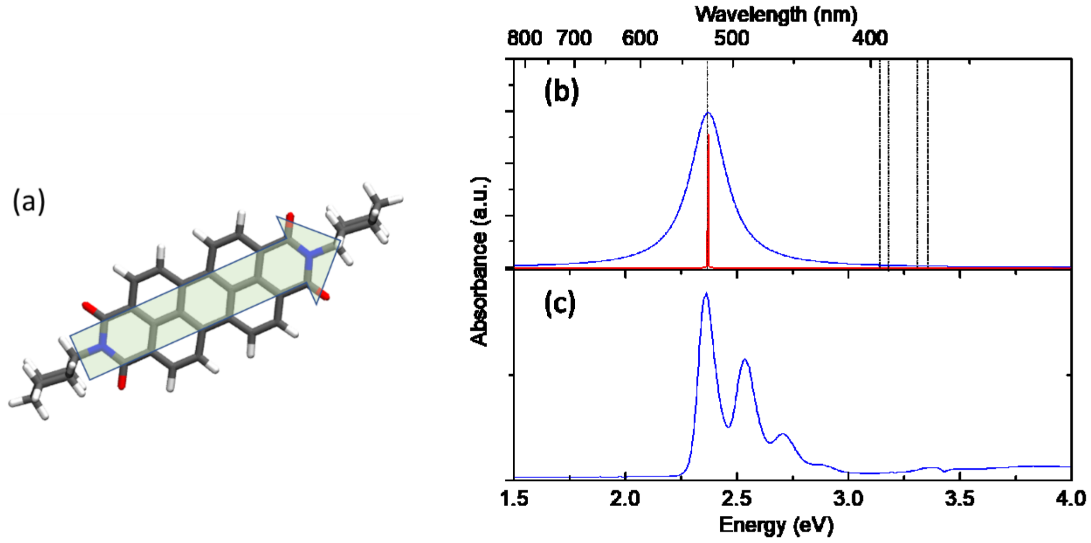

Supplementary Figure 74: a. Structure of PDI molecule and the orientation of the transition dipole associated with the lowest electronic excitation. **b.** Computed electronic excitation spectrum of PDI molecule. Frequencies of the lowest five transitions are marked by dashed lines. Only the lowest electronic transition is optically active. The red line corresponds to the computed frequency and the blue line is for the Lorentz-broadened transition with $\Gamma = 50$ meV. **c.** Measured absorption spectrum of PDI molecules in solution showing vibronic progression.

Intermediate couplings and exciton transport in PDI nanobelts

The transport of singlet excitons in PDI nanocrystals is controlled by the Förster coupling between the intramolecular electronic transitions [33]. We compute the Förster coupling between PDI molecules using the electron transition densities obtained from DFT calculations. Supplementary Figure 75a shows the coupling strength in a dimer of PDI molecules as a function of the relative angle between two molecules displaced axially by the Van der Waals distance, which is equal to 3.62 A. Supplementary Figure 75b shows the distance dependence of the interaction while the relative angle is fixed at $\varphi = 30°$. In order to efficiently compute excitonic Hamiltonian of nanocrystals, we fit the a extended dipole model [34] to the intermolecular coupling dependencies obtained using transition densities. The resulting parameters of extended dipoles are $q = 0.25e$ and $l = 7.6$ Å.

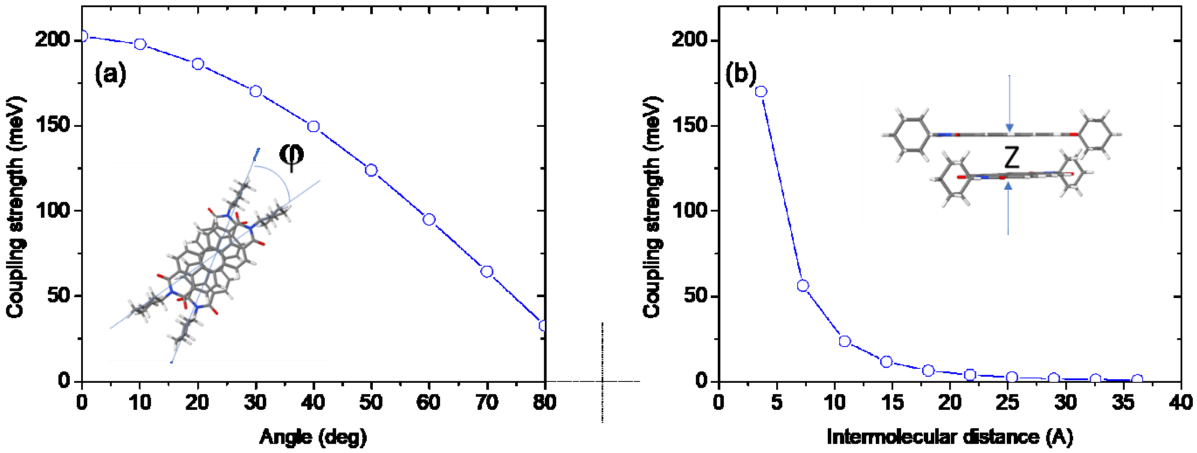

Supplementary Figure 75: Computed excitonic coupling strength in a dimer of PDI molecules as a function of the relative angle **a** and the intermolecular distance **b**.

To model absorption spectra and exciton dynamics in PDI nanocrystals, we built three slabs that represent 3-dimensional, 2-dimensional, and 1-dimensional domains. The slabs are composed correspondingly of 3×3×17 lattice cells (1224 molecules), 13×13×1 lattice cells (1352 molecules), and 1×1×151 lattice cells (1208 molecules). Then, the excitonic Hamiltonians were constructed with the values of extended transition dipoles obtained from the fitting procedure and the electronic excitation spectra were computed. The theoretical spectra of the slabs, Supplementary Figure 75a-c, are consistent with the measured absorption spectrum of PDI nanobelts, Supplementary Figure 75d. Firstly, in all computed spectra, the majority of the bright excitonic transitions are shifted to shorter wavelengths (blue part of the spectrum) as compared to the transition in monomers. Secondly, the ranges of the shift in the theory and experiment are comparable. However, in our computational model the values of the shifts depend sufficiently on the shape of the slab. This is due to the saturation of the Förster interaction

in stacks of PDI molecules. Finally, all spectra show a feature red-shifted from the monomer peak by about 100-150 meV. It is likely that the experimental spectrum shows responses from multiple domains of different shapes. We conclude that the derived Hamiltonian can be used for further modelling of exciton transport.

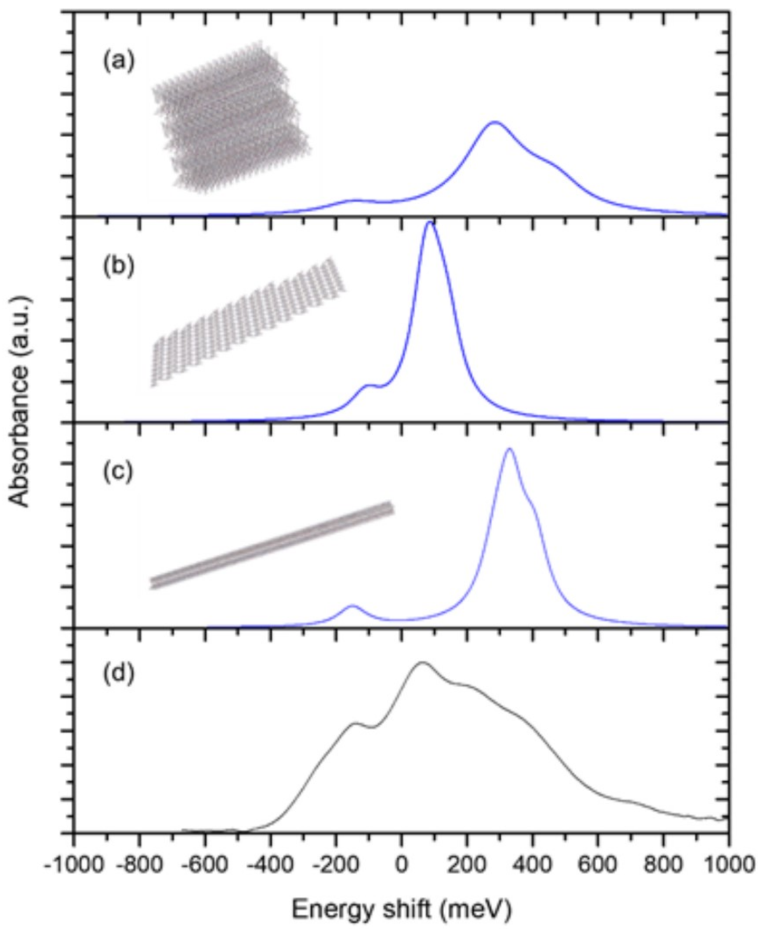

Supplementary Figure 76: a-c. Computed spectra of 3D, 2D, and 1D PDI slabs. The relative intensities are normalized to the number of molecules. The transition lines are broadened using a Lorentzian function with $\Gamma = 50$ meV. **d.** Measured absorption spectrum of PDI nanobelts.

The diffusion coefficient for singlet excitons in PDI crystals has been estimated using the model developed by Haken, Reineker, and Strobl [35,36], where the exciton scattering is modelled using a phenomenological source of white noise. This model provides realistic estimates of transport properties, both ballistic and diffusive, at high temperatures keeping the number of model parameters minimal. Specifically, in the model we include a single phenomenological parameter, Γ, which characterizes the exciton dephasing rate due to the fluctuation of intramolecular electronic transition frequencies. For perfect molecular crystals, the second moment of the exciton wave function can be written as [37]:

$$M_2(t) = \frac{\sum_{m>0}(x_m V_m)^2}{\Gamma}\left\{\frac{e^{-2\Gamma t/\hbar}-1}{\Gamma} + \frac{2t}{\hbar}\right\}, (19)$$

where V_m is the strength of excitonic couplings between two molecules, x_m is the intermolecular distance, and the sum is taken over only positive direction. At short times $t \ll \hbar/\Gamma$ the exciton transport is ballistic with the velocity $v \propto \sum_{m>0} x_m V_m/\hbar$, and it becomes diffusive at longer time with the diffusion coefficient $D = \sum_{m>0}(x_m V_m)^2/(\hbar\Gamma)$. In the derived model, the intermolecular couplings along the $\pi - \pi$ stacked chains are several times stronger than the interactions between molecules belonging to different chains. Therefore, for the sake of simplicity we estimate 1D transport along single chains.

The computed ballistic velocity of excitons $v \propto 10$ m/sec, which is about 30 times smaller than the value estimated from the experiments in the R_1 region. In order to have the diffusion coefficient the same as the experimentally measured in R_2 region, the dephasing rate should be about $\Gamma = 5$ meV. While this value is realistic, it disagrees with the measured line widths that are of the order of $\Gamma = 50$ meV. One potential reason for this discrepancy is that in the R_2 region excitons still have some small admixture of a photon mode. In this case, the diffusion can be fast even for large values of Γ. Another potential reason is that a large fraction of the line width in the measured absorption spectra is due to the inhomogeneous broadening.

Finally, we compute exciton dynamics in the three slabs shown in Supplementary Figure 77a-c. In the model we assume the dephasing rate $\Gamma = 5$ meV. Additionally, we include a disorder in molecular transition frequencies with a Gaussian distribution of $\Delta\omega = 50$ meV width. Supplementary Figure 78 shows the second moments of the exciton wave function as a function of time for three slabs. Each simulation is averaged over 100 different realizations of structural disorder. At time $t = 0$ the excitation was localized on a single molecule in the middle of the slab. The initial dynamics of the exciton wave packet is coherent with a ballistic propagation that is reflected as a quadratic time dependence of the second moment. At longer times, the exciton dynamics becomes diffusive with a linear time dependence. The slab boundaries limit the transport of excitons which can be seen for the all three slabs. Only 1D slab is sufficiently long that the boundaries do not affect the exciton dynamics on the time scales of 100 fs. In this case the diffusion coefficient along the stack of PDI molecules, z-axis, is equal to $D = 370$ Å^2/fs, see Supplementary Figure 74c, that is comparable to the value obtained in the experiments.

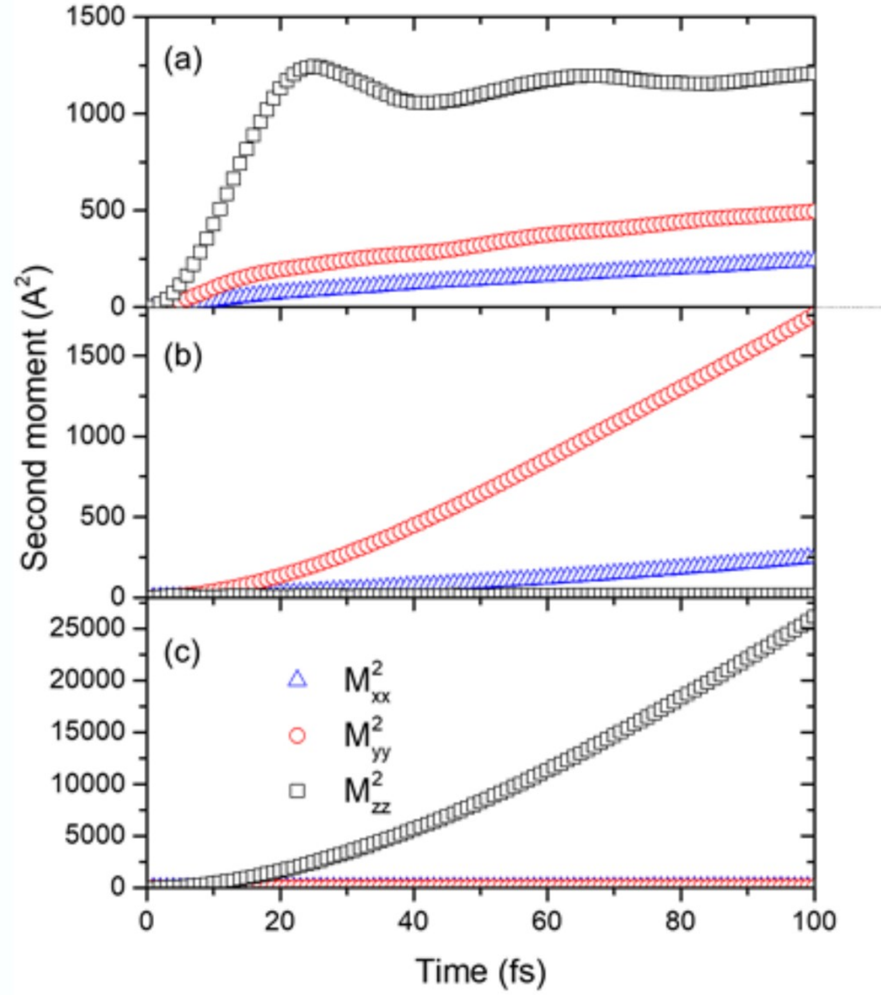

Supplementary Figure 77: a-c. Computed dynamics of excitons in **a.** 3D, **b.** 2D, and **c.** 1D slabs of PDI molecules. The plots show second moments of the exciton wave function (z-axis is along the stacks of PDI molecules) as a function of time for the first 100 femtoseconds. The dephasing rate is Γ = 5 meV.

In our model the ballistic velocity is proportional to $\frac{\mu}{X^2}$ where μ is the transition dipole moment and X is the intermolecular separation. Variations in X are due to the uncertainty in the X-ray measurements (~2%) and the thermal expansion, which is ~1×10^4 K^{-1} for organic crystals. This equates to a total uncertainty in the intermolecular distance of ~10%. The uncertainty in the transition dipole is more difficult to estimate as it is not directly measurable. However, estimating this to be ~15-20% as reported in e.g. Tamai *et al.* [38] gives an overall ~30% uncertainty in the velocity.

PIC has a smaller transition dipole as compared to PDI, it's 10D for PIC versus 13D for PDI. If we assume the same coupling strength and the exciton velocity the expected exciton velocity should only be only 60% of that observed for PDI at ~0.12×10^{6} ms^{-1}. The uncertainty should be the same ~30%. In our experiments we observe faster transport in PIC as compared to PDI. This could be due to stronger coupling between PIC molecules as compared to PDI i.e. a different molecular arrangement as well as there also being a stronger admixture of a photon modes in PIC aggregates. The former point will certainly be true. Despite over a 90 years of research the exact packing of PIC molecules in their aggregates has still not yet been determined [39], with several potential models proposed. For this reason, a full calculation of the maximum exciton velocity that would be expected has not been performed here for this system.

Theoretical calculation of exciton velocities in PDA

In order to obtain the exciton group velocity of PDA, we calculate the excitonic band structure using GW-BSE methods [40,41], as implemented in the YAMBO code [42]. Due to the large computational cost associated with these calculations, and the necessity to study a supercell of PDA to obtain the velocity (see below), we restrict ourselves to a model PDA system, which only contains the conjugated structure (within which exciton transport occurs) and the side-chains have been substituted by hydrogen atoms. This structure is obtained from a realistic PDA structure as deposited in the Cambridge Crystallographic Database (identifier: DEMYID), when we truncate the side-chains, but keep the rest of the structure to be identical to the real one.

The group velocity within the first Brillouin zone of the periodic conjugated system is approximated as:

$$v_g = \frac{1}{\hbar} \cdot \frac{E(X) - E(\Gamma)}{q(X) - q(\Gamma)}, \quad (20)$$

where E is the exciton energy and q the exciton wavevector. Here we denote $q(\Gamma) = (0,0,0)$ the zone-centre, and $q(X) = (\frac{2\pi}{\alpha}, 0,0)$ the zone-edge in the x-direction, along which the PDA chains lie. In order to obtain $E(X)$, we generate a supercell of size $2 \times 1 \times 1$, thus halving the size of the first Brillouin zone. As a result, the zone-boundary X point folds onto Γ, which appears as an extra exciton state once the BSE Hamiltonian of the $2 \times 1 \times 1$ system is diagonalised. The distance of this new band from the exciton value at Γ gives the width of the exciton band, which we find to be equal to 1.33 eV. The lattice

constant of the studied PDA is $\alpha = 4.88$ Angstrom, yielding a group velocity of 0.3×10^6 m s^{-1}. This is likely an overestimate of the exciton ballistic velocity in PDA, as the calculation assumes a perfectly periodic and disorder-free structure. Therefore, it is clear that the exciton ballistic velocity in this material is at least an order of magnitude slower compared to the regime of ballistic transport (R_1) as observed in the TAM.

For performing energy self-consistent GW calculations we find that using 40 Kohn-Sham states, 40 states for the polarisation function calculation, and a 3 Ry cut-off for the dielectric matrix, leads to converged results for the fundamental gap. For the exciton energy, we solve the Bethe-Salpeter equation (BSE), using 4 occupied and 4 unoccupied bands, and setting the exchange term to 40 Ry, which converges the first exciton peak. Due to the one-dimensional character of studied PDAs, divergences occur at small values of q (i.e. at long distances) in the Coulomb term appearing in the calculations. To avoid this problem, we employ the random integration method, as implemented in Yambo. The calculations are converged by using 10^5random q-points in the first Brillouin zone, and setting the cut-off for the real space components of the Coulomb interaction to 20 Ry.

Supplementary Note 12: Waveguiding

The rapid initial growth described in the main text could be argued to result from the waveguiding properties of aggregate 1D crystals, which propagate light efficiently away from the initial excitation spot. Such light can be absorbed along the fiber- or wire-shaped organic waveguides and produce an excited population, which becomes visible in our TAM data.

Balzer *et al.* studied the propagating light modes in self-assembled organic needle-like aggregates which have rectangular cross sections, on a dielectric substrate [43,44]. They demonstrated that such waveguides only support transverse magnetic modes (TM modes) when the fibres are assumed to be optically uniaxial with the dielectric tensor components $\epsilon_{\parallel}$ (along the fibre) and $\epsilon_{\perp}$ (perpendicular to the fibre). The number of possible modes, m, is restricted by:

$$m < \frac{2a}{\lambda}\sqrt{\frac{\epsilon_{\perp}}{\epsilon_{\parallel}}}\sqrt{\epsilon_{\parallel} - \epsilon_S} \quad (21)$$

where a is the width of the waveguide, λ is the wavelength of the propagating light, and ϵ_S is the dielectric constant of the substrate. From our ellipsometry measurements and published data, $n_{\perp}$ was determined to be ~1.60 for PIC [39] and PDA [45], and 1.62 for PDI [46]. By further assuming that all three systems are optically isotropic, we adopted $\epsilon_{iso} = n_{\perp}^2$ as the value for the isotropic dielectric constant. The calculated values of m for each of the three systems are summarised in the table below, using the value $\epsilon_S = n_S^2 = 1.53^2 = 2.34$ for the glass coverslip used in our TAM measurements.

	PIC	PDA	PDI
a / µm	1	2	0.13
ϵ_{iso}	2.56	2.56	2.62
λ_{pump} / nm	565	590	590
m	1.66	3.18	0.23

Supplementary Table 3: Table summarising refractive index and supported waveguiding modes for three materials considered.

This mode analysis suggests that only m = 1 is possible in PIC whereas m up to 3 is supported in PDA. PDI, on the other hand, is unable to support any waveguides due to its dimensions and this has been shown to be the case at room temperature experimentally [46].

It can be shown that the wavevector k and the group velocity v_g of the supported waveguiding mode are given by:

$$k = \sqrt{\frac{\omega^2}{c^2}\epsilon_{\parallel} - \frac{\epsilon_{\parallel}}{\epsilon_{\perp}}\left(\frac{m\pi}{a}\right)^2} \quad (22)$$

$$v_g = \frac{d\omega}{dk} = \frac{c^2 k}{\omega \epsilon_{\parallel}} \quad (23)$$

The calculated group velocities plotted in Figure **SI78** show a minimum velocity of 0.5c for any supported modes in PIC and PDA, which is at least 60 times faster than the maximum propagation speed observed in our experiments (i.e. 1% of light speed in vacuum). This suggests that the observed movement is not the result of waveguiding of the pump pulse and subsequent absorption along the length of the crystals.

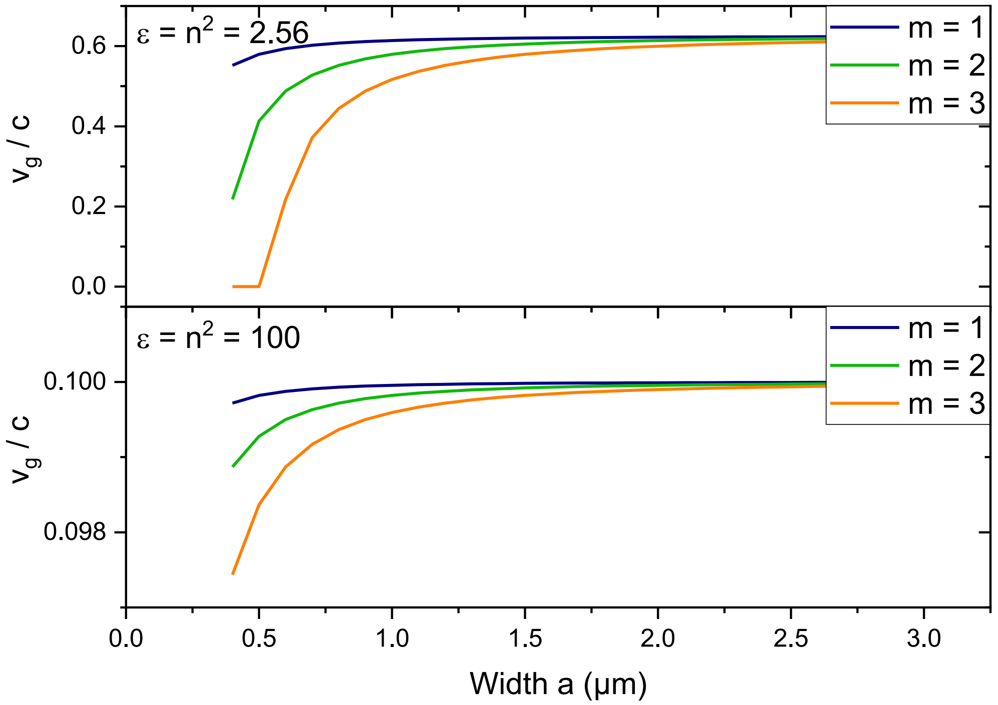

Supplementary Figure 78: Calculated group velocity for indicated waveguiding modes for PIC (top) and PDA (bottom).

It has been reported that exciton-polaritons (EP) could be formed by coupling between propagating fluorescence and the excitons within nanofibers. The authors observed a refractive index parallel to the nanofibers exceeding 10 at energies close to the anti-crossing point of the lower EP branch. This unusually high refractive index would result in a much slower group velocity. However, even with n = 10, the calculated group velocities (0.1c) is still about an order of magnitude higher than our observations. Therefore, it seems reasonable to rule out the possibility of waveguiding and argue that the observed expansion in our TAM signal is the result of ultrafast electronic population transport in our nanostructures. Nonetheless, weak EP coupling to the supported wave guiding modes could theoretically increase the group velocity. Further studies, theoretically and experimentally, is required to elucidate the nature of the transport mechanism.

Supplementary Note 13: Control measurements

In order to confirm that the ultrafast, ultra-long energy propagation described in the main text arises from the specific chemical and morphological nature of the samples control measurements were performed on the isolated PIC, PDI dye monomers as well as amorphous poorly polymerised regions of the PDA sample. Additional measurements were also performed on diluted films of CdSe nanocrystals. In all cases no (ultrafast) energy propagation could be observed, confirming the behaviour observed in the main text is unique to the chemical structure of the materials and does not arise from a systematic measurement artefact.

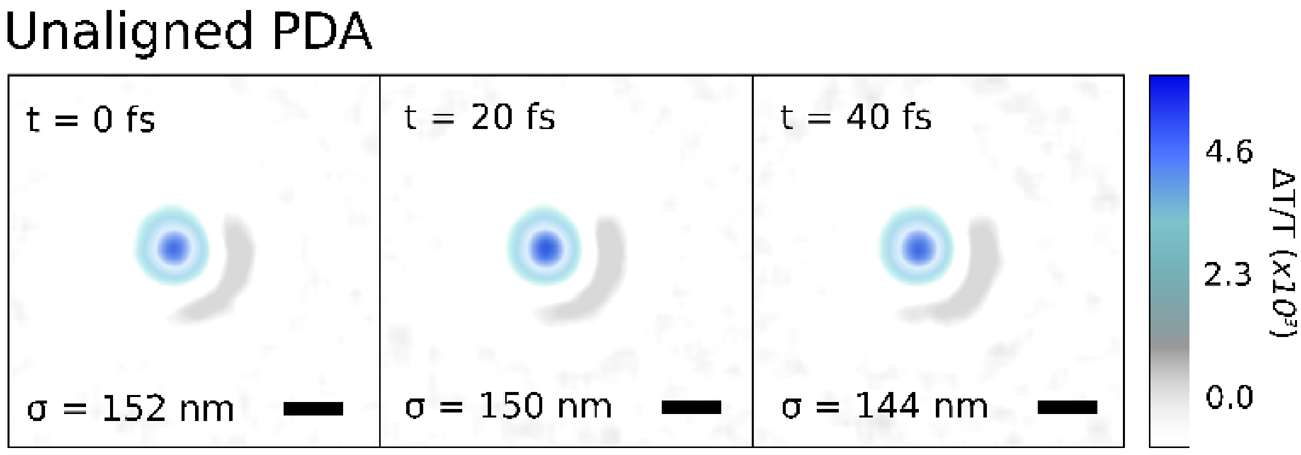

Supplementary Figure 79: fs-TAM images unaligned poorly polymerized PDA (Supplementary Figure 14c) following photoexcitation. The 10-fs pump pulse is at 520-650 nm, and probing is carried out at 670 nm at the edge of the presumed stimulated emission band. The scale bar is 500 nm and the Gaussian standard deviation (σ) is indicated in the bottom left corner of the images.

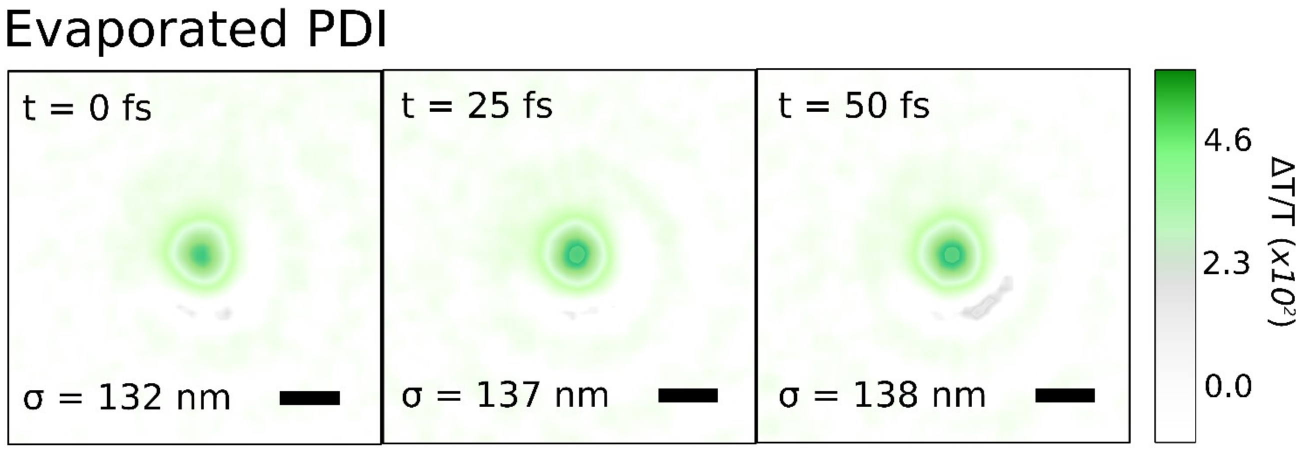

Supplementary Figure 80: fs-TAM images of an evaporated thin film (100 nm thickness) of PDI following photoexcitation. The 10-fs pump pulse is at 520-650 nm, and probing is carried out at 780 nm in the singlet photo-induced absorption band. The scale bar is 500 nm.

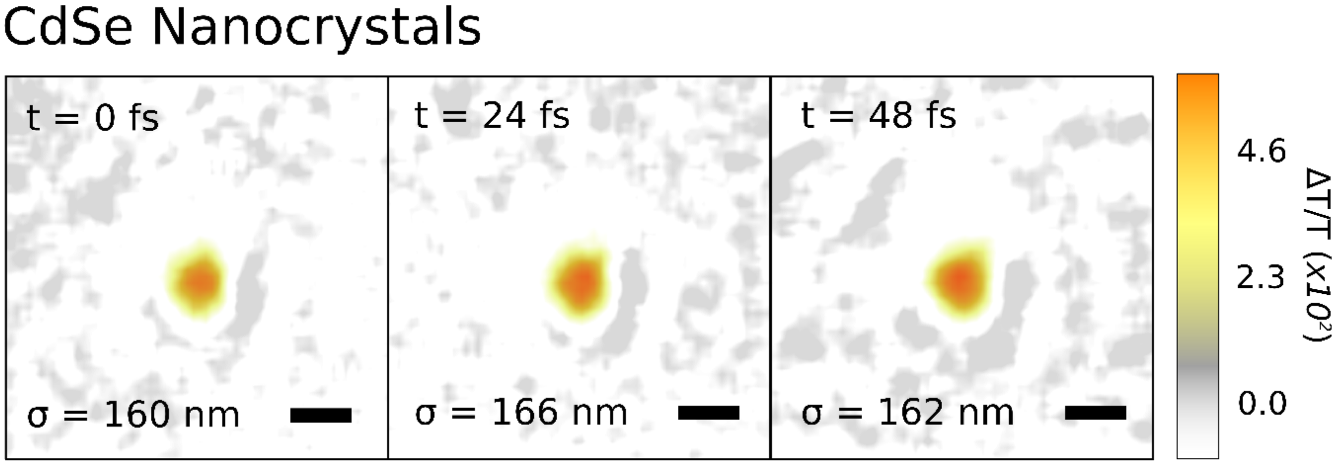

Supplementary Figure 81: fs-TAM images of CdSe nanocrystals dispersed 2% w/v in a polystyrene matrix (M_w ~250, 000; film thickness 150 nm) following photoexcitation. The 10-fs pump pulse is at 520-650 nm, and probing is carried out at 700 nm in the broad stimulated emission band. The scale bar is 500 nm.

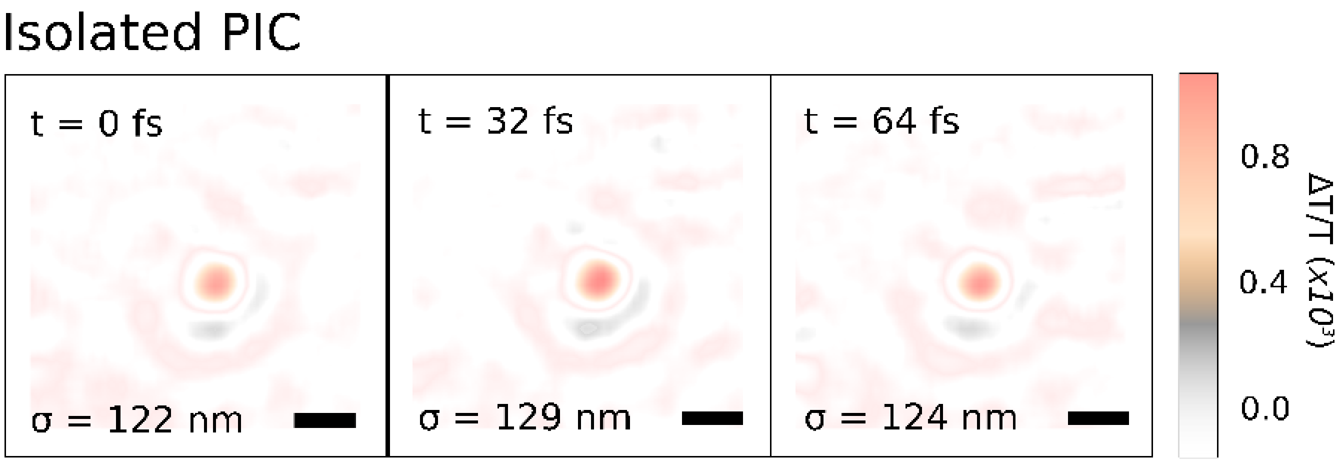

Supplementary Figure 82: fs-TAM images of PIC dye monomers dispersed 1% w/v in a polystyrene matrix (~250, 000 M_w; film thickness 130 nm) following photoexcitation. The 10 fs pump pulse is at 520-650 nm, and probing is carried out at 660 nm in the photo-induced absorption band. The scale bar is 500 nm.

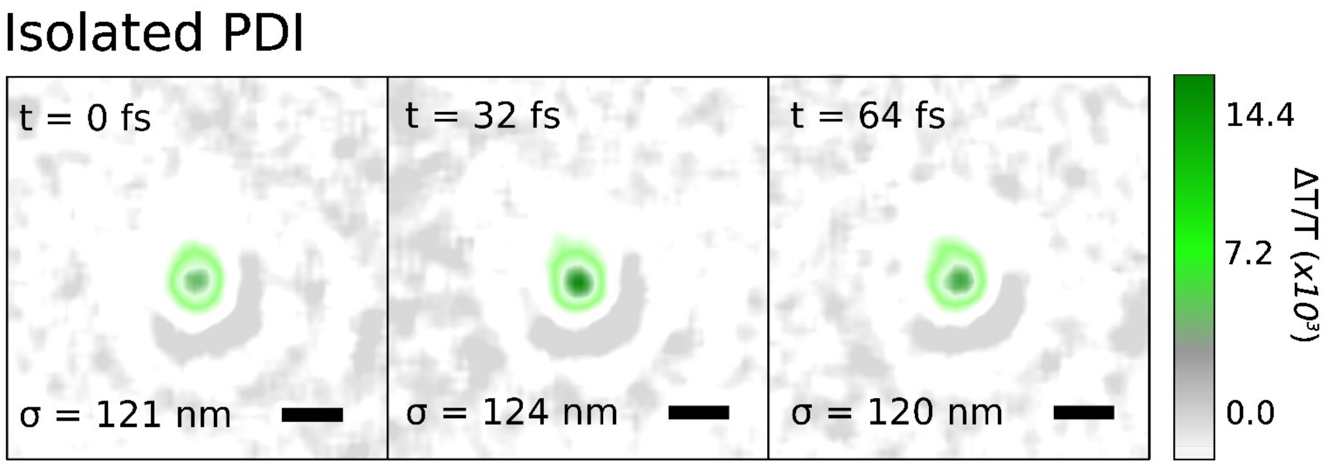

Supplementary Figure 83: fs-TAM images of PDI dye monomers dispersed 1% w/v in a PMMA matrix (M_w ~500, 000; film thickness 200 nm) following photoexcitation. The 10-fs pump pulse is at 520-650 nm, and probing is carried out at 770 nm in the photo-induced absorption band. The scale bar is 500 nm.

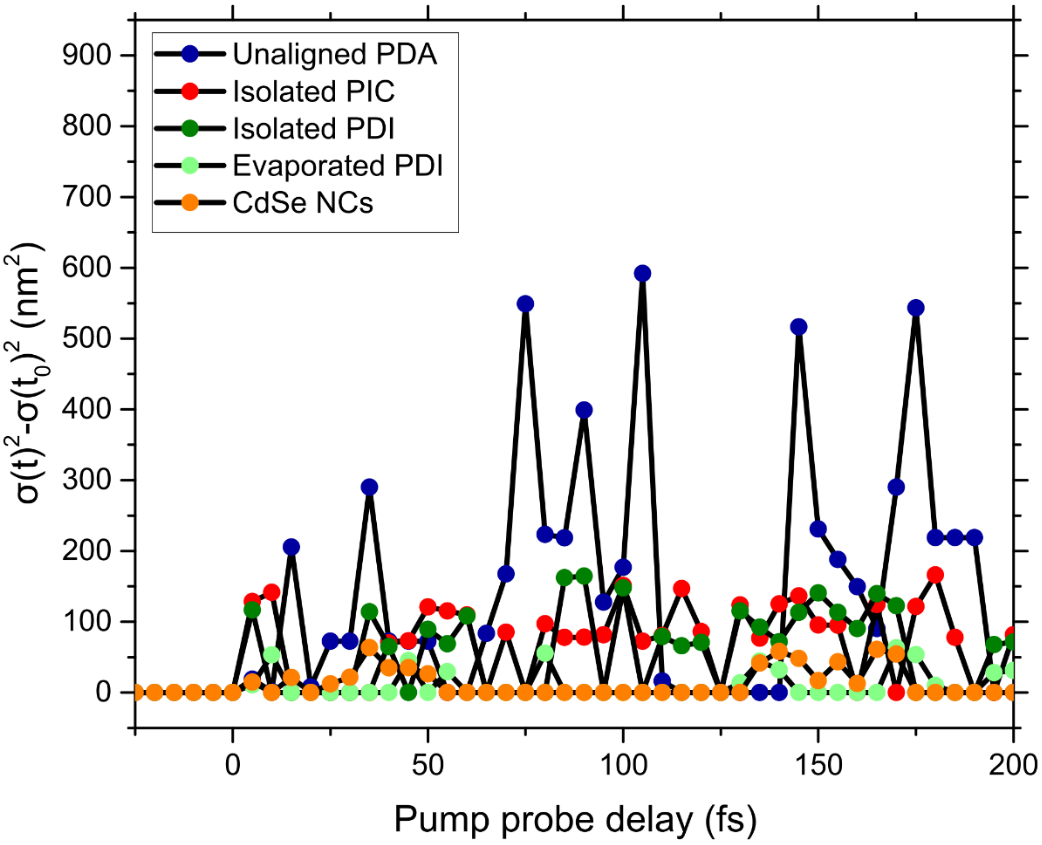

Supplementary Figure 84: Mean square displacement for control samples shown above. In all cases there appears to be no expansion and the radial extent of the spatial $\Delta T/T$ fluctuates about the value obtained at t_0. In the case when, $\sigma(t)^2$ drops below $\sigma(t_0)^2$ we set by convention set the mean square to 0 for ease of viewing. Any spikes in growth are assigned to random noise fluctuations in the relatively low signals.

Performing reflectivity measurements on PIC samples prepared from solutions of varying dilution (see below) shows that as the ratio of matrix to nanotubes is increased no splitting is observed in the reflectivity spectrum. Furthermore, in these more dilute nanotube-matrix composites no ultrafast transport is observed. This supports the hypothesis that regions of locally enhanced oscillator strength are responsible for the peak splitting reported in Figure 1 of the main text. These regions of locally enhanced oscillator strength in turn give rise to the EP formation and ultrafast transport.

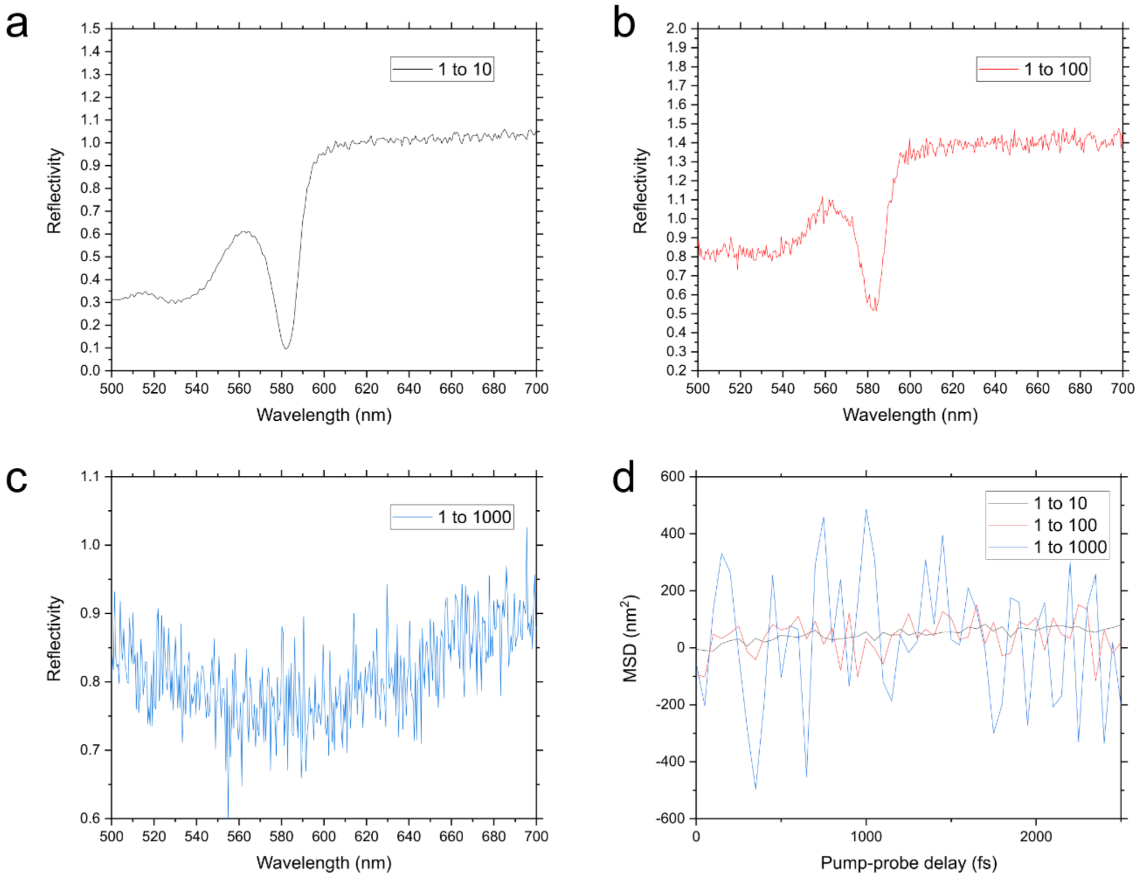

Supplementary Figure 85: a-c. Reflectivity spectrum of PIC nanotubes prepared in glassy matrices with differing ratios of sugar matrix solution to nanotube solution (by volume). In these more dilute films no splitting is observed in the reflectivity. **d.** MSD from fs-TAM experiments performed on PIC solutions of differing matrix to nanotube ratio. In all cases no ultrafast transport akin to that reported in the main text is observed.

Supplementary Note 14: Refractive index dependent fs-TAM measurements

In Figure 5 of the main text we present fs-TAM measurements where the refractive of the optical environment surrounding the organic material is tuned. In the case of PDA and PIC a ~300 nm thick film of PMMA (Sigma; 10% wt in Toluene) was spun atop of the dry organic film in an inert environment. The samples were then encapsulated as previously described. Performing UV-Vis measurements before and after applying the polymer showed it did not degrade the organic material in any way. For the case of PDI, PMMA was found to be unsuitable for polymer encapsulation due to the solubility requirements of nanobelts and the polymer; in this case an alternate polymer PDMS (Sigma; M_n ~1200) was used for encapsulation. A thin film (~700 nm thickness) of PDMS was spun atop of the nanobelts and encapsulated as before. For covering the samples with index matching oil (Olympus MOIL-30; refractive index ~1.53), the organic material was sandwiched with a second coverslip (gap of ~900 nm.) Oil was then pipette into the gap between the two coverslips such that it covered the entirety of the film.

Supplementary References

1. Trebino, R. *et al.* Measuring Ultrashort Laser Pulses in the Time-Frequency Domain Using Frequency-Resolved Optical Gating. *Rev. Sci. Instrum.* **68**, 3277–3295 (1997).

2. Kazunori, S., Ohnuma, H. & Kotaka, T. Urethane Substituted Polydiacetylene: Synthesis and Characterization of Poly[4,6-decadiyn-1,10-diol-bis(n-butoxy-carbonyl-methyl-urethane)]. *Polym. J.* **14**, 895–905 (1982).

3. Ribierre, J.-C. *et al.* A solvent-free and vacuum-free melt-processing method to fabricate organic semiconducting layers with large crystal size for organic electronic applications. *J. Mater. Chem. C* **7**, 3190–3198 (2019).

4. Vierheilig, A. *et al.* Femtosecond dynamics of ground-state vibrational motion and energy flow: Polymers of diacetylene. *Chem. Phys. Lett.* **312**, 349–356 (1999).

5. Horvath, A., Weiser, G., Lapersonne-Meyer, C., Schott, M. & Spagnoli, S. Wannier excitons and Franz-Keldysh effect of polydiacetylene chains diluted in their single crystal monomer matrix. *Phys. Rev. B - Condens. Matter Mater. Phys.* **53**, 13507–13514 (1996).

6. Kraabel, B., Joffre, M., Lapersonne-Meyer, C. & Schott, M. Singlet Exciton Relaxation in Isolated Polydiacetylene Chains Studied by sub-picosecond Pump-Probe Experiments. *Phys. Rev. B* **58**, (1998).

7. Lanzani, G. *Photophysics of Molecular Materials: From Single Molecules to Single Crystals. Photophysics of Molecular Materials: From Single Molecules to Single Crystals* (Wiley-Blackwell, 2006).

8. Spagnoli, S., Schott, M., Johnson, M. & Toupet, L. Structural study of two reactive diacetylenes. *Chem. Phys.* **333**, 236–245 (2007).

9. Brito de Barros, R. & Ilharco, L. M. The role of cellulose acetate as a matrix for aggregation of pseudoisocyanine iodide: Absorption and emission studies. *Spectrochim. Acta - Part A Mol. Biomol. Spectrosc.* **57**, 1809–1817 (2001).

10. von Berlepsch, H., Böttcher, C. & Dähne, L. Structure of J-Aggregates of Pseudoisocyanine Dye in Aqueous Solution. *J. Phys. Chem. B* **104**, 8792–8799 (2002).

11. Caram, J. R. *et al.* Room-Temperature Micron-Scale Exciton Migration in a Stabilized Emissive Molecular Aggregate. *Nano Lett.* **16**, 6808–6815 (2016).

12. Che, Y., Yang, X., Balakrishnan, K., Zuo, J. & Zang, L. Highly Polarized and Self-Waveguided Emission from Single-Crystalline Organic Nanobelts. *Chem. Mater.* **21**, 2930–2934 (2009).

13. Kabsch, W. *XDS. Acta Crystallogr. Sect. D Biol. Crystallogr.* **66** (2010).

14. Sheldrick, G. M. Crystal structure refinement with SHELXL. *Acta Crystallogr. Sect. C, Struct. Chem.* **71**, 3–8 (2015).

15. Sheldrick, G. M. SHELX. *Acta Cryst. A* **64**, 112–122 (2008).

16. Palatinus, L. *et al.* Structure refinement using precession electron diffraction tomography and dynamical diffraction: Tests on experimental data. *Acta Crystallogr. Sect. B Struct. Sci. Cryst. Eng. Mater.* **71**, 740–751 (2015).

17. Heijs, D. J., Malyshev, V. A. & Knoester, J. Thermal broadening of the J-band in disordered linear molecular aggregates: A theoretical study. *J. Chem. Phys.* **123**, 1445071–1445083 (2005).

18. Grant, R. T. *et al.* Strong coupling in a microcavity containing β-carotene. *Opt. Express* **26**, 3320 (2018).

19. Hopfield, J. J. & Thomas, D. G. Polariton Absorption Lines. *Phys. Rev. Lett.* **15**, 22–25 (1965).

20. Morris, G. C. & Sceats, M. G. The 4000 Å transition of crystal anthracene. *Chem. Phys.* **3**, 164–179 (1974).

21. Turlet, J. M., Kottis, P. & Philpott, M. R. Polariton and Surface Exciton State Effects in the Photodynamics of Organic Molecular Crystals. Polariton and Surface Exciton State Effects in the Photodynamics of Organic Molecular Crystals (Wiley, 2007).

22. Oskooi, A. F. *et al.* Meep: A flexible free-software package for electromagnetic simulations by the FDTD method. *Comput. Phys. Commun.* **181**, 687–702 (2010).

23. Chutinan, A. & Noda, S. Waveguides and waveguide bends in two-dimensional photonic crystal slabs. *Phys. Rev. B - Condens. Matter Mater. Phys.* **62**, 4488–4492 (2000).

24. Cacciola, A. *et al.* Subdiffraction Light Concentration by J-Aggregate Nanostructures. *ACS Photonics* **2**, 971–979 (2015).

25. Mack, C. Fundamental Principles of Optical Lithography: The Science of Microfabrication. Fundamental Principles of Optical Lithography: The Science of Microfabrication (Wiley, 2007).

26. Ribeiro, R. F., Martínez-Martínez, L. A., Du, M., Campos-Gonzalez-Angulo, J. & Yuen-Zhou, J. Polariton Chemistry: Controlling Molecular Dynamics with Optical Cavities. *Chem. Sci.* **9**, 6325–6339 (2018).

27. Zhu, T., Wan, Y. & Huang, L. Direct Imaging of Frenkel Exciton Transport by Ultrafast Microscopy. *Acc. Chem. Res.* **50**, 1725–1733 (2017).

28. Wan, Y., Wiederrecht, G. P., Schaller, R. D., Johnson, J. C. & Huang, L. Transport of Spin-Entangled Triplet Excitons Generated by Singlet Fission. *J. Phys. Chem. Lett.* **9**, 6731–6738 (2018).

29. Wan, Y., Stradomska, A., Knoester, J. & Huang, L. Direct Imaging of Exciton Transport in Tubular Porphyrin Aggregates by Ultrafast Microscopy. *J. Am. Chem. Soc.* **139**, 7287–7293 (2017).

30. Ahlrichs, R., Bär, M., Häser, M., Horn, H. & Kölmel, C. Electronic structure calculations on workstation computers: The program system turbomole. *Chem. Phys. Lett.* **162**, 165–169 (1989).

31. Becke, A. D. Density-functional thermochemistry. III. The role of exact exchange. *J. Chem. Phys.* **98**, 5648–5652 (1993).

32. Schäfer, A., Horn, H. & Ahlrichs, R. Fully optimized contracted Gaussian basis sets for atoms Li to Kr. *J. Chem. Phys.* **97**, 2571–2577 (1992).

33. Förster, T. *Chapter III: Modern Quantum Chemistry*. (Dove 1965).

34. Kobayashi, T. *J-Aggregates*. (Worl Scientfic, 1996).

35. Haken, H. & Strobl, G. An exactly solvable model for coherent and incoherent exciton motion. *Zeitschrift für Phys.* **262**, 135–148 (1973).

36. Haken, H. & Reineker, P. The coupled coherent and incoherent motion of excitons and its influence on the line shape of optical absorption. *Zeitschrift für Phys.* **249**, 253–268 (1972).

37. Reineker, P. Equations of motion for the moments of the coupled coherent and incoherent

motion of triplet and singlet excitons. *Zeitschrift für Phys.* **261**, 187–190 (1973).

38. Tamai, Y., Ohkita, H., Benten, H. & Ito, S. Exciton Diffusion in Conjugated Polymers: From Fundamental Understanding to Improvement in Photovoltaic Conversion Efficiency. *J. Phys. Chem. Lett.* **6**, 3417–3428 (2015).

39. Von Berlepsch, H., Möller, S. & Dähne, L. Optical properties of crystalline pseudoisocyanine (PIC). *J. Phys. Chem. B* **105**, 5689–5699 (2001).

40. Rohlfing, M. & Louie, S. G. Electron-hole excitations in semiconductors and insulators. *Phys. Rev. Lett.* **81** (1998).

41. Hedin, L. New method for calculating the one-particle Green’s function with application to the electron-gas problem. *Phys. Rev.* **139** (1965).

42. Marini, A., Hogan, C., Grüning, M. & Varsano, D. yambo: An ab initio tool for excited state calculations. *Comput. Phys. Commun.* **180** (2009).

43. Balzer, F., Bordo, V. G., Simonsen, A. C. & Rubahn, H.-G. Optical waveguiding in individual nanometer-scale organic fibers. *Phys. Rev. B* **82**, (2003).

44. Balzer, F., Bordo, V. G., Simonsen, A. C. & Rubahn, H.-G. Isolated hexaphenyl nanofibers as optical waveguides. *Appl. Phys. Lett.* **67**, 115408 (2003).

45. Sauteret, C. *et al.* Optical nonlinearities in one-dimensional-conjugated polymer crystals. *Phys. Rev. Lett.* **36**, 956–959 (1976).

46. Chaudhuri, D. *et al.* Enhancing Long-Range Exciton Guiding in Molecular Nanowires by H-Aggregation Lifetime Engineering. *Nano Lett.* **11**, 488–492 (2011).
